# Evaluation of mortality among Marines, Navy personnel, and civilian workers exposed to contaminated drinking water at USMC base Camp Lejeune: a cohort study

**DOI:** 10.1186/s12940-024-01099-7

**Published:** 2024-07-03

**Authors:** Frank J. Bove, April Greek, Ruth Gatiba, Rona C. Boehm, Marcie M. Mohnsen

**Affiliations:** 1 Office of Community Health and Hazard Assessment, Health Studies Section, Agency for Toxic Substances and Disease Registry (ATSDR)/CDC, 6558 Parkside Way, Chamblee, GA 30084 USA; 2 Health Research and Analytics Division, Battelle Memorial Institute, Charlottesville, VA USA; 3 Battelle Memorial Institute, Columbus, OH USA

**Keywords:** Camp Lejeune, Camp Pendleton, Marines/Navy personnel, Civilian workers, Mortality, Drinking water, Trichloroethylene, Tetrachloroethylene, Benzene, Vinyl chloride, Hazard ratio

## Abstract

**Background:**

Drinking water at U.S. Marine Corps Base (MCB) Camp Lejeune, North Carolina was contaminated with trichloroethylene and other industrial solvents from 1953 to 1985.

**Methods:**

A cohort mortality study was conducted of Marines/Navy personnel who, between 1975 and 1985, began service and were stationed at Camp Lejeune (*N* = 159,128) or MCB Camp Pendleton, California (*N* = 168,406), and civilian workers employed at Camp Lejeune (*N* = 7,332) or Camp Pendleton (*N* = 6,677) between October 1972 and December 1985. Camp Pendleton’s drinking water was not contaminated with industrial solvents. Mortality follow-up was between 1979 and 2018. Proportional hazards regression was used to calculate adjusted hazard ratios (aHRs) comparing mortality rates between Camp Lejeune and Camp Pendleton cohorts. The ratio of upper and lower 95% confidence interval (CI) limits, or CIR, was used to evaluate the precision of aHRs. The study focused on underlying causes of death with aHRs ≥ 1.20 and CIRs ≤ 3.

**Results:**

Deaths among Camp Lejeune and Camp Pendleton Marines/Navy personnel totaled 19,250 and 21,134, respectively. Deaths among Camp Lejeune and Camp Pendleton civilian workers totaled 3,055 and 3,280, respectively. Compared to Camp Pendleton Marines/Navy personnel, Camp Lejeune had aHRs ≥ 1.20 with CIRs ≤ 3 for cancers of the kidney (aHR = 1.21, 95% CI: 0.95, 1.54), esophagus (aHR = 1.24, 95% CI: 1.00, 1.54) and female breast (aHR = 1.20, 95% CI: 0.73, 1.98). Causes of death with aHRs ≥ 1.20 and CIR > 3, included Parkinson disease, myelodysplastic syndrome and cancers of the testes, cervix and ovary. Compared to Camp Pendleton civilian workers, Camp Lejeune had aHRs ≥ 1.20 with CIRs ≤ 3 for chronic kidney disease (aHR = 1.88, 95% CI: 1.13, 3.11) and Parkinson disease (aHR = 1.21, 95% CI: 0.72, 2.04). Female breast cancer had an aHR of 1.19 (95% CI: 0.76, 1.88), and aHRs ≥ 1.20 with CIRs > 3 were observed for kidney and pharyngeal cancers, melanoma, Hodgkin lymphoma, and chronic myeloid leukemia. Quantitative bias analyses indicated that confounding due to smoking and alcohol consumption would not appreciably impact the findings.

**Conclusion:**

Marines/Navy personnel and civilian workers likely exposed to contaminated drinking water at Camp Lejeune had increased hazard ratios for several causes of death compared to Camp Pendleton.

**Supplementary Information:**

The online version contains supplementary material available at 10.1186/s12940-024-01099-7.

## Background

Industrial organic solvents were detected in finished drinking water samples between 1980 and 1985 from two of eight treatment plants serving United States Marine Corps (USMC) Base Camp Lejeune, North Carolina. Each treatment plant served a different area of the base. The Tarawa Terrace (TT) treatment plant began operating in 1952, served family housing, and was contaminated by an off-base dry cleaner. The maximum concentration of tetrachloroethylene (PCE) in the TT distribution system measured 215 micrograms per liter (µg/L) [1].

The Hadnot Point (HP) treatment plant began operation in 1942, served family housing, most workplaces and barracks, field training areas (via mobile “water buffaloes”) and eating establishments, and was contaminated by on-base sources – leaking underground storage tanks, industrial area spills, and waste disposal sites. Maximum concentrations of trichloroethylene (TCE) and PCE in the HP distribution system measured 1,400 µg/L and 100 µg/L, respectively. Also detected in the distribution system were benzene from fuel spills and leaks, and vinyl chloride from degradation of PCE and TCE [2].

No drinking water sampling for volatile organic compounds occurred before 1980. The Agency for Toxic Substances and Disease Registry (ATSDR) conducted historical reconstruction modeling to estimate monthly average contaminant levels in the TT and HP distribution systems and found that contamination started by the mid-1950s [1, 2]. Heavily contaminated supply wells were shut down by February 1985, although benzene was detected in the HP distribution system in late 1985. Contaminant concentrations were minimal after 1985. In each system, water from supply wells was mixed at the treatment plant prior to distribution; and contamination levels varied depending on the wells in use, their levels of contamination and pumpage rates [1, 2].

In the TT distribution system, estimated monthly average concentrations of PCE between January 1975 and February 1985 ranged from 0 to 158 µg/L with a median of 85 µg/L [1]. In the HP distribution system, estimated monthly average concentrations of TCE, PCE and vinyl chloride during this period ranged from 0 to 783 µg/L, 0 to 39 µg/L, and 0 to 67 µg/L, respectively, with medians of 366 µg/L, 15 µg/L and 22 µg/L, respectively [2].

A Marine in training is estimated to consume 6 L/day of drinking water three times per week, and 3 L/day four times per week [3]. Marines/Navy personnel not in training and civilian workers are estimated to consume 3 L/day [3]. Current U.S. maximum contamination levels in drinking water are 5 µg/L for TCE, PCE and benzene, and 2 µg/L for vinyl chloride [4]. TCE, benzene and vinyl chloride are known human carcinogens and PCE is “probably carcinogenic to humans [5–7].

ATSDR previously conducted two cohort mortality studies comparing Marines/Navy personnel and civilian workers stationed or employed at Camp Lejeune from 1975 to 1985 and 1973 to 1985, respectively, with similar cohorts over the same periods stationed or employed at USMC Base Camp Pendleton, California [8, 9]. The follow-up period for both studies was 1979–2008. Both studies found increased risks from cancers of the kidney, rectum, lung, prostate, leukemias, and multiple myeloma. The Marines/Navy personnel had increased risks from cancers of the esophagus, liver, and cervix, Hodgkin lymphoma and multiple sclerosis [8]. The civilian workers had increased risks of oral cancers and Parkinson disease [9].

The purpose of the current study was to determine if being stationed or employed at Camp Lejeune between 1975 and 1985 (Marines/Navy personnel) or between October 1972 and December 1985 (civilian workers), increased the risk of specific causes of death between 1979 and 2018 compared to being stationed or employed at Camp Pendleton. Samples of Camp Pendleton’s drinking water taken between 1989 and 2005 did not detect industrial organic solvents above their MCLs [10].

## Methods

### Study population

The Defense Manpower Data Center (DMDC) quarterly personnel data were obtained for Marines/Navy personnel and civilian workers stationed or employed at Camp Lejeune or Camp Pendleton any quarter between 1975 and 1985 and between October 1972 and December 1985, respectively. DMDC started data collection for civilian workers in October 1972. DMDC data for Marines/Navy personnel did not include military unit codes, necessary to determine base locations, until 1975.

The study included a cohort of 7,332 workers employed at Camp Lejeune and a comparison cohort of 6,677 workers employed at Camp Pendleton, who were known to be alive as of January 1, 1979. The DMDC information included base location of employment, social security number, name, birth date, sex, race, education and occupation code. Based on DMDC data, average employment duration at Camp Lejeune between October 1972 and December 1985 was 64 months.

The full cohort of Marines/Navy personnel included 217,988 at Camp Lejeune and 232,026 at Camp Pendleton, who were known to be alive as of January 1, 1979. DMDC data included social security number, name, birth date, sex, race, rank, education, date active duty started, and military unit code. Based on DMDC data, the average duration that Marines/Navy personnel were stationed at Camp Lejeune between 1975 and 1985 was about 18 months.

Base locations before 1975 could not be determined using DMDC data. Some of the Marines/Navy personnel who began active duty before 1975 could have been stationed at Camp Pendleton between 1975 and 1985, and considered unexposed in the study, but stationed at Camp Lejeune before 1975 and actually exposed. Therefore, a subgroup of the full cohort was identified consisting of Marines/Navy personnel who began active duty between 1975 and 1985. This subgroup consisted of 154,821 at Camp Lejeune and 163,484 at Camp Pendleton and was the focus of the analyses of the Marines/Navy personnel.

Camp Pendleton Marines/Navy personnel and civilian workers were selected as reference cohorts because they were unexposed to drinking water contaminated with industrial solvents [10] and were similar to the Camp Lejeune cohorts on demographic and socioeconomic factors, pre-enlistment screening and fitness requirements, training activities, and types of military and civilian employee occupations. Therefore, biases due to the healthy veteran (or healthy soldier) effect [11, 12] or the healthy worker effect [13], or from unmeasured confounders, should be minimal.

### Vital status ascertainment

To obtain vital status, personal identifiers from the DMDC data were linked to data from a locator firm and the Social Security Administration (SSA) Data for Epidemiological Researchers. SSA matched 99% of the records. Data on individuals who died or had unknown vital status were submitted to the National Death Index (NDI) to obtain International Classification of Diseases (ICD), Ninth and Tenth codes for underlying and contributing causes of death and date of death. About 1% had unknown vital status after the NDI linkage and were lost to follow-up, but contributed person-years until the last date they were known to be alive (Table 1).

**Table 1:** Demographic information for the Marines/Navy personnel subgroup and the civilian workers at risk during the follow-up period (1979–2018)

Base	Camp Lejeune *N* (%)		Camp Pendleton *N* (%) (ref)	
Cohort	Marines/Navy	Civilian workers	Marines/Navy	Civilian workers
Total	159,128 (48.6)	7,332 (52.3%)	168,406 (51.4)	6,677 (47.7%)
Sex				
Male	151,026 (94.9)	3,708 (50.6%)	162,473 (96.5)	3,646 (54.6%)
Female	8,102 (5.1)	3,624 (49.4%)	5,933 (3.5)	3,031 (45.4%)
Race				
White	116,501 (73.2)	5,539 (75.5%)	131,011 (77.8)	5,199 (77.9%)
Black/African American	38,365 (24.1)	1,409 (19.2%)	28,657 (17.0)	498 (7.5%)
Other race	4,262 (2.7)	384 (5.2%)	8,738 (5.2)	980 (14.7%)
Rank				
E1 – E4	130,312 (81.9)	-	137,281 (81.5)	-
E5 – E9	23,049 (14.5)	-	23,434 (13.9)	-
WO or CO	5,767 (3.6)	-	7,691 (4.6)	-
Occupation				
Blue collar	-	2,819 (38.4%)	-	2,798 (41.9%)
White collar	-	4,513 (61.6%)	-	3,879 (58.1%)
Education				
High school graduate	133,140 (83.7)	1,038 (14.2%)	132,871 (78.9)	679 (10.2%)
< High school	19,951 (12.5)	5,206 (71.0%)	27,362 (16.2)	5,539 (83.0%)
College graduate	6,037 (3.8)	1,088 (14.8%)	8,173 (4.9)	459 (6.9%)
Age, start of follow-up				
Mean (years)	20.2	39.1	20.5	41.2
Median (years)	20.0	36.0	20.0	41.0
Age, end of follow-up^*^				
Mean (years)	56.4	71.3	56.6	72.4
Median (years)	57.0	70.0	58.0	72.0
% Age > 55 years	105,054 (66.0%)	6,966 (95.0%)	113,881 (67.6%)	6,363 (95.3%)
% Age > 65 years	2,702 (1.7%)	5,185 (70.7%)	3,367 (2.0%)	4,760 (71.3%)
% Age > 70 years	287 (0.2%)	3,574 (48.7%)	273 (0.2%)	3,586 (53.7%)
Deaths^¥^				
Number	19,250	3,055	21,134	3,280
% of cohort	12.1%	41.7%	12.5%	49.1%
Length of follow-up (years)				
Mean	36.2	31.5	36.1	31.0
Median	38.0	36.0	38.0	35.0
Total person-years	5,760,931	231,496	6,078,598	203,469
Total lost to follow-up	1,072 (0.7%)	111 (1.5%)	1,231 (0.7%)	84 (1.3%)
Quarters in the DMDC data^€^				
Mean	7.7	18.9	7.2	17.3
Median	7.0	12.0	6.0	11.0
IQR	8 (3–11)	30 (3–33)	8 (3–11)	23 (4–27)

E1 – E4: private to corporal; E5 – E9: sergeant to sergeant major; WO: warrant officer; CO: commissioned officer

^*^ Age at end of follow-up (12/31/2018 or date of death if earlier than 12/31/2018)

^¥^ Deaths occurring 1/1/1979–12/31/2018

^€^ Number of quarters stationed at either Camp Lejeune or Camp Pendleton during 1975–1985 for the Marines/Navy subgroup cohort and during 10/72–12/85 for civilian workers cohort

### Data analysis

Marines/Navy personnel and civilian employees were analyzed separately. Follow-up began on January 1, 1979, or the start of employment or military service at Camp Lejeune or Camp Pendleton, whichever was later, and continued until December 31, 2018, if the individual was known to be alive, or to date of death. The main analyses compared Camp Lejeune and Camp Pendleton civilian workers and the Marines/Navy subgroup on underlying causes of death. Secondary analyses evaluated: (1) the full cohort of Marines/Navy personnel, (2) contributing causes of death, and (3) duration stationed or employed at Camp Lejeune as a proxy for cumulative drinking water exposure.

Descriptive analyses included standardized mortality ratios (SMRs) and Poisson regression risk ratios (RRs). SMRs used age-, sex-, race- and calendar period-specific U.S. mortality rates for underlying causes of death using the life table analysis system [14]. Poisson regressions compared sex-, race-, and five-year age-specific underlying causes of death for Camp Lejeune versus Camp Pendleton. Once an individual was stationed or employed at Camp Lejeune, all subsequent person-years were assigned to Camp Lejeune.

Hazard ratios (HRs) and 95% confidence intervals (CIs) were estimated using Cox extended proportional hazards regression [15], with age as the time variable and base location as time-varying. Adjusted models included sex, race, education level, and rank (Marines/Navy personnel) or blue-collar work (civilian workers). Base location was not lagged because > 75% of deaths occurred > 10 years after contamination ended at Camp Lejeune. Schoenfeld residuals were used to check the proportional hazards assumption [15].

Because information on smoking and alcohol consumption was unavailable, negative control diseases [16] were used to estimate prevalence differences between Camp Lejeune and Camp Pendleton. The negative controls for smoking were chronic obstructive pulmonary disease (COPD) and cardiovascular disease mortality. The negative controls for alcohol consumption were alcoholism, alcoholic liver disease, and chronic liver disease mortality. Cancers of the oral cavity, larynx, lung, esophagus, colon, liver, bladder, and female breast were not negative controls for smoking or alcohol consumption because they have been linked to occupational exposures to TCE, PCE, vinyl chloride and/or benzene [17–27].

Quantitative bias analyses (QBA) using Excel spreadsheets were used to estimate possible impacts of confounding due to smoking and alcohol consumption and bias due to non-differential exposure misclassification [28]. Exposure misclassification bias was assumed non-differential and independent because base locations were assigned prior to mortality data collection. The QBA parameters for confounding were the prevalences of the confounding factor in the Camp Lejeune and Camp Pendleton cohorts, and the risk ratio associating the confounder with the cause of death under evaluation. The QBA parameters for exposure misclassification were the sensitivity of the exposure classification, i.e., the probability that the truly exposed were correctly classified as exposed (i.e., assigned to Camp Lejeune) and the specificity of the exposure classification, i.e., the probability that the truly unexposed were correctly classified as unexposed (i.e., assigned to Camp Pendleton). The QBA provided estimates of what the observed adjusted HR (aHR) would have been if the bias were absent. More detail on the methods and results of the QBA are included in the supplemental file.

Statistical significance testing was not used to interpret findings, following the recommendations of a wide range of researchers [29–33]. Instead, the interpretation of findings emphasized: (1) the estimate of the magnitude of the association, i.e., the aHR, (2) the precision of the estimate, using the ratio of the upper to lower limits of the 95% confidence interval (CIR) [34, 35], (3) supporting information from other studies, and (4) the results of the QBA. For rare causes of death (e.g., male breast cancer), interpretation considered findings from the full cohort of Marines/Navy personnel and from analyses of contributing causes.

In this study, aHRs ≥ 1.20 with CIRs ≤ 3 were emphasized. The decision to emphasize aHRs ≥ 1.20 was based on the results of published meta-analyses of studies of TCE exposed workers [17]. Meta-analyses of TCE exposure and kidney cancer, NHL and liver cancer obtained summary RRs between 1.23 and 1.32 [36–38]. An appropriate CIR level for precision has not been specified in the literature. We considered CIRs ≤ 3 to indicate reasonable precision of the aHRs. In this study, an aHR was likely to have a CIR ≤ 3 if both the exposed group and unexposed group had > 25 cases of the cause of death. Although aHRs ≥ 1.20 with CIRs ≤ 3 are emphasized, aHRs < 1.20, or aHRs ≥ 1.20 with CIRs > 3, may also be of importance.

Analyses used SAS 9.4 and STATA 16. This study was approved by the Centers for Disease Control and Prevention Institutional Review Board.

## Results

Table 1 provides demographics for the Marines/Navy personnel subgroup and civilian workers. Demographics and all results for the full cohort of Marines/Navy personnel are in Tables S1-S4. The Camp Lejeune and Camp Pendleton Marines/Navy personnel had similar demographics and were mostly male, white, and ranged in rank from private to corporal. By the end of follow-up, 2% were aged > 65 years and 12% had died. Civilian workers were mostly white with white collar jobs, and > 45% were women. Higher percentages of Camp Lejeune workers were African American and graduated from college compared to Camp Pendleton. By the end of follow-up, > 70% were aged > 65 years and > 40% had died.

SMRs for the Marines/Navy personnel subgroup and civilian workers were < 1.00 for most causes of death (Tables 2 and 3), consistent with a healthy veteran effect [11, 12] and healthy worker effect [13]. Factors producing a healthy veteran effect include the initial recruitment physical screening, fitness standards during military service, and access to health care during and after service. The effect was likely strong in the Marines/Navy personnel subgroup because most were aged < 65 years at the end of follow-up.

**Table 2:** Standardized mortality ratios (SMR), Poisson regression risk ratios, and 95% confidence intervals (CI) for the Camp Lejeune and Camp Pendleton Marines/Navy personnel subgroup: underlying cause of death

Cause of Death	Camp Lejeune (CL)	Camp Pendleton (CP)	Risk Ratio (95% CI)
	Observed SMR (95% CI)	Observed SMR (95% CI)	**CL vs. CP**
All Causes	19,250 0.90 (0.89, 0.91)	21,134 0.93 (0.92, 0.94)	0.99 (0.97, 1.01)
All Cancer Malignancies	3,689 0.92 (0.89, 0.95)	3,760 0.87 (0.84, 0.90)	1.07 (1.02, 1.12)
Oral Cavity and Pharynx	106 0.85 (0.70, 1.03)	124 0.92 (0.77, 1.10)	0.95 (0.73, 1.23)
Esophagus	171 1.01 (0.87, 1.18)	154 0.83 (0.70, 0.97)	1.25 (1.00, 1.55)
Stomach	89 0.78 (0.62, 0.95)	100 0.83 (0.68, 1.01)	0.93 (0.70, 1.24)
Colon	266 0.87 (0.77, 0.98)	264 0.81 (0.71, 0.91)	1.07 (0.91, 1.27)
Rectum	83 0.79 (0.63, 0.98)	94 0.83 (0.67, 1.02)	0.93 (0.69, 1.26)
Liver/Biliary System	268 0.84 (0.75, 0.95)	289 0.85 (0.76, 0.96)	1.01 (0.86, 1.20)
Pancreas	265 1.06 (0.94, 1.19)	250 0.92 (0.81, 1.04)	1.15 (0.97, 1.37)
Larynx	36 0.76 (0.53, 1.06)	39 0.77 (0.55, 1.05)	1.04 (0.66, 1.64)
Lung/Trachea/Bronchus	982 0.97 (0.91, 1.03)	915 0.83 (0.78, 0.89)	1.19 (1.08, 1.30)
Connective Tissue	53 1.03 (0.77, 1.34)	50 0.90 (0.67, 1.19)	1.17 (0.80, 1.73)
Melanoma	105 0.98 (0.80, 1.18)	109 0.89 (0.73, 1.07)	1.09 (0.84, 1.43)
Breast Cancer - Female	41 0.92 (0.66, 1.25)	25 0.72 (0.47, 1.07)	1.23 (0.75, 2.03)
Breast Cancer - Male	4 0.69 (0.19, 1.75)	11 1.76 (0.88, 3.15)	0.39 (0.12, 1.22)
Cervix	9 1.07 (0.49, 2.03)	5 0.79 (0.26, 1.85)	1.21 (0.39, 3.72)
Uterus	5 0.91 (0.30, 2.12)	7 1.59 (0.64, 3.28)	0.59 (0.19, 1.88)
Ovary	8 0.72 (0.31, 1.42)	6 0.67 (0.24, 1.45)	1.19 (0.41, 3.44)
Prostate	95 1.01 (0.81, 1.23)	108 1.06 (0.87, 1.28)	0.94 (0.71, 1.24)
Testis	18 0.72 (0.42, 1.15)	12 0.45 (0.23, 0.79)	1.72 (0.83, 3.58)
Kidney and Renal Pelvis	139 1.11 (0.93, 1.31)	126 0.91 (0.76, 1.09)	1.21 (0.95, 1.54)
Urinary Bladder	61 0.97 (0.74, 1.24)	66 0.94 (0.73, 1.20)	1.02 (0.72, 1.45)
Brain and CNS	178 0.91 (0.78, 1.06)	217 1.00 (0.87, 1.15)	0.90 (0.74, 1.10)
Thyroid	8 0.78 (0.34, 1.55)	12 1.07 (0.55, 1.88)	0.72 (0.30, 1.77)
Hematopoietic Cancers	354 0.83 (0.75, 0.92)	380 0.83 (0.74, 0.91)	1.00 (0.87, 1.16)
Hodgkin Lymphoma	30 0.93 (0.63, 1.32)	32 0.91 (0.62, 1.29)	1.00 (0.61, 1.65)
NHL	122 0.73 (0.60, 0.87)	151 0.83 (0.70, 0.97)	0.87 (0.68, 1.10)
Multiple Myeloma	62 0.99 (0.77, 1.26)	61 0.92 (0.70, 1.18)	1.08 (0.76, 1.54)
Leukemias	142 0.87 (0.73, 1.02)	136 0.77 (0.65, 0.91)	1.13 (0.89, 1.43)
Diabetes	400 0.71 (0.64, 0.78)	452 0.75 (0.69, 0.83)	0.94 (0.82, 1.08)
Alcoholism	242 0.93 (0.81, 1.05)	302 1.07 (0.95, 1.20)	0.88 (0.74, 1.04)
Multiple Sclerosis	29 0.78 (0.52, 1.12)	27 0.68 (0.45, 0.99)	1.12 (0.66, 1.89)
Parkinson Disease	15 1.47 (0.73, 2.21)	8 0.69 (0.21, 1.17)	2.00 (0.85, 4.73)
ALS	64 1.12 (0.85, 1.39)	67 1.05 (0.80, 1.30)	1.05 (0.75, 1.48)
Cardiovascular Disease	4,316 0.90 (0.87, 0.93)	4,650 0.91 (0.88, 0.94)	1.00 (0.96, 1.04)
COPD	312 0.96 (0.86, 1.07)	320 0.89 (0.79, 0.99)	1.10 (0.94, 1.28)
Chronic Liver Disease	614 0.79 (0.73, 0.86)	775 0.91 (0.84, 0.97)	0.88 (0.79, 0.97)
Chronic Kidney Disease	133 0.62 (0.52, 0.74)	139 0.64 (0.54, 0.75)	0.99 (0.78, 1.25)
Suicide	1,664 1.21 (1.16, 1.27)	2,002 1.32 (1.27, 1.38)	0.92 (0.86, 0.98)

CNS: central nervous system; NHL: non-Hodgkin lymphoma; ALS: amyotrophic lateral sclerosis; COPD: chronic obstructive pulmonary disease

SMRs were calculated using the age-, sex-, race- and calendar period-specific U.S. mortality rates for underlying causes of death

Risk ratios were adjusted for sex, race, and five-year age groups

**Table 3:** Standardized mortality ratios (SMR), Poisson regression risk ratios, and 95% confidence intervals (CI) for Camp Lejeune and Camp Pendleton civilian employees: underlying cause of death

Cause of Death	CL	CP	Risk Ratio (95% CI)
	Observed SMR (95% CI)	Observed SMR (95% CI)	**CL vs. CP**
All causes	3,055 0.89 (0.85, 0.92)	3,280 0.90 (0.87, 0.94)	0.96 (0.91, 1.01)
All cancers	882 0.93 (0.87, 0.99)	890 0.93 (0.87, 0.99)	1.01 (0.92, 1.12)
All malignant cancers	859 0.91 (0.85, 0.98)	874 0.91 (0.85, 0.98)	1.01 (0.91, 1.11)
Oral cavity and pharynx	10 0.62 (0.31, 1.10)	10 0.62 (0.31, 1.10)	1.06 (0.43, 2.61)
Pharynx	8 0.95 (0.41, 1.88)	4 0.48 (0.13, 1.24)	2.14 (0.61, 7.46)
Esophagus	13 0.51 (0.27, 0.88)	24 0.96 (0.61, 1.43)	0.63 (0.31, 1.27)
Stomach	21 0.85 (0.53, 1.30)	21 0.87 (0.54, 1.33)	1.00 (0.53, 1.90)
Colon	46 0.61 (0.45, 0.82)	55 0.70 (0.53, 0.92)	0.85 (0.56, 1.29)
Rectum	13 0.87 (0.46, 1.48)	14 0.92 (0.50, 1.54)	0.95 (0.43, 2.08)
Liver/Biliary system	20 0.62 (0.38, 0.96)	29 0.92 (0.62, 1.32)	0.73 (0.40, 1.32)
Pancreas	41 0.78 (0.56, 1.06)	63 1.20 (0.92, 1.53)	0.72 (0.48, 1.08)
Larynx	8 0.94 (0.41, 1.86)	5 0.59 (0.19, 1.38)	1.36 (0.42, 4.40)
Lung	310 1.08 (0.96, 1.20)	281 0.96 (0.85, 1.08)	1.15 (0.97, 1.36)
Kidney and renal pelvis	24 1.16 (0.74, 1.73)	15 0.70 (0.39, 1.16)	1.49 (0.76, 2.92)
Urinary bladder	18 0.85 (0.50, 1.34)	25 1.06 (0.69, 1.57)	0.65 (0.34, 1.24)
Melanoma	12 1.03 (0.53, 1.80)	5 0.41 (0.13, 0.97)	2.59 (0.89, 7.56)
Connective tissue	5 0.90 (0.29, 2.11)	6 1.10 (0.40, 2.40)	0.65 (0.19, 2.22)
Brain and CNS	17 0.87 (0.50, 1.39)	28 1.44 (0.96, 2.09)	0.66 (0.36, 1.23)
Thyroid	1	0	
Hematopoietic cancers	84 1.00 (0.80, 1.24)	90 1.02 (0.82, 1.25)	1.00 (0.73, 1.36)
Hodgkin lymphoma	3 1.47 (0.30, 4.29)	2 0.98 (0.12, 3.55)	1.81 (0.30, 11.0)
NHL	35 1.12 (0.78, 1.56)	38 1.13 (0.80, 1.55)	0.98 (0.61, 1.58)
Multiple myeloma	15 0.79 (0.44, 1.30)	13 0.68 (0.36, 1.16)	0.99 (0.45, 2.16)
Leukemias	31 0.98 (0.67, 1.39)	37 1.11 (0.78, 1.53)	1.00 (0.61, 1.64)
Breast cancer - Female	48 0.84 (0.62, 1.12)	33 0.63 (0.44, 0.89)	1.21 (0.77, 1.89)
Breast cancer - Male	0	0	
Cervix	1	3 0.55 (0.11, 1.60)	
Uterus	10 1.01 (0.49, 1.87)	9 1.00 (0.46, 1.90)	0.97 (0.39, 2.43)
Ovary	14 0.75 (0.41, 1.26)	22 1.28 (0.80, 1.94)	0.57 (0.29, 1.13)
Prostate	71 0.99 (0.78, 1.25)	59 0.80 (0.61, 1.04)	1.01 (0.69, 1.50)
Testis	0	0	
Diabetes	94 0.90 (0.72, 1.10)	103 0.98 (0.80, 1.19)	0.78 (0.58, 1.05)
Alcoholism	7 0.56 (0.23, 1.16)	10 0.87 (0.42, 1.61)	0.63 (0.23, 1.74)
Multiple sclerosis	3 0.63 (0.13, 1.85)	3 0.73 (0.15, 2.12)	0.70 (0.14, 3.52)
Parkinson disease	30 1.34 (0.86, 1.82)	31 1.19 (0.77, 1.60)	1.15 (0.68, 1.93)
ALS	5 0.57 (0.07, 1.07)	10 1.12 (0.43, 1.82)	0.44 (0.14, 1.32)
Anemias	7 1.20 (0.48, 2.47)	3 0.48 (0.10, 1.41)	1.61 (0.39, 6.61)
Heart/Circulatory disease	1105 0.88 (0.83, 0.93)	1271 0.93 (0.88, 0.98)	0.92 (0.85, 1.00)
COPD	171 0.99 (0.85, 1.15)	213 1.13 (0.99, 1.30)	0.91 (0.73, 1.11)
Chronic liver disease	36 0.70 (0.49, 0.97)	51 1.04 (0.77, 1.36)	0.71 (0.46, 1.10)
Chronic kidney disease	49 0.84 (0.62, 1.11)	26 0.43 (0.28, 0.63)	1.77 (1.07, 2.93)
Suicide	29 0.84 (0.56, 1.21)	45 1.36 (0.99, 1.82)	0.68 (0.42, 1.10)

CL: Camp Lejeune; CP: Camp Pendleton; SMR: Standardized mortality ratio; CI: Confidence interval; CNS: Central nervous system cancers; NHL: Non-Hodgkin lymphoma; ALS: Amyotrophic Lateral Sclerosis; COPD: Chronic obstructive pulmonary disease

SMRs were calculated using the age-, sex-, race- and calendar period-specific U.S. mortality rates for underlying causes of death

Risk ratios were adjusted for sex, race, and five-year age groups

The Marines/Navy personnel subgroup analysis obtained Poisson regression RRs ≥ 1.20 with CIRs ≤ 3 for cancers of the esophagus, kidney and female breast, and RRs ≥ 1.20 with CIRs > 3 for cervical and testicular cancers and Parkinson disease (Table 2). The civilian workers analysis obtained Poisson regression RRs ≥ 1.20 with CIRs ≤ 3 for female breast cancer and chronic kidney disease, and RRs ≥ 1.20 and CIRs > 3 for cancers of the kidney, pharynx, and larynx, and melanoma, Hodgkin lymphoma, and anemias (Table 3).

Cox proportional hazards regression analysis of the Marines/Navy personnel subgroup and underlying causes of death obtained aHRs ≥ 1.20 with CIRs ≤ 3 for cancers of the kidney, esophagus, and female breast (Table 4). Parkinson disease, myelodysplastic syndrome, and cancers of the testes, cervix, and ovary had aHRs ≥ 1.20 with CIRs > 3. In the analyses of the full cohort of Marines/Navy personnel, additional underlying causes of death with aHRs ≥ 1.20 and CIRs ≤ 3 included acute myeloid leukemia (AML), Hodgkin lymphoma, multiple sclerosis, and acute kidney disease (Table S3).

**Table 4:** Hazard ratios (HR) and 95% confidence intervals (CI) for the Marines/Navy personnel subgroup analysis of base location at Camp Lejeune (CL) vs. Camp Pendleton (CP); underlying cause of death

Outcome	Total	Camp Lejeune #	Unadjusted HR (95% CI)	Adjusted HR (95% CI)	Camp Pendleton #
All causes	40,384	19,250	0.98(0.96, 1.00)	0.99(0.97, 1.01)	21,134
All cancer malignancies	7,449	3,689	1.06(1.01, 1.11)	1.06(1.02, 1.11)	3,760
Oral cancers	230	106	0.92(0.71, 1.19)	0.95(0.73, 1.24)	124
Pharyngeal cancer	121	56	0.93(0.65, 1.33)	0.94(0.66, 1.35)	65
Esophageal cancer	325	171	1.20(0.97, 1.49)	1.24 (1.00, 1.54)	154
Stomach cancers	189	89	0.96(0.72, 1.28)	0.92(0.69, 1.23)	100
Colorectal cancers	707	349	1.05(0.91, 1.22)	1.03(0.88, 1.19)	358
Colon cancer	530	266	1.09(0.92, 1.29)	1.05(0.89, 1.25)	264
Rectal cancer	177	83	0.95(0.71, 1.27)	0.94(0.70, 1.27)	94
Liver cancer	557	268	1.00(0.85, 1.19)	1.06(0.89, 1.25)	289
Pancreatic cancer	515	265	1.14(0.96, 1.36)	1.14(0.96, 1.36)	250
Laryngeal cancer	75	36	1.00(0.64, 1.57)	1.01(0.64, 1.58)	39
Lung cancer	1,897	982	1.16(1.06, 1.27)	1.18(1.07, 1.29)	915
Bone cancers	40	16	0.71(0.38, 1.34)	0.74(0.39, 1.39)	24
Soft tissue cancers	103	53	1.14(0.77, 1.67)	1.12(0.76, 1.65)	50
Melanoma	214	105	1.03(0.79, 1.35)	1.07(0.82, 1.40)	109
Female Breast cancer	66	41	1.27(0.77, 2.08)	1.20 (0.73, 1.98)	25
Male Breast cancer	15	4	0.39(0.13, 1.24)	0.36 (0.12, 1.14)	11
Cervical cancer	14	9	1.19(0.39, 3.65)	1.25 (0.40, 3.85)	5
Uterine cancer	12	5	0.62(0.20, 1.97)	0.62 (0.20, 1.98)	7
Ovarian cancer	14	8	1.12(0.39, 3.24)	1.23 (0.42, 3.59)	6
Prostate cancer	203	95	0.97(0.74, 1.28)	0.93 (0.71, 1.23)	108
Testicular cancer	30	18	1.62(0.78, 3.36)	1.76 (0.85, 3.67)	12
Bladder cancer	127	61	1.00(0.71, 1.42)	1.02(0.72, 1.45)	66
Kidney cancer	265	139	1.19(0.93, 1.51)	1.21(0.95, 1.54)	126
Brain and CNS cancers	395	178	0.88(0.72, 1.07)	0.89(0.73, 1.09)	217
Thyroid cancer	20	8	0.71(0.29, 1.74)	0.71(0.29, 1.74)	12
Hematopoietic cancers	734	354	1.00(0.86, 1.16)	0.99(0.86, 1.14)	380
Hodgkin lymphoma	62	30	1.00(0.61, 1.64)	0.98(0.59, 1.61)	32
Non-Hodgkin lymphoma	273	122	0.87(0.68, 1.10)	0.87(0.68, 1.10)	151
Multiple myeloma	123	62	1.10(0.77, 1.56)	1.07(0.75, 1.53)	61
Leukemias	278	142	1.12(0.89, 1.42)	1.10(0.87, 1.40)	136
ALL	45	20	0.85(0.47, 1.53)	0.85(0.47, 1.53)	25
CLL	15	7	0.94(0.34, 2.59)	0.89(0.32, 2.48)	8
AML	122	62	1.12(0.78, 1.59)	1.11(0.78, 1.59)	60
CML	28	11	0.69(0.32, 1.47)	0.65(0.30, 1.40)	17
MDS	17	11	1.96(0.73, 5.30)	2.26(0.83, 6.17)	6
Lymphoid cancers	63	27	0.82(0.50, 1.35)	0.80(0.49, 1.33)	36
Myeloid cancers	157	79	1.12(0.82, 1.53)	1.10(0.80, 1.50)	78
Diabetes	852	400	0.96(0.84, 1.10)	0.96(0.83, 1.09)	452
Anemias	25	12	1.00(0.46, 2.19)	0.89(0.41, 1.97)	13
Cardiovascular disease	8,966	4,316	1.00(0.96, 1.04)	0.99(0.95, 1.03)	4,650
Heart disease	7,331	3,512	0.99(0.95, 1.04)	0.99(0.94, 1.03)	3,819
Stroke	861	432	1.08(0.95, 1.24)	1.05(0.92, 1.20)	429
Circulatory diseases	773	371	0.99(0.86, 1.14)	0.95(0.82, 1.09)	402
COPD	632	312	1.06(0.91, 1.24)	1.08(0.93, 1.27)	320
Chronic Liver disease	1,389	614	0.85(0.77, 0.95)	0.93(0.83, 1.03)	775
Cirrhosis	1,560	693	0.86(0.78, 0.95)	0.93(0.84, 1.03)	867
Alcoholic Liver disease	901	381	0.79(0.69, 0.90)	0.86(0.76, 0.99)	520
Nonalcoholic Liver disease	488	233	0.98(0.82, 1.17)	1.06(0.89, 1.27)	255
Acute Kidney disease	42	18	0.81(0.44, 1.50)	0.81(0.44, 1.50)	24
Chronic Kidney disease	272	133	1.03(0.82, 1.31)	0.96(0.75, 1.22)	139
Parkinson disease	23	15	2.09(0.89, 4.94)	2.05(0.86, 4.87)	8
ALS	131	64	1.03(0.73, 1.45)	1.04(0.73, 1.46)	67
Multiple sclerosis	56	29	1.18(0.70, 1.99)	1.18(0.70, 2.00)	27
Alcoholism	544	242	0.86(0.72, 1.02)	0.90(0.76, 1.07)	302
Suicide	3,666	1,664	0.88(0.83, 0.94)	0.93(0.87, 0.99)	2,002

CL = 159,128 Males = 151,026 Females = 8,102; CP = 168,406 Males = 162,473 Females = 5,933; Total = 327,534 Males = 313,499 Females = 14,035; COPD: chronic obstructive pulmonary disease; ALS: amyotrophic lateral sclerosis; MDS: myelodysplastic syndrome; CML: chronic myeloid leukemia; AML: acute myeloid leukemia; CLL: chronic lymphocytic leukemia; ALL: acute lymphocytic leukema; CNS: central nervous system

HRs adjusted for sex, race, rank and education level; age was the time variable

Results for contributing causes of death among the Marines/Navy personnel subgroup were similar to results for underlying causes (Table S5). Multiple sclerosis had an aHR ≥ 1.20 and CIRs ≤ 3. In the full cohort of Marines/Navy personnel, an aHR ≥ 1.20 with CIR > 3 was observed for male breast cancer as contributing cause (Table S4).

Cox proportional hazards regression analysis of civilian workers and underlying causes of death obtained aHRs ≥ 1.20 with CIRs ≤ 3 for chronic kidney disease and Parkinson disease (Table 5). Female breast cancer had an aHR of 1.19 with CIR ≤ 3. Cancers of the kidney and pharynx, melanoma, Hodgkin lymphoma, chronic myeloid leukemia (CML), and anemias had aHRs ≥ 1.20 with CIRs > 3. Analysis of contributing causes of death obtained an aHR ≥ 1.20 with CIR ≤ 3 for female breast cancer, and aHRs ≥ 1.20 with CIRs > 3 for cancers of the pharynx and larynx, melanoma, Hodgkin lymphoma, and CML (Table S7).

**Table 5:** Comparison of Camp Lejeune (CL) and Camp Pendleton (CP) civilian workers: underlying cause of death

Outcome	Total	Camp Lejeune #	Unadjusted HR (95% CI)	Adjusted HR (95% CI)	Camp Pendleton #
All causes	6,335	3,055	0.96 (0.91, 1.01)	0.96(0.91, 1.01)	3,280
All cancers	1,772	882	0.99(0.91, 1.09)	1.00(0.91, 1.11)	890
All malignancies	1,733	859	0.98(0.90, 1.08)	1.00(0.91, 1.10)	874
Oral cancers	20	10	1.00(0.42, 2.41)	1.03(0.41, 2.58)	10
Pharynx	12	8	2.02(0.61, 6.71)	2.21(0.63, 7.78)	4
Esophagus	37	13	0.54(0.28, 1.07)	0.63(0.31, 1.28)	24
Stomach	42	21	0.98(0.53, 1.79)	0.92(0.48, 1.76)	21
Colorectal cancers	126	58	0.86(0.61, 1.23)	0.86(0.59, 1.24)	68
Colon	101	46	0.85(0.57, 1.26)	0.85(0.56, 1.28)	55
Rectum and Rectosigmoid junction	27	13	0.93(0.43, 1.97)	0.95(0.43, 2.09)	14
Rectum only	17	8	0.87(0.34, 2.27)	0.84(0.31, 2.27)	9
Liver, biliary, gall bladder	49	20	0.71(0.40, 1.25)	0.77(0.43, 1.39)	29
Liver and bile ducts	38	16	0.75(0.39, 1.43)	0.80(0.41, 1.57)	22
Primary liver	15	6	0.68(0.24, 1.92)	0.87(0.31, 2.48)	9
Pancreas	104	41	0.64(0.43, 0.95)	0.71(0.47, 1.06)	63
Larynx	13	8	1.52(0.50, 4.67)	1.19(0.37, 3.83)	5
Lung	591	310	1.10(0.94, 1.30)	1.13(0.96, 1.34)	281
Urinary bladder	43	18	0.78(0.43, 1.43)	0.65(0.34, 1.26)	25
Kidney	39	24	1.56(0.82, 2.98)	1.44(0.73, 2.84)	15
Brain and CNS	45	17	0.58(0.32, 1.07)	0.61(0.33, 1.13)	28
Connective tissue	11	5	0.81(0.25, 2.65)	0.60(0.17, 2.14)	6
Melanoma	17	12	2.42(0.85, 6.89)	3.03(1.05, 8.76)	5
Hematopoietic cancers	174	84	0.95(0.70, 1.28)	1.00(0.73, 1.36)	90
Lymphoid cancers	17	6	0.57(0.21, 1.53)	0.84(0.30, 2.34)	11
Myeloid cancers	40	19	0.89(0.48, 1.66)	0.98(0.51, 1.88)	21
Hodgkin lymphoma	5	3	1.48(0.25, 8.86)	1.65(0.27, 9.96)	2
Non-Hodgkin lymphoma	73	35	0.97(0.61, 1.53)	0.95(0.59, 1.54)	38
Multiple myeloma	28	15	1.11(0.53, 2.33)	1.02 (0.47, 2.24)	13
Leukemias	68	31	0.85(0.53, 1.37)	1.00(0.61, 1.64)	37
CLL	13	5	0.68(0.22. 2.09)	0.85(0.27, 2.63)	8
AML	31	15	0.93(0.46, 1.88)	0.97(0.47, 2.02)	16
CML	8	4	0.99(0.25, 3.96)	1.26(0.29, 5.48)	4
Female Breast	81	48	1.24 (0.79, 1.93)	1.19 (0.76, 1.88)	33
Uterus	19	10	1.00 (0.41, 2.48)	0.99 (0.39, 2.49)	9
Ovary	36	14	0.58 (0.29, 1.13)	0.60 (0.30, 1.19)	22
Prostate	130	71	1.32 (0.93, 1.86)	1.03 (0.71, 1.51)	59
Diabetes	197	94	0.94(0.71, 1.24)	0.81(0.60, 1.10)	103
Cardiovascular disease	2,377	1,105	0.91(0.84, 0.99)	0.91(0.83, 0.99)	1,272
Anemias	10	7	2.38(0.61, 9.24)	1.91(0.48, 7.65)	3
Chronic liver disease	87	36	0.64(0.42, 0.98)	0.74(0.48, 1.15)	51
Alcoholic liver disease	49	16	0.43(0.24, 0.78)	0.54(0.29, 1.00)	33
Nonalcoholic liver disease	36	19	1.04(0.54, 2.01)	1.11(0.57, 2.17)	17
Alcoholism	17	7	0.63(0.24, 1.65)	0.67(0.24, 1.83)	10
Chronic kidney disease	75	49	2.04(1.27, 3.29)	1.88(1.13, 3.11)	26
COPD	384	171	0.86(0.70, 1.05)	0.91(0.74, 1.12)	213
Multiple sclerosis	6	3	0.97(0.19, 4.80)	0.83(0.16, 4.25)	3
Amyotrophic Lateral Sclerosis	15	5	0.50(0.17, 1.47)	0.44(0.15, 1.34)	10
Parkinson disease	61	30	1.09(0.66, 1.80)	1.21(0.72, 2.04)	31
Suicide	74	29	0.59(0.37, 0.94)	0.72(0.45, 1.16)	45

HR: hazard ratio; CI: confidence interval; CNS: Central nervous system cancers; CLL: Chronic lymphcytic leukemia; AML: Acute myeloid leukemia; CML: Chronic myeloid leukemia; COPD: Chronic obstructive pulmonary disease

Totals: Camp Lejeune = 7,332 Females = 3,624 Males = 3,708; Camp Pendleton = 6,677 Females = 3,031 Males = 3,646

Causes of death that were not evaluated because the number of cases were < 2 for CL and/or CP: Testicular cancer; Male breast cancer; Thyroid cancer; Acute lymphocytic leukemia

HRs adjusted for sex, race, blue collar work (y/n) and education level; age was the time variable

Blue collar work included manual jobs such as maintenance workers, mechanics, construction workers, laundry and dry-cleaning workers, pest control workers, and water treatment plant workers

Results of the duration analyses should be interpreted with caution because monthly contamination levels fluctuated considerably between 1972 and 1985. Duration stationed or employed at Camp Lejeune was categorized into tertiles of the quarterly data after removal of the Camp Pendleton reference group (Tables S6, S8). Monotonic trends with aHRs ≥ 1.20 and CIRs > 3 in each stratum were observed for myelodysplastic syndrome among the Marines/Navy personnel subgroup and kidney cancer among civilian workers.

For the Marines/Navy personnel subgroup, the negative control diseases for smoking had aHRs < 1.00 except for COPD as an underlying cause with an aHR of 1.08 (Table 4, Table S5). Using the HR of 1.08 for COPD, the prevalence difference in smoking between Camp Lejeune and Camp Pendleton would be 6% (Table S9). Assuming a 6% smoking prevalence difference, the HRs for cancers of the kidney, esophagus and lung would decrease by ≤ 3.3%, ≤ 7.3%, and ≤ 9.3%, respectively (Tables S10-S12). Because smoking decreases the risk of Parkinson disease [39], the HR for Parkinson disease would increase by ≤ 6.8% (Table S13).

For civilian workers, COPD as a contributing cause had an aHR of 1.05, with other negative control diseases having aHRs < 1.00 (Table 5, Table S7). Using the HR of 1.05 for COPD, the prevalence difference in smoking between Camp Lejeune and Camp Pendleton workers would be 4% (Table S14). Assuming a 4% smoking prevalence difference, the HRs for cancers of the lung and larynx would decrease by ≤ 7.1%, the HR for pharyngeal cancer would decrease by ≤ 6.3%, and the HRs for kidney cancer and chronic kidney disease would decrease by ≤ 2.8% (Tables S15-S20). For Parkinson disease, the HR would increase by ≤ 4.1% (Table S21).

To estimate the prevalence difference in alcohol consumption between the Camp Lejeune and Camp Pendleton Marines/Navy personnel subgroup, the chronic liver disease HR of 0.93 was used. The prevalence difference would range between 6% and 10% (Table S22). Assuming a prevalence difference of 8%, the HRs for cancers of the esophagus, larynx and female breast would increase by ≤ 8.9%, ≤ 7.0%, and ≤ 3.3%, respectively (Tables S23-S25).

To estimate the prevalence difference in alcohol consumption between Camp Lejeune and Camp Pendleton civilian workers, the chronic liver disease HR of 0.74 was used. The prevalence difference would range between 15% and 25% (Table S26). Assuming a prevalence difference of 15%, the HRs for cancers of the oral cavity, larynx, pharynx, and female breast would increase by ≤ 25.9%, ≤ 17.6%, ≤ 26%, and ≤ 6.7%, respectively (Tables S27-S30).

For the Marines/Navy personnel subgroup, non-differential exposure misclassification bias would increase the HRs for Parkinson disease and cancers of the kidney, esophagus, and lung by ≤ 17.1%, ≤ 5%, ≤ 6.5%, and ≤ 4.2%, respectively (Table S31). For civilian workers, this bias would increase the HRs for cancers of the lung and female breast by < 3.5%, and increase the HRs for kidney cancer, chronic kidney disease and Parkinson disease by ≤ 12.5%, ≤ 13.3%, and ≤ 5%, respectively. The HR for female breast cancer as a contributing cause would increase by ≤ 6% (Table S32).

## Discussion

This cohort mortality study of Marines/Navy personnel and civilian workers at Camp Lejeune and Camp Pendleton included 40 years of follow-up, from 1979 to 2018. Person-years of follow-up totaled over 11 million for the Marines/Navy personnel subgroup and over 400,000 for the civilian workers.

Kidney cancer had HRs ≥ 1.20 with CIR ≤ 3 for the Marines/Navy personnel subgroup and CIR > 3 for civilian workers. HRs ≥ 1.20 were also observed for kidney cancer in the previous mortality studies of Marines/Navy personnel and civilian workers [8, 9]. TCE exposure is a known cause of kidney cancer [5].

Female breast cancer had HRs ≥ 1.20 with CIR ≤ 3 for the Marines/Navy personnel subgroup and as a contributing cause among civilian workers. A meta-analysis of occupational studies and breast cancer obtained pooled odds ratios for benzene and TCE of 1.12 (95% CI: 0.96, 1.31) and 1.19 (95% CI: 0.92, 1.53), respectively [25]. Occupational exposures to PCE and benzene were associated with breast cancer among pre-menopausal [26] and post-menopausal women [27]. In one study, PCE exposed workers had higher mammographic densities [40]. PCE-contaminated drinking water in Cape Cod, MA was associated with increased risk for breast cancer [41].

Male breast cancer as a contributing cause had a HR ≥ 1.20 with CIR > 3 in the analysis of the full cohort of Marines/Navy personnel. A case-control study of male breast cancer incidence found an increased risk among Camp Lejeune Marines compared to Marines from other bases [42]. Occupational TCE exposure has been associated with male breast cancer in three studies [43–45].

HRs ≥ 1.20 were found for myelodysplastic syndrome (CIR > 3) and AML (CIR ≤ 3) among the Marines/Navy personnel subgroup and the full cohort, respectively. The previous mortality study of Marines/Navy personnel did not evaluate these cancers [8]. Benzene exposure has been associated with myelodysplastic syndrome [46] and is a known cause of AML [7].

The Marines/Navy personnel subgroup had HRs ≥ 1.20 and a CIR ≤ 3 for esophageal cancer and CIRs > 3 for cervical, ovarian and testicular cancers. Hodgkin lymphoma had HRs ≥ 1.20 and a CIR ≤ 3 in the full cohort of Marines/Navy personnel and a CIR > 3 civilian workers. The previous Camp Lejeune mortality study of Marines/Navy personnel found HRs ≥ 1.20 for esophageal and cervical cancers and Hodgkin lymphoma but could not evaluate ovarian and testicular cancers [8].

Cancers of the pharynx, larynx (contributing cause), melanoma and CML had HRs ≥ 1.20 and CIRs > 3 among civilian workers. In the previous mortality study of Camp Lejeune civilian workers, an HR ≥ 1.20 was observed for oral cavity cancers, which included pharyngeal cancer [9]. Occupational exposures to PCE and/or TCE among men and women were associated with cancers of the larynx and pharynx [23, 24]. One occupational study found an association between TCE and melanoma (OR = 3.2, 95% CI: 1.0, 9.9) [47]. Associations between occupational benzene exposure and CML were found in a meta-analysis [48] and among benzene workers in China [49].

Parkinson disease had HRs ≥ 1.20 with CIR ≤ 3 among civilian workers and CIR > 3 for the Marines/Navy personnel subgroup. The previous Camp Lejeune mortality studies found an increase in Parkinson disease among civilian workers but could not be evaluated among Marines/Navy personnel [8, 9]. In a recent study, Camp Lejeune Marines/Navy personnel had a 70% increased risk of Parkinson disease diagnoses compared to Camp Pendleton [50]. In animal studies, TCE exposure reproduces key pathological features of Parkinson disease [50, 51].

Multiple sclerosis had HRs ≥ 1.20 with CIRs ≤ 3 for the full cohort of Marines/Navy personnel and as a contributing cause among civilian workers. Multiple sclerosis had an HR ≥ 1.20 in the previous mortality study of Marines/Navy personnel [8]. Occupational organic solvent exposure was associated with multiple sclerosis, but specific solvents were not studied [52].

HRs ≥ 1.20 with CIRs ≤ 3 were observed for chronic kidney disease among civilian workers and acute kidney disease in the full cohort of Marines/Navy personnel. Epidemiological and animal studies support causal associations between TCE and PCE exposures and chronic kidney disease [17].

A study weakness was lack of data on risk factors such as smoking, alcohol consumption, and occupational history before and after active-duty service or employment at Camp Lejeune or Camp Pendleton. However, confounding due to failure to adjust for unmeasured risk factors was likely minor because of the demographic and socio-economic similarity of the Camp Lejeune and Camp Pendleton cohorts.

Prevalence of smoking and “heavy alcohol” consumption among Marines in 1980 was 53.4% and 28.6%, respectively [53], and was encouraged at both bases by the military culture, the stress of service, targeted advertising by the tobacco and alcoholic beverage industry, and the lower cost and tax-free availability of these products on base compared to civilian stores off-base [53, 54]. Quantitative bias analysis of smoking indicated that prevalence differences between Camp Lejeune and Camp Pendleton would range between 4% and 6%. For the strongly smoking-related cancers of the lung and larynx, adjusting for smoking would decrease their HRs by < 10%.

For alcohol consumption, the negative control diseases likely overstated the prevalence differences between the two bases. For alcohol-related causes of death, adjustment for alcohol consumption would increase their HRs. Moreover, for causes of death both smoking-related and alcohol-related, the impacts of adjusting for alcohol consumption and smoking would cancel each other.

Another study weakness was use of base location as a proxy for drinking water exposure. Quantitative bias analyses suggested the impact of this source of non-differential exposure misclassification would be minor in most instances. Disease misclassification bias due to cause of death errors on the death certificate would likely be non-differential and would tend to bias the HRs for the dichotomous comparisons between Camp Lejeune and Camp Pendleton toward a value of 1.00. Other study weaknesses included: (1) almost all of the Marines/Navy personnel subgroup was aged < 65 years by the end of follow-up, reducing numbers of deaths for each cause; and (2) poor precision of HRs for some causes of death in the civilian workers analyses because of the small sample size and resulting small numbers of cases.

Study strengths included: (1) the large sample size of Marines/Navy personnel; (2) forty years of follow-up; (3) a comparison military base with similar demographic and socio-economic and cultural characteristics as Camp Lejeune; (4) a small percentage lost to follow-up; and (5) a majority of civilian workers aged > 65 years by the end of follow-up.

## Conclusion

The Marines/Navy personnel subgroup analysis obtained aHRs ≥ 1.20 with CIRs ≤ 3 for mortality due to cancers of the kidney, esophagus and female breast, and multiple sclerosis (contributing cause). Causes of death with aHRs ≥ 1.20 and CIRs > 3 included Parkinson disease, myelodysplastic syndrome and cancers of the testes, cervix and ovary. In the analyses of the full cohort, additional causes of death with aHRs ≥ 1.20 and CIRs ≤ 3 included Hodgkin lymphoma, AML, all myeloid cancers (contributing cause), and acute kidney disease. Male breast cancer (contributing cause) had an aHR ≥ 1.20 with CIR > 3 in the full cohort. Because most of the Marines/Navy personnel were aged < 65 years by the end of follow-up, additional years of follow-up are necessary to fully evaluate mortality.

The civilian workers analysis obtained aHRs ≥ 1.20 with CIRs ≤ 3 for chronic kidney and Parkinson disease mortality. Female breast cancer as underlying and contributing cause had aHRs of 1.19 and 1.33, respectively, with CIRs ≤ 3. AHRs ≥ 1.20 with CIRs > 3 included cancers of the kidney, pharynx and larynx (as a contributing cause), melanoma, CML, Hodgkin lymphoma and anemias.

Contaminated drinking water exposures at Camp Lejeune affected up to one million people, including Marines/Navy personnel, their families, and civilian workers. The results of this study are relevant to everyone exposed to the contaminated drinking water at Camp Lejeune and add to the literature on the health effects of these chemicals. Because most of the Marines/Navy personnel subgroup were under the age of 65 at the end of follow-up, continued follow-up is indicated.

### Electronic supplementary material

Below is the link to the electronic supplementary material.

Supplementary Material 1

## Data Availability

No datasets were generated or analysed during the current study.

## Abbreviations

aHR: Adjusted hazard ratio
ALS: Amyotrophic lateral sclerosis
AML: Acute myeloid leukemia
ATSDR: Agency for Toxic Substances and Disease Registry
CDC: Centers for Disease Control and Prevention
CI: 95% confidence interval
CIR: Confidence interval ratio
CL: Camp Lejeune
CLL: Chronic lymphocytic leukemia
CML: Chronic myeloid leukemia
CNS: Central nervous system cancers
COPD: Chronic obstructive pulmonary disease
CP: Camp Pendleton
DMDC: Defense Manpower Data Center
HP: Hadnot Point treatment plant
HR: Hazard ratio
ICD: International Classification of Diseases
LTAS: Life table analysis system
MCL: US Environmental Protection Agency maximum contaminant level in drinking water
NDI: National Death Index
MDS: Myelodysplastic syndrome
MS: Multiple sclerosis
NHL: Non-Hodgkin lymphoma
µg/L: Micrograms per liter
PCE: Tetrachloroethylene (also known as perchloroethylene)
QBA: Quantitative bias analyses
RR: Risk ratio
SMR: Standardized mortality ratio
SSA: Social Security Administration
TCE: Trichloroethylene
TT: Tarawa Terrace treatment plant
USMC: United States Marine Corps
