# Supplementary material for: Evaluation of mortality among Marines, Navy personnel, and civilian workers exposed to contaminated drinking water at USMC base Camp Lejeune: a cohort study
Source: Environ Health. 2024 Jul 3;23:61. doi: 10.1186/s12940-024-01099-7 (PMC11221020; doi:10.1186/s12940-024-01099-7)
Supplement: Supplementary file 1 — Supplementary Material 1 [file 12940_2024_1099_MOESM1_ESM.docx]

Supplemental File

**Supplemental File**

**Evaluation of mortality among Marines and Navy personnel and civilian employees exposed to contaminated drinking water at USMC Base Camp Lejeune: a cohort study**

Frank J. Bove, April Greek, Ruth Gatiba, Rona C. Boehm, and Marcie M. Mohnsen

**Table of Contents**

**Description of the quantitative bias analysis methods**.

**Table S1**. Demographic information for the Marines/Navy personnel full cohort at risk during the follow-up period

**Table S2**. Standardized mortality ratios (SMR), Poisson regression risk ratios, and 95% confidence intervals (CI) for the Camp Lejeune and Camp Pendleton Marines/Navy personnel full cohort: Underlying cause of death

**Table S3**. Hazard ratios (HR) and 95% confidence intervals (CI) for the Marines/Navy personnel full cohort analysis of base location at Camp Lejeune (CL) vs. Camp Pendleton (CP): Underlying cause of death

**Table S4**. Hazard ratios (HR) and 95% confidence intervals (CI) for the Marines/Navy personnel full cohort analysis of base location at Camp Lejeune (CL) vs. Camp Pendleton (CP); Contributing causes of death

**Table S5**. Hazard ratios (HR) and 95% confidence intervals (CI) for the Marines/Navy personnel subgroup analysis of base location at Camp Lejeune (CL) vs. Camp Pendleton (CP); Contributing cause of death

**Table S6**. Hazard ratios (HR) and 95% lower and upper confidence intervals (CI) for the Marines/Navy personnel subgroup analysis of base duration between 1975 and 1985 at Camp Lejeune with Camp Pendleton as reference: Underlying cause of death

**Table S7**. Hazard ratios (HR) and 95% confidence intervals (CI) for the analysis of civilian employees at Camp Lejeune (CL) vs. Camp Pendleton (CP): Contributing cause of death

**Table S8**. Hazard ratios (HR) and 95% lower and upper confidence intervals (CI) for the analysis of civilian employees’ employment duration at Camp Lejeune between October 1972 and December 1985 with Camp Pendleton as reference: Underlying cause of death

**Table S9**. Chronic obstructive pulmonary disease (COPD) hazard ratio = 1.08. Adjusted for smoking prevalence (p) differences between the Camp Lejeune and Camp Pendleton Marines/Navy personnel subgroup.

**Table S10**. Kidney cancer hazard ratio = 1.21. Adjusted for a 6% smoking prevalence (p) difference between the Camp Lejeune and Camp Pendleton Marines/Navy personnel subgroup.

**Table S11**. Esophageal cancer hazard ratio = 1.24. Adjusted for a 6% smoking prevalence (p) difference between the Camp Lejeune and Camp Pendleton Marines/Navy personnel subgroup.

**Table S12**. Lung cancer hazard ratio = 1.18. Adjusted for a 6% smoking prevalence (p) difference between the Camp Lejeune and Camp Pendleton Marines/Navy personnel subgroup.

**Table S13**. Parkinson disease hazard ratio = 2.05. Adjusted for a 6% smoking prevalence (p) difference between the Camp Lejeune and Camp Pendleton Marines/Navy personnel subgroup.

**Table S14**. Chronic obstructive pulmonary disease (COPD) hazard ratio = 1.05. Adjusted for smoking prevalence (p) differences between the Camp Lejeune and Camp Pendleton civilian employees.

**Table S15**. Lung cancer hazard ratio = 1.13. Adjusted for a 4% smoking prevalence (p) difference between the Camp Lejeune and Camp Pendleton civilian employees.

**Table S16**. Cancer of the larynx (as an underlying cause) hazard ratio = 1.19. Adjusted for a 4% smoking prevalence (p) difference between the Camp Lejeune and Camp Pendleton civilian employees.

**Table S17**. Cancer of the larynx (as a contributing cause) hazard ratio = 1.69. Adjusted for a 4% smoking prevalence (p) difference between Camp Lejeune and Camp Pendleton civilian employees.

**Table S18**. Pharyngeal cancer hazard ratio = 2.21. Adjusted for a 4% smoking prevalence (p) difference between the Camp Lejeune and Camp Pendleton civilian employees.

**Table S19**. Kidney cancer hazard ratio = 1.44. Adjusted for a 4% smoking prevalence (p) difference between the Camp Lejeune and Camp Pendleton civilian employees.

**Table S20**. Chronic kidney disease hazard ratio = 1.88. Adjusted for a 4% smoking prevalence (p) difference between the Camp Lejeune and Camp Pendleton civilian employees.

**Table S21**. Parkinson disease hazard ratio = 1.21. Adjusted for a 4% smoking prevalence (p) difference between the Camp Lejeune and Camp Pendleton civilian employees.

**Table S22**. Chronic liver disease mortality (as an underlying cause) hazard ratio = 0.93. Adjusted for alcohol consumption prevalence (p) differences between the Camp Lejeune and Camp Pendleton Marines/Navy personnel subgroup.

**Table S23**. Esophageal cancer mortality hazard ratio = 1.24. Adjusted for a 8% alcohol consumption prevalence (p) difference between the Camp Lejeune and Camp Pendleton Marines/Navy personnel subgroup.

**Table S24**. Cancer of the larynx mortality (as a contributing cause) hazard ratio = 1.14. Adjusted for a 8% alcohol consumption prevalence (p) difference between the Camp Lejeune and Camp Pendleton Marines/Navy personnel subgroup.

**Table S25**. Female breast cancer mortality hazard ratio = 1.20. Adjusted for a 8% alcohol consumption prevalence (p) difference between the Camp Lejeune and Camp Pendleton Marines/Navy personnel subgroup.

**Table S26**. Chronic liver disease mortality hazard ratio = 0.74. Adjusted for alcohol consumption prevalence (p) differences between the Camp Lejeune and Camp Pendleton civilian employees.

**Table S27**. Oral cancer mortality (as a contributing cause) hazard ratio = 1.12. Adjusted for a 15% alcohol consumption prevalence (p) difference between the Camp Lejeune and Camp Pendleton civilian employees.

**Table S28**. Cancer of the larynx hazard ratio = 1.19. Adjusted for a 15% alcohol consumption prevalence (p) difference between the Camp Lejeune and Camp Pendleton civilian employees.

**Table S29**. Cancer of the pharynx hazard ratio = 2.21. Adjusted for a 15% alcohol consumption prevalence (p) difference between the Camp Lejeune and Camp Pendleton civilian workers.

**Table S30**. Female breast cancer mortality hazard ratio = 1.19. Adjusted for a 15% alcohol consumption prevalence (p) difference between the Camp Lejeune and Camp Pendleton civilian employees.

**Table S31**: Increases in the hazard ratio (HR) accounting for non-differential exposure misclassification: Marines/Navy personnel subgroup

**Table S32**. Increases in the hazard ratio (HR) accounting for non-differential exposure misclassification: Civilian employees

**References**

**Description of the quantitative bias analysis methods**

Quantitative bias analyses were conducted to estimate quantitatively, and adjust the HR estimates for, the systematic errors (or biases) due to unmeasured confounding factors and exposure misclassification. The analyses focused on the dichotomous subgroup comparisons between Camp Lejeune and Camp Pendleton, and used Excel spreadsheets included with the textbook, Applying Quantitative Bias Analysis to Epidemiologic Data, Second Edition [1]. A quantitative bias analysis involves choosing a bias model (e.g., exposure misclassification), an analytic technique (e.g., a multidimensional analysis), and values for the parameters of the bias model (e.g., for exposure misclassification, the bias parameters could be the sensitivity and specificity of the exposure classification). The values of the bias parameters are applied to the observed data using bias adjustment equations to calculate what the data would have been if the bias were absent. The quantitative bias analyses of the impacts of unmeasured confounding due to smoking and alcohol consumption used the negative control results to determine the values for the bias parameters of the bias model.

Quantitative bias analyses of exposure misclassification assumed that the misclassification was non-differential and independent because base locations were assigned to the Marines/Navy personnel and civilian workers prior to the mortality data collection.

For Camp Lejeune Marines/Navy personnel, the sources of possible exposure misclassification were due to using unit assignment to a base as a proxy for exposure to the drinking water. First, errors were possible in the historical research conducted by the DMDC and USMC to determine the base where each unit was located. Second, even if the base assignment of the unit was correct, some individuals may not have been exposed to the contaminated drinking water because they were deployed to a different base (e.g., outside the country) or trained at a different base. Third, some individuals stationed at Camp Lejeune may not have been exposed because all their water consumption (including showering and other water uses) occurred off-base (e.g., in off-base housing) or in areas of the base not served by the HP or TT drinking water systems. On the other hand, virtually all of those classified as stationed at Camp Pendleton likely were truly unexposed to the contaminated drinking water.

For Camp Lejeune civilian employees, the main source of exposure misclassification was due to water consumption (including showering and other water uses) occurring mostly or entirely off-base (e.g., at their residences). In addition, the workplaces of some of the Camp Lejeune civilian employees may have been located in areas not served by the contaminated drinking water. All civilian employees at Camp Pendleton were assumed to be truly unexposed to contaminated drinking water during the study period.

To conduct the quantitative bias analyses, it was assumed that the sensitivity of the exposure classification for the Marines/Navy personnel and civilian employees, i.e., the probability that the truly exposed individuals were correctly classified as exposed (i.e., assigned to Camp Lejeune) was 1.0. The specificity of the exposure classification, i.e., the probability that the truly unexposed individuals were correctly classified as unexposed (i.e., assigned to Camp Pendleton) was assumed to range from 0.81 to 0.91. The chosen values for sensitivity and specificity used in the quantitative bias analysis reflected the assumptions that between 75% and 90% of those stationed or employed at Camp Lejeune were truly exposed, and all of those stationed or employed at Camp Pendleton were truly unexposed.

The quantitative bias analyses of possible confounding due to smoking and alcohol consumption used the results of the smoking-related and alcohol-related negative control diseases, i.e., mortality due to COPD, cardiovascular disease, alcoholism, alcoholic liver disease and chronic liver disease, to estimate prevalence differences in smoking and alcohol consumption between Camp Lejeune and Camp Pendleton. The results of the Cox regression analyses of the negative control diseases for the Marines/Navy personnel subgroup and civilian employees are shown in Tables 4 – 5 (underlying causes) in the main text and Tables S5 and S7 (contributing causes) in the supplemental file.

For the Marines/Navy personnel subgroup, the smoking-related negative control diseases COPD and cardiovascular disease had adjusted HRs of 1.08 and 0.99, respectively as underlying causes of death. (Lower HRs were found for these diseases as contributing causes of death.) The bias analysis assumed that about half or more of the Marines smoked, based on a 1980 survey that found that 53.4% of Marines smoked [2]. Using a range of RRs from 3.00 to 5.50 for smoking and COPD [3], to fully explain the HR of 1.08 for COPD, the difference in smoking prevalence between Camp Lejeune and Camp Pendleton Marines/Navy personnel would be about 6% (Table S9). (Using higher RRs for smoking and COPD would lower the prevalence difference between Camp Lejeune and Camp Pendleton resulting in a lower impact of confounding bias due to smoking.)

Assuming a 6% difference in smoking prevalence and a range of RRs for smoking and kidney cancer between 1.25 and 1.75 [4-5], the observed adjusted HR for kidney cancer of 1.21 for the Marines/Navy personnel subgroup would be reduced to between 1.17 and 1.19, a change of ≤3.3% (Table S10). Assuming a range of RRs for smoking and esophageal cancer between 1.5 and 4.5 [4,6], the observed adjusted HR of 1.24 would be reduced to between 1.15 and 1.21, a change of ≤7.3% (Table S11).

Since smoking is a strong risk factor for lung cancer, the impact of adjusting for smoking on the observed adjusted HR for lung cancer should be the greatest. Assuming a 6% prevalence difference in smoking and assuming that the RR for smoking and lung cancer ranges between 7.00 and 12.00 [4], the observed adjusted HR of 1.18 for lung cancer as an underlying cause would be reduced to between 1.07 and 1.08, a change of ≤9.3% (Table S12). Smoking has been observed to decrease the risk of Parkinson disease [7-8]. Adjusting for smoking and assuming a range of RRs for smoking and Parkinson disease between 0.30 and 0.85 [7-8], the observed adjusted HR of 2.05 for the Marines/Navy personnel subgroup would increase to between 2.07 and 2.19, a change of ≤6.8% (Table S13).

For smoking to fully explain the HR for COPD of 1.05 for civilian employees, the difference in smoking prevalence between Camp Lejeune and Camp Pendleton would be no more than 4% (Table S14). Adjusting for a smoking prevalence difference of 4% and assuming RRs for smoking and cancers of the lung and larynx ranging between 7.00 and 12.00 [4,9], the underlying cause HRs of 1.13 for lung cancer and 1.19 for laryngeal cancer would decrease by <7.1% (Tables S15-S16). The HR for laryngeal cancer as a contributing cause of 1.69 would also decrease by ≤7.1% (Table S17).

For civilian employees, the adjusted HR for cancer of the pharynx as an underlying cause would decrease from 2.21 to 2.07, or about 6.3%, assuming the RRs for smoking and cancer of the pharynx ranges from 5.0 to 7.5 [4] (Table S18). Assuming RRs for smoking and kidney cancer and chronic kidney disease ranging from 1.30 to 1.80 [4,9], the adjusted HRs of 1.44 for kidney cancer and 1.88 chronic kidney disease would decrease by ≤2.8% (Tables S19-S-20). Assuming RRs for smoking and Parkinson disease ranging between 0.30 and 0.85 [7-8], the underlying cause HR for Parkinson disease of 1.21 would increase by ≤4.1% (Table S21).

A military survey conducted in 1980 found that about 30% of Marines were heavy drinkers defined as drinking five or more drinks per typical drinking occasion at least once a week in the past 30 days [2]. To estimate the alcohol consumption prevalence differences between the Camp Lejeune and Camp Pendleton Marines/Navy personnel subgroup, the bias analysis used the adjusted HR of 0.93 for chronic liver disease mortality (Table 4, main text).

A recent systematic review of alcohol consumption and mortality due to liver cirrhosis found RRs of 2.65, 6.83 and 16.38 for drinking 25g/day (2 drinks/day), 50g/day (4 drinks/day) and 100g/day (8 drinks/day) compared to those who never drank alcoholic beverages [10]. Assuming at least 2/3 of Marines/Navy personnel at Camp Lejeune consumed ≥1 drink/day and assuming that the RRs for alcohol consumption and chronic liver disease mortality ranged between 2.5 and 10.0 [10], to fully explain the RR of 0.93, the prevalence difference would range between 6% and 10% (Table S22). (Assuming a lower percentage of Camp Lejeune drinkers would decrease the prevalence difference range, e.g., if only half the Marines/Navy personnel at Camp Lejeune were drinkers, then the percentage difference range would be 5% - 9%)

Adjusting for an alcohol prevalence difference of 8% between Camp Lejeune and Camp Pendleton Marines/Navy personnel, and assuming RRs for alcohol consumption and esophageal cancer ranging from 1.25 to 5.25 [11-12], the HR of 1.24 for esophageal cancer as an underlying cause would increase to between 1.26 and 1.35, or by ≤8.9% (Table S23). The HR of 1.14 for laryngeal cancer as a contributing cause would increase to between 1.15 and 1.22, or ≤7.0% (Table S24). Assuming RRs for alcohol consumption and female breast cancer range from 1.10 to 1.60 [12], the female breast cancer HR of 1.20 as an underlying cause would increase to between 1.21 and 1.24, or by ≤3.3% (Table S25).

For the civilian employees, the adjusted HR for chronic liver disease mortality as an underlying cause was 0.74. To fully explain this HR, the prevalence difference in alcohol consumption between Camp Lejeune and Camp Pendleton employees would range between 15% and 25%, assuming that about 1/3 of the Camp Lejeune employees consumed ≥1 drink/day and assuming that the RRs for alcohol consumption and chronic liver disease mortality range between 2.5 and 10.0 [10] (Table S26). (Assuming that only 20% of Camp Lejeune employees consumed ≥1 drink/day, the prevalence difference would range from 11% to 21%. Assuming a higher percentage of Camp Lejeune drinkers would increase the prevalence difference range, e.g., if 50% of Camp Lejeune employees consumed ≥1 drink/day, the prevalence difference would range from 21% to 31%.)

Adjusting for an alcohol prevalence difference of 15% between Camp Lejeune and Camp Pendleton employees, and assuming RRs for alcohol consumption and oral cancers range from 1.10 to 5.20 [12], the HR of 1.12 for oral cancers as a contributing cause would increase to between 1.13 and 1.41, or by ≤25.9% (Table S27). Assuming RRs for alcohol consumption and laryngeal cancer range from 1.10 to 3.00 [12], the HR of 1.19 for laryngeal cancer as an underlying cause would increase to between 1.20 and 1.40, or by ≤17.6% (Table S28). Assuming RRs for alcohol consumption and pharyngeal cancer range from 1.10 to 5.2 [12], the HR of 2.21 for pharyngeal cancer would increase to between 2.24 and 2.79, or by ≤26% (Table S29). Assuming RRs for alcohol consumption and female breast cancer range from 1.10 to 1.60 [12], the HR of 1.19 for female breast cancer as an underlying cause would increase to between 1.20 and 1.27, or by ≤6.7% (Table S30).

The impact of non-differential exposure misclassification on the adjusted HRs for the Marines/Navy personnel and civilian employees assumed that between 10% and 25% of those assigned to Camp Lejeune were truly unexposed and virtually none of those assigned to Camp Pendleton were truly exposed (Tables S31-S32).

For underlying cause of death in the Marines/Navy personnel subgroup, after accounting for exposure misclassification the observed adjusted HR for kidney cancer of 1.21 would increase to between 1.23 and 1.27, or by ≤5% (Table S31). For esophageal cancer, the observed adjusted HR of 1.24 would increase to between 1.27 and 1.32, or by ≤6.5%. For Parkinson disease, the observed adjusted HR of 2.05 would increase to between 2.17 and 2.40, or by ≤17.1%. For lung cancer, the observed adjusted HR of 1.18 would increase to between 1.20 and 1.23, or by ≤4.2% (Table S31).

For civilian employees, adjusting for non-differential exposure misclassification would increase the underlying cause HRs for lung cancer and female breast cancer by <3.5% (Table S32). However, the underlying cause HR for kidney cancer would increase by ≤12.5%, and the underlying cause HR for chronic kidney disease would increase by ≤13.3%. The underlying cause HR for Parkinson disease would increase by ≤5% and the contributing cause HR for female breast cancer would increase by ≤6% (Table S32).

**Table S1**. Demographic information for the Marines/Navy personnel full cohort at risk during the follow-up period

| Base | Camp Lejeune N (%) | Camp Pendleton N (%) (ref) | Total N (%) |
| --- | --- | --- | --- |
| Marines at risk | 217,988 (48.4) | 232,026 (51.6) | 450,014 |
|  |  |  |  |
| Sex |  |  |  |
| Male | 209,127 (95.9) | 225,166 (97.0) | 434,293 (96.5) |
| Female | 8,861 (4.1) | 6,860 (3.0) | 15,721 (3.5) |
|  |  |  |  |
| Race |  |  |  |
| “white” | 161,410 (74.0) | 184,340 (79.4) | 345,750 (76.8) |
| African  American | 51,673 (23.7) | 37,190 (16.0) | 88,863 (19.7) |
| Other race | 4,905 (2.3) | 10,496 (4.5) | 15,401 (3.4) |
|  |  |  |  |
| Rank |  |  |  |
| E1 – E4 | 157,364 (72.2) | 164,387 (70.8) | 321,751 (71.5) |
| E5 – E9 | 48,202 (22.1) | 51,741 (22.3) | 99,943 (22.2) |
| WO or CO | 12,422 (5.7) | 15,898 ( 6.9) | 28,320 (6.3) |
|  |  |  |  |
| Education |  |  |  |
| High school graduate | 171,031 (78.5) | 175,186 (75.5) | 346,217 (76.9) |
| <High school | 35,782 (16.4) | 42,096 (18.1) | 77,878 (17.3) |
| College graduate and higher | 11,175 (5.1) | 14,744 (6.4) | 25,919 (5.8) |
|  |  |  |  |
| Age, at start of follow-up (1/1/1979) |  |  |  |
| Mean | 22.1 years | 22.4 years | 22.3 years |
| Median | 21.0 years | 21.0 years | 21.0 years |
|  |  |  |  |
| Age at end of follow-up^*^ |  |  |  |
| Mean | 58.4 years | 58.7 years | 58.6 years |
| Median | 59.0 years | 59.0 years | 59.0 years |
| % Age >55 years | 72.4% | 73.8% | 73.1% |
| % Age >65 years | 11.0% | 12.3% | 11.6% |
|  |  |  |  |
| Deaths^¥^ |  |  |  |
| Number | 34,652 | 36,892 | 71,544 |
| % of cohort | 15.9% | 15.9% | 15.9% |
| Base | Camp Lejeune | Camp Pendleton  (ref) | Total |
| Length of follow-up (years) |  |  |  |
| Mean | 36.3 | 36.3 | 36.3 |
| Median | 39.0 | 39.0 | 39.0 |
|  |  |  |  |
| Total person-years of follow-up | 7,912,152 | 8,413,589 | 16,325,741 |
| Total lost to follow-up | 1,582 (0.7%) | 1,838 (0.8%) | 3,420 (0.8%) |

E1 – E4: private to corporal

E5 – E9: sergeant to sergeant major

WO: warrant officer

CO: commissioned officer

^*^ Age at end of follow-up (12/31/2018 or date of death if earlier than 12/31/2018)

^¥^ Deaths occurring 1/1/1979 – 12/31/2018

**Table S2**. Standardized mortality ratios (SMR), Poisson regression risk ratios, and 95% confidence intervals (CI) for the Camp Lejeune and Camp Pendleton Marines/Navy personnel full cohort: Underlying cause of death

| **Cause of Death** | **Camp Lejeune (CL)** | **Camp Pendleton (CP)** | **Risk Ratio (95% CI)** |
| --- | --- | --- | --- |
|  | Observed SMR (95% CI) | Observed SMR (95% CI) | **CL vs CP** |
| All Causes | 34,652 0.95 (0.94, 0.96) | 36,885 0.94 (0.93, 0.95) | 1.02 (1.01, 1.04) |
| All Cancer Malignancies | 7,932 1.00 (0.97, 1.02) | 8,164 0.94 (0.92, 0.96) | 1.07 (1.04, 1.10) |
| Oral Cavity and Pharynx | 226 0.98 (0.86, 1.12) | 256 1.03 (0.91, 1.16) | 0.99 (0.82, 1.18) |
| Esophagus | 337 1.01 (0.90, 1.12) | 355 0.95 (0.86, 1.06) | 1.07 (0.92, 1.24) |
| Stomach | 184 0.85 (0.73, 0.98) | 206 0.91 (0.79, 1.05) | 0.95 (0.78, 1.16) |
| Colon | 514 0.87 (0.80, 0.95) | 569 0.89 (0.82, 0.97) | 0.97 (0.86, 1.10) |
| Rectum | 155 0.84 (0.71, 0.98) | 170 0.85 (0.73, 0.99) | 0.98 (0.79, 1.22) |
| Liver/Biliary System | 509 0.90 (0.82, 0.98) | 576 0.96 (0.89, 1.04) | 0.96 (0.86, 1.09) |
| Pancreas | 544 1.09 (1.00, 1.19) | 533 0.98 (0.90, 1.07) | 1.12 (0.99, 1.26) |
| Larynx | 97 1.01 (0.82, 1.24) | 94 0.91 (0.74, 1.11) | 1.15 (0.86, 1.52) |
| Lung/Trachea/Bronchus | 2,450 1.10 (1.06, 1.14) | 2,378 0.98 (0.94, 1.02) | 1.15 (1.08, 1.21) |
| Cervix | 9 0.94 (0.43, 1.79) | 5 0.65 (0.21, 1.51) | 1.23 (0.40, 3.80) |
| Uterus | 9 1.31 (0.60, 2.48) | 7 1.14 (0.46, 2.34) | 1.17 (0.43, 3.17) |
| Ovary | 12 0.86 (0.45, 1.51) | 13 1.04 (0.55, 1.77) | 0.99 (0.44, 2.21) |
| Breast Cancer - Female | 53 0.99 (0.74, 1.30) | 31 0.68 (0.46, 0.96) | 1.43 (0.91, 2.23) |
| Breast Cancer - Male | 13 1.14 (0.60, 1.94) | 12 0.97 (0.50, 1.69) | 1.15 (0.53, 2.53) |
| Prostate | 321 1.12 (1.00, 1.24) | 312 1.01 (0.90, 1.13) | 1.11 (0.95, 1.30) |
| Testis | 30 0.81 (0.53, 1.18) | 20 0.55 (0.33, 0.84) | 1.73 (0.98, 3.05) |
| Kidney and Renal Pelvis | 279 1.13 (1.00, 1.27) | 253 0.93 (0.82, 1.05) | 1.22 (1.03, 1.45) |
| Urinary Bladder | 137 0.90 (0.76, 1.07) | 171 1.01 (0.87, 1.18) | 0.90 (0.72, 1.13) |
| Melanoma | 191 0.98 (0.85, 1.13) | 221 0.99 (0.86, 1.12) | 1.00 (0.82, 1.21) |
| Connective Tissue | 82 0.99 (0.78, 1.22) | 76 0.84 (0.66, 1.05) | 1.18 (0.86, 1.61) |
| Brain and CNS | 315 0.96 (0.85, 1.07) | 350 0.95 (0.85, 1.05) | 1.01 (0.87, 1.17) |
| Thyroid | 17 0.87 (0.51, 1.40) | 22 1.02 (0.64, 1.54) | 0.85 (0.45, 1.60) |
| Hematopoietic Cancers | 730 0.90 (0.84, 0.97) | 746 0.84 (0.78, 0.90) | 1.07 (0.96, 1.18) |
| Hodgkin Lymphoma | 49 1.01 (0.75, 1.33) | 41 0.77 (0.55, 1.04) | 1.29 (0.85, 1.95) |
| NHL | 263 0.83 (0.73, 0.93) | 300 0.85 (0.76, 0.96) | 0.96 (0.81, 1.13) |
| Multiple Myeloma | 140 1.04 (0.88, 1.23) | 129 0.89 (0.74, 1.06) | 1.15 (0.91, 1.47) |
| Leukemias | 284 0.92 (0.82, 1.04) | 277 0.82 (0.73, 0.92) | 1.13 (0.95, 1.33) |
| Diabetes | 833 0.81 (0.75, 0.86) | 899 0.81 (0.76, 0.87) | 0.99 (0.90, 1.09) |
| Alcoholism | 391 0.96 (0.87, 1.06) | 450 1.03 (0.93, 1.12) | 0.94 (0.82, 1.08) |
| Multiple Sclerosis | 53 0.85 (0.65, 1.12) | 43 0.63 (0.46, 0.84) | 1.32 (0.88, 1.98) |
| Parkinson Disease | 64 1.01 (0.76, 1.26) | 67 0.94 (0.72, 1.17) | 1.06 (0.75, 1.49) |
| ALS | 116 1.08 (0.88, 1.28) | 130 1.07 (0.89, 1,26) | 1.00 (0.78, 1.28) |
| Cardiovascular Disease | 8,683 0.95 (0.93, 0.97) | 8,922 0.91 (0.89, 0.92) | 1.05 (1.02, 1.09) |
| COPD | 879 1.04 (0.97, 1.11) | 982 1.04 (0.98, 1.11) | 1.02 (0.93, 1.11) |
| Liver Disease | 1,047 0.84 (0.79, 0.89) | 1,320 0.95 (0.90, 1.00) | 0.87 (0.81, 0.95) |
| **Cause of Death** | **Camp Lejeune (CL)** | **Camp Pendleton (CP)** | **Risk Ratio (95% CI)** |
|  | Observed SMR (95% CI) | Observed SMR (95% CI) | **CL vs CP** |
| Kidney Disease | 313 0.76 (0.68, 0.85) | 300 0.70 (0.63, 0.78) | 1.09 (0.93, 1.27) |
| Suicide | 2,210 1.18 (1.13, 1.23) | 2,717 1.29 (1.25, 1.34) | 0.91 (0.86, 0.96) |

CNS: central nervous system

NHL: non-Hodgkin lymphoma

ALS: amyotrophic lateral sclerosis

COPD: chronic obstructive pulmonary disease

Risk ratios adjusted for sex, race, and 5-year age groups.

**Table S3**. Hazard ratios (HR) and 95% confidence intervals (CI) for the Marines/Navy personnel full cohort analysis of base location at Camp Lejeune (CL) vs. Camp Pendleton (CP): Underlying cause of death

| Outcome | Total | Camp Lejeune # | Unadjusted  HR (95% CI) | Adjusted  HR (95% CI) | Camp Pendleton # |
| --- | --- | --- | --- | --- | --- |
| All causes | 71,544 | 34,652 | 1.02 (1.00, 1.03) | 1.01 (0.99, 1.02) | 36,892 |
| All cancer malignancies | 16,096 | 7,932 | 1.06 (1.03, 1.10) | 1.05 (1.02, 1.08) | 8,164 |
| Oral cancers | 482 | 226 | 0.96 (0.81, 1.15) | 0.97 (0.81, 1.16) | 256 |
| Pharyngeal cancer | 257 | 123 | 1.00 (0.79, 1.28) | 0.99 (0.78, 1.27) | 134 |
| Esophageal cancer | 692 | 337 | 1.04 (0.89, 1.20) | 1.04 (0.90, 1.21) | 355 |
| Stomach cancers | 390 | 184 | 0.98 (0.80, 1.19) | 0.94 (0.77, 1.14) | 206 |
| Colorectal cancers | 1,408 | 669 | 0.99 (0.89, 1.10) | 0.96 (0.86, 1.07) | 739 |
| Colon cancer | 1,083 | 514 | 0.99 (0.88, 1.11) | 0.95 (0.85, 1.08) | 569 |
| Rectal cancer | 325 | 155 | 0.99 (0.80, 1.23) | 0.98 (0.79, 1.22) | 170 |
| Liver cancer | 1,085 | 509 | 0.97 (0.86, 1.09) | 0.97 (0.86, 1.09) | 576 |
| Pancreatic cancer | 1,077 | 544 | 1.12 (0.99, 1.26) | 1.10 (0.97, 1.24) | 533 |
| Laryngeal cancer | 191 | 97 | 1.13 (0.85, 1.50) | 1.10 (0.83, 1.46) | 94 |
| Lung cancer | 4,828 | 2,450 | 1.13 (1.07, 1.20) | 1.12 (1.06, 1.18) | 2,378 |
| Bone cancers | 59 | 24 | 0.74 (0.44, 1.24) | 0.75 (0.45, 1.27) | 35 |
| Soft tissue cancers | 158 | 82 | 1.17 (0.86, 1.60) | 1.14 (0.83, 1.56) | 76 |
| Melanoma | 412 | 191 | 0.94 (0.77, 1.14) | 1.00 (0.82, 1.21) | 221 |
| Female Breast cancer | 84 | 53 | 1.45 (0.93, 2.27) | 1.41 (0.90, 2.21) | 31 |
| Male Breast cancer | 25 | 13 | 1.18 (0.54, 2.58) | 1.09 (0.50, 2.41) | 12 |
| Cervical cancer | 14 | 9 | 1.26 (0.41, 3.85) | 1.30 (0.42, 4.01) | 5 |
| Uterine cancer | 16 | 9 | 1.21 (0.45, 3.26) | 1.17 (0.43, 3.16) | 7 |
| Ovarian cancer | 25 | 12 | 0.95 (0.42, 2.12) | 1.02 (0.46, 2.30) | 13 |
| Prostate cancer | 633 | 321 | 1.14 (0.98, 1.33) | 1.06 (0.91, 1.24) | 312 |
| Testicular cancer | 50 | 30 | 1.62 (0.92, 2.85) | 1.82 (1.03, 3.21) | 20 |
| Bladder cancer | 308 | 137 | 0.88 (0.70, 1.10) | 0.89 (0.71, 1.11) | 171 |
| Kidney cancer | 532 | 279 | 1.20 (1.01, 1.43) | 1.21 (1.02, 1.43) | 253 |
| Brain and CNS cancers | 665 | 315 | 0.98 (0.84, 1.14) | 1.01 (0.86, 1.17) | 350 |
| Thyroid cancer | 39 | 17 | 0.85 (0.45, 1.59) | 0.82 (0.44, 1.56) | 22 |
| Hematopoietic cancers | 1,476 | 730 | 1.07 (0.96, 1.18) | 1.04 (0.94, 1.16) | 746 |
| Hodgkin lymphoma | 90 | 49 | 1.29 (0.85, 1.95) | 1.25 (0.82, 1.90) | 41 |
| Non-Hodgkin lymphoma | 563 | 263 | 0.95 (0.81, 1.13) | 0.95 (0.81, 1.13) | 300 |
| Multiple myeloma | 269 | 140 | 1.19 (0.94, 1.51) | 1.12 (0.88, 1.42) | 129 |
| Leukemias | 561 | 284 | 1.11 (0.94, 1.31) | 1.10 (0.93, 1.30) | 277 |
| ALL | 71 | 35 | 1.05 (0.66, 1.67) | 1.07 (0.67, 1.71) | 36 |
| CLL | 66 | 26 | 0.71 (0.44, 1.17) | 0.68 (0.41, 1.11) | 40 |
| AML | 237 | 125 | 1.21 (0.94, 1.57) | 1.21 (0.94, 1.56) | 112 |
| CML | 55 | 23 | 0.77 (0.45, 1.32) | 0.73 (0.43, 1.25) | 32 |
| MDS | 59 | 32 | 1.31 (0.78, 2.18) | 1.36 (0.81, 2.28) | 27 |
| Outcome | Total | Camp Lejeune # | Unadjusted  HR (95% CI) | Adjusted  HR (95% CI) | Camp Pendleton # |
| Lymphoid cancers | 143 | 58 | 0.80 (0.57, 1.11) | 0.79 (0.56. 1.10) | 85 |
| Myeloid cancers | 307 | 155 | 1.18 (0.94, 1.48) | 1.16 (0.92, 1.45) | 152 |
| Diabetes | 1,732 | 833 | 1.02 (0.92, 1.12) | 0.99 (0.90, 1.09) | 899 |
| Anemias | 53 | 25 | 0.98 (0.57, 1.67) | 0.91 (0.53, 1.57) | 28 |
| Cardiovascular disease | 17,605 | 8,683 | 1.06 (1.03, 1.09) | 1.03 (1.00, 1.06) | 8,922 |
| Heart disease | 14,391 | 7,050 | 1.05 (1.01, 1.08) | 1.02 (0.99, 1.05) | 7,341 |
| Stroke | 1,766 | 908 | 1.15 (1.05, 1.27) | 1.09 (0.99, 1.20) | 858 |
| Circulatory diseases | 1,447 | 724 | 1.09 (0.99, 1.21) | 1.03 (0.93, 1.15) | 723 |
| COPD | 1,861 | 879 | 0.99 (0.90, 1.09) | 0.99 (0.90, 1.08) | 982 |
| Chronic Liver disease | 2,367 | 1,047 | 0.86 (0.79, 0.93) | 0.90 (0.83, 0.98) | 1,320 |
| Cirrhosis | 2,660 | 1,182 | 0.87 (0.80, 0.94) | 0.91 (0.84, 0.98) | 1,478 |
| Alcoholic Liver disease | 1,486 | 616 | 0.77 (0.69, 0.85) | 0.82 (0.74, 0.91) | 870 |
| Nonalcoholic Liver disease | 881 | 431 | 1.04 (0.91, 1.18) | 1.07 (0.94, 1.22) | 450 |
| Acute Kidney disease | 117 | 65 | 1.37 (0.95, 1.97) | 1.32 (0.91, 1.90) | 52 |
| Chronic Kidney disease | 614 | 313 | 1.14 (0.97, 1.33) | 1.04 (0.88, 1.22) | 301 |
| Parkinson disease | 131 | 64 | 1.30 (0.92, 1.83) | 1.29 (0.92, 1.82) | 67 |
| ALS | 246 | 116 | 0.98 (0.76, 1.25) | 0.99 (0.77, 1.28) | 130 |
| Multiple sclerosis | 96 | 53 | 1.38 (0.93, 2.07) | 1.37 (0.92, 2.06) | 43 |
| Alcoholism | 841 | 391 | 0.94 (0.82, 1.07) | 0.95 (0.83, 1.09) | 450 |
| Suicide | 4,927 | 2,210 | 0.87 (0.82, 0.92) | 0.91 (0.86, 0.96) | 2,717 |

CL = 217,988 Males = 209,127 Females = 8,861

CP = 232,026 Males = 225,166 Females = 6,860

Total = 450,014 Males = 434,293 Females = 15,721

COPD: chronic obstructive pulmonary disease

ALS: amyotrophic lateral sclerosis

MDS: myelodysplastic syndrome

CML: chronic myeloid leukemia

AML: acute myeloid leukemia

CLL: chronic lymphocytic leukemia

ALL: acute lymphocytic leukema

CNS: central nervous system

HRs adjusted for sex, race, rank and education level; age was the time variable.

**Table S4**. Hazard ratios (HR) and 95% confidence intervals (CI) for the Marines/Navy personnel full cohort analysis of base location at Camp Lejeune (CL) vs. Camp Pendleton (CP); Contributing causes of death

| Outcome | Total | | Camp Lejeune # | Unadjusted  HR (95% CI) | Adjusted  HR (95% CI) | Camp Pendleton # |
| --- | --- | --- | --- | --- | --- | --- |
| all cancer malignancies | 17,258 | | 8,480 | 1.06 (1.03, 1.09) | 1.04 (1.01, 1.08) | 8,778 |
| Oral cancers | 553 | | 256 | 0.94 (0.80, 1.11) | 0.95 (0.80, 1.12) | 297 |
| Pharyngeal cancer | 305 | | 147 | 1.02 (0.81, 1.27) | 1.01 (0.80, 1.26) | 158 |
| Esophageal cancer | 740 | | 360 | 1.04 (0.90, 1.20) | 1.04 (0.90, 1.20) | 380 |
| Stomach cancers | 408 | | 195 | 1.00 (0.82, 1.21) | 0.96 (0.79, 1.17) | 213 |
| Colorectal cancers | | 1,517 | 723 | 0.99 (0.90, 1.10) | 0.97 (0.87, 1.07) | 794 |
| Colon cancer | 1,168 | | 556 | 0.99 (0.89, 1.11) | 0.96 (0.85, 1.08) | 612 |
| Rectal cancer | 354 | | 168 | 0.98 (0.80, 1.21) | 0.97 (0.79, 1.20) | 186 |
| Liver cancer | 1,216 | | 570 | 0.97 (0.86, 1.08) | 0.96 (0.86, 1.08) | 646 |
| Pancreatic cancer | 1,120 | | 567 | 1.12 (1.00, 1.26) | 1.10 (0.98, 1.24) | 553 |
| Laryngeal cancer | 228 | | 117 | 1.16 (0.89, 1.50) | 1.13 (0.87, 1.47) | 111 |
| Lung cancer | 5,050 | | 2,560 | 1.13 (1.07, 1.19) | 1.12 (1.06, 1.18) | 2,490 |
| Bone cancers | 63 | | 25 | 0.71 (0.43, 1.17) | 0.72 (0.43, 1.19) | 38 |
| Soft tissue cancers | 173 | | 88 | 1.12 (0.83, 1.51) | 1.09 (0.81, 1.47) | 85 |
| Melanoma | 434 | | 203 | 0.95 (0.79, 1.15) | 1.01 (0.84, 1.22) | 231 |
| Female Breast cancer | 98 | | 60 | 1.38 (0.92, 2.07) | 1.34 (0.89, 2.02) | 38 |
| Male Breast cancer | 29 | | 16 | 1.34 (0.64, 2.78) | 1.27 (0.61, 2.64) | 13 |
| Cervical cancer | 14 | | 9 | 1.26 (0.41, 3.85) | 1.30 (0.42, 4.01) | 5 |
| Uterine cancer | 16 | | 9 | 1.21 (0.45, 3.26) | 1.17 (0.43, 3.16) | 7 |
| Ovarian cancer | 27 | | 13 | 0.95 (0.44, 2.06) | 1.02 (0.47, 2.22) | 14 |
| Prostate cancer | 835 | | 419 | 1.12 (0.98, 1.28) | 1.04 (0.90, 1.19) | 416 |
| Testicular cancer | 65 | | 39 | 1.63 (0.99, 2.67) | 1.81 (1.10, 2.97) | 26 |
| Bladder cancer | 367 | | 166 | 0.91 (0.74, 1.12) | 0.91 (0.74, 1.12) | 201 |
| Kidney cancer | 593 | | 305 | 1.16 (0.98, 1.36) | 1.16 (0.98, 1.36) | 288 |
| Brain and CNS cancers | 689 | | 328 | 0.99 (0.85, 1.14) | 1.02 (0.87, 1.18) | 361 |
| Thyroid cancer | 51 | | 22 | 0.83 (0.48, 1.44) | 0.81 (0.47, 1.42) | 29 |
| Hematopoietic cancers | 1,762 | | 864 | 1.05 (0.96, 1.15) | 1.02 (0.93, 1.13) | 898 |
| Hodgkin lymphoma | 135 | | 73 | 1.27 (0.91, 1.78) | 1.25 (0.89, 1.76) | 62 |
| Non-Hodgkin lymphoma | 691 | | 323 | 0.96 (0.82, 1.11) | 0.95 (0.82, 1.10) | 368 |
| Multiple myeloma | 314 | | 161 | 1.16 (0.93, 1.44) | 1.08 (0.86, 1.35) | 153 |
| Leukemias | 664 | | 339 | 1.14 (0.98, 1.32) | 1.11 (0.96, 1.30) | 325 |
| ALL | 75 | | 37 | 1.05 (0.67, 1.65) | 1.06 (0.67, 1.68) | 38 |
| CLL | 98 | | 40 | 0.76 (0.51, 1.14) | 0.73 (0.48, 1.09) | 58 |
| AML | 264 | | 141 | 1.25 (0.98, 1.59) | 1.24 (0.97, 1.58) | 123 |
| CML | 67 | | 29 | 0.82 (0.51, 1.33) | 0.77 (0.47, 1.25) | 38 |
| MDS | 111 | | 58 | 1.21 (0.83, 1.75) | 1.25 (0.86, 1.81) | 53 |
| Outcome | Total | | Camp Lejeune # | Unadjusted  HR (95%CI) | Adjusted  HR (95% CI) | Camp Pendleton # |
| Lymphoid cancers | 180 | | 80 | 0.87 (0.65, 1.17) | 0.85 (0.63, 1.14) | 100 |
| Myeloid cancers | 342 | | 182 | 1.24 (1.00, 1.53) | 1.21 (0.97, 1.49) | 160 |
| Diabetes | 5,517 | | 2,631 | 1.00 (0.95, 1.05) | 0.97 (0.92, 1.02) | 2,886 |
| Anemias | 813 | | 404 | 1.08 (0.94, 1.24) | 1.02 (0.89, 1.17) | 409 |
| Cardiovascular disease | 29,549 | | 14,361 | 1.03 (1.01, 1.06) | 1.00 (0.98, 1.02) | 15,188 |
| Heart disease | 24,534 | | 11,933 | 1.03 (1.01, 1.06) | 1.01 (0.98, 1.03) | 12,601 |
| Stroke | 3,205 | | 1,595 | 1.08 (1.01, 1.16) | 1.03 (0.96, 1.10) | 1,610 |
| Circulatory diseases | 8,620 | | 4,081 | 0.98 (0.94, 1.03) | 0.94 (0.90, 0.98) | 4,539 |
| COPD | 4,688 | | 2,250 | 1.02 (0.96, 1.08) | 1.01 (0.96, 1.07) | 2,438 |
| Chronic Liver disease | 4,049 | | 1,758 | 0.83 (0.78, 0.89) | 0.87 (0.81, 0.92) | 2,291 |
| Cirrhosis | 4,735 | | 2,083 | 0.85 (0.80, 0.90) | 0.89 (0.84, 0.94) | 2,652 |
| Alcoholic Liver disease | 1,994 | | 821 | 0.76 (0.69, 0.83) | 0.80 (0.73, 0.88) | 1,173 |
| Nonalcoholic Liver disease | 2,653 | | 1,172 | 0.86 (0.80, 0.93) | 0.89 (0.82, 0.96) | 1,481 |
| Acute Kidney disease | 1,077 | | 530 | 1.06 (0.94, 1.19) | 1.03 (0.92, 1.17) | 547 |
| Chronic Kidney disease | 4,304 | | 2,060 | 1.01 (0.95, 1.07) | 0.94 (0.89, 1.00) | 2,244 |
| Parkinson disease | 224 | | 107 | 1.02 (0.79, 1.33) | 1.01 (0.78, 1.31) | 117 |
| ALS | 261 | | 121 | 0.95 (0.74, 1.21) | 0.96 (0.75, 1.23) | 140 |
| Multiple sclerosis | 148 | | 84 | 1.42 (1.03, 1.97) | 1.39 (1.00, 1.92) | 64 |
| Alcoholism | 3,706 | | 1,664 | 0.88 (0.83, 0.94) | 0.90 (0.85, 0.96) | 2,042 |
| Suicide | 6,911 | | 3,191 | 0.92 (0.87, 0.96) | 0.95 (0.91, 1.00) | 3,720 |

CL = 217,988 Males = 209,127 Females = 8,861

CP = 232,026 Males = 225,166 Females = 6,860

Total = 450,014 Males = 434,293 Females = 15,721

COPD: chronic obstructive pulmonary disease

ALS: amyotrophic lateral sclerosis

MDS: myelodysplastic syndrome

CML: chronic myeloid leukemia

AML: acute myeloid leukemia

CLL: chronic lymphocytic leukemia

ALL: acute lymphocytic leukema

CNS: central nervous system

HRs adjusted for sex, race, rank and education level; age was the time variable.

**Table S5**. Hazard ratios (HR) and 95% confidence intervals (CI) for the Marines/Navy personnel subgroup analysis of base location at Camp Lejeune (CL) vs. Camp Pendleton (CP); Contributing cause of death

| Outcome | Total | Camp Lejeune # | Unadjusted  HR (95% CI) | Adjusted  HR (95% CI) | Camp Pendleton # |
| --- | --- | --- | --- | --- | --- |
| All cancer malignancies | 7,908 | 3,903 | 1.05 (1.01, 1.10) | 1.06 (1.01, 1.10) | 4,005 |
| Oral cancers | 260 | 119 | 0.91 (0.71, 1.16) | 0.94 (0.74, 1.21) | 141 |
| Pharyngeal cancer | 142 | 65 | 0.91 (0.66, 1.27) | 0.94 (0.67, 1.31) | 77 |
| Esophageal cancer | 339 | 177 | 1.18 (0.95, 1.46) | 1.22 (0.98, 1.51) | 162 |
| Stomach cancers | 192 | 92 | 0.99 (0.75, 1.32) | 0.95 (0.72, 1.27) | 100 |
| Colorectal cancers | 744 | 368 | 1.05 (0.91, 1.22) | 1.03 (0.89, 1.19) | 376 |
| Colon cancer | 561 | 281 | 1.08 (0.92, 1.28) | 1.05 (0.89, 1.24) | 280 |
| Rectal cancer | 186 | 88 | 0.97 (0.72, 1.29) | 0.95 (0.71, 1.28) | 98 |
| Liver cancer | 617 | 293 | 0.98 (0.84, 1.15) | 1.03 (0.88, 1.21) | 324 |
| Pancreatic cancer | 539 | 277 | 1.14 (0.96, 1.35) | 1.14 (0.96, 1.35) | 262 |
| Laryngeal cancer | 87 | 44 | 1.11 (0.73, 1.69) | 1.14 (0.74, 1.73) | 43 |
| Lung cancer | 1,965 | 1,016 | 1.16 (1.06, 1.26) | 1.17 (1.07, 1.28) | 949 |
| Bone cancers | 42 | 16 | 0.66 (0.35, 1.23) | 0.67 (0.36, 1.26) | 26 |
| Soft tissue cancers | 114 | 58 | 1.11 (0.77, 1.61) | 1.09 (0.76, 1.58) | 56 |
| Melanoma | 223 | 110 | 1.04 (0.80, 1.36) | 1.09 (0.83, 1.41) | 113 |
| Female Breast cancer | 72 | 44 | 1.21 (0.76, 1.95) | 1.14 (0.70, 1.83) | 28 |
| Male Breast cancer | 19 | 7 | 0.63 (0.25, 1.60) | 0.59 (0.23, 1.51) | 12 |
| Cervical cancer | 14 | 9 | 1.19 (0.39, 3.65) | 1.25 (0.40, 3.85) | 5 |
| Uterine cancer | 12 | 5 | 0.62 (0.20, 1.97) | 0.62 (0.20, 1.98) | 7 |
| Ovarian cancer | 16 | 9 | 1.09 (0.41, 2.95) | 1.19 (0.44, 3.24) | 7 |
| Prostate cancer | 251 | 121 | 1.03 (0.81, 1.32) | 0.98 (0.76, 1.26) | 130 |
| Testicular cancer | 40 | 25 | 1.81 (0.95, 3.43) | 1.96 (1.03, 3.72) | 15 |
| Bladder cancer | 142 | 70 | 1.06 (0.76, 1.47) | 1.07 (0.77, 1.48) | 72 |
| Kidney cancer | 294 | 152 | 1.15 (0.92, 1.45) | 1.18 (0.94, 1.48) | 142 |
| Brain and CNS cancers | 410 | 187 | 0.90 (0.74, 1.09) | 0.91 (0.75, 1.11) | 223 |
| Thyroid cancer | 26 | 10 | 0.67 (0.30, 1.47) | 0.66 (0.30, 1.46) | 16 |
| Hematopoietic cancers | 867 | 422 | 1.02 (0.89, 1.16) | 1.01 (0.88, 1.15) | 445 |
| Hodgkin lymphoma | 90 | 44 | 1.02 (0.67, 1.54) | 1.02 (0.67, 1.54) | 46 |
| Non-Hodgkin lymphoma | 342 | 159 | 0.93 (0.75, 1.15) | 0.93 (0.75, 1.15) | 183 |
| Multiple myeloma | 138 | 69 | 1.08 (0.77, 1.51) | 1.04 (0.75, 1.46) | 69 |
| Leukemias | 317 | 163 | 1.14 (0.91, 1.41) | 1.12 (0.89, 1.39) | 154 |
| ALL | 47 | 22 | 0.93 (0.53, 1.66) | 0.92 (0.52, 1.63) | 25 |
| CLL | 20 | 7 | 0.58 (0.23, 1.45) | 0.56 (0.22, 1.41) | 13 |
| AML | 133 | 68 | 1.13 (0.81, 1.59) | 1.12 (0.80, 1.58) | 65 |
| CML | 35 | 16 | 0.89 (0.46, 1.74) | 0.83 (0.43, 1.63) | 19 |
| MDS | 29 | 19 | 2.04 (0.95, 4.40) | 2.25 (1.04, 4.86) | 10 |
| Outcome | Total | Camp Lejeune # | Unadjusted  HR (95%CI) | Adjusted  HR (95% CI) | Camp Pendleton # |
| Lymphoid cancers | 70 | 30 | 0.80 (0.50, 1.28) | 0.78 (0.48, 1.25) | 40 |
| Myeloid cancers | 172 | 89 | 1.15 (0.86, 1.56) | 1.13 (0.83, 1.52) | 83 |
| Diabetes | 2,506 | 1,155 | 0.92 (0.86, 1.00) | 0.93 (0.85, 1.00) | 1,351 |
| Anemias | 388 | 191 | 1.04 (0.86, 1.27) | 1.02 (0.83, 1.25) | 197 |
| Cardiovascular disease | 14,848 | 7,107 | 0.99 (0.96, 1.02) | 0.98 (0.95, 1.01) | 7,741 |
| Heart disease | 12,249 | 5,858 | 0.99 (0.95, 1.02) | 0.98 (0.95, 1.02) | 6,391 |
| Stroke | 1,543 | 757 | 1.04 (0.94, 1.15) | 1.00 (0.91, 1.11) | 786 |
| Circulatory diseases | 4,191 | 1,970 | 0.96 (0.90, 1.02) | 0.94 (0.88, 1.00) | 2,221 |
| COPD | 1,700 | 809 | 0.99 (0.90, 1.09) | 1.02 (0.93, 1.12) | 891 |
| Chronic Liver disease | 2,310 | 996 | 0.81 (0.75, 0.88) | 0.88 (0.81, 0.96) | 1,314 |
| Cirrhosis | 2,715 | 1,181 | 0.83 (0.77, 0.89) | 0.90 (0.83, 0.97) | 1,534 |
| Alcoholic Liver disease | 1,208 | 506 | 0.77 (0.69, 0.87) | 0.84 (0.75, 0.95) | 702 |
| Nonalcoholic Liver disease | 1,480 | 636 | 0.81 (0.73, 0.90) | 0.88 (0.79, 0.98) | 844 |
| Acute Kidney disease | 533 | 258 | 1.02 (0.86, 1.20) | 1.01 (0.85, 1.20) | 275 |
| Chronic Kidney disease | 2,003 | 936 | 0.95 (0.87, 1.04) | 0.91 (0.83, 1.00) | 1,067 |
| Parkinson disease | 44 | 24 | 1.31 (0.73, 2.38) | 1.33 (0.73, 2.41) | 20 |
| ALS | 136 | 66 | 1.01 (0.72, 1.42) | 1.02 (0.73, 1.43) | 70 |
| Multiple sclerosis | 80 | 44 | 1.31 (0.84, 2.03) | 1.29 (0.83, 2.01) | 36 |
| Alcoholism | 2,413 | 1,072 | 0.86 (0.79, 0.93) | 0.90 (0.83, 0.98) | 1,341 |
| Suicide | 5,118 | 2,375 | 0.92 (0.87, 0.97) | 0.96 (0.91, 1.02) | 2,743 |

CL = 159,128 Males = 151,026 Females = 8,102

CP = 168,406 Males = 162,473 Females = 5,933

Total = 327,534 Males = 313,499 Females = 14,035

COPD: chronic obstructive pulmonary disease

ALS: amyotrophic lateral sclerosis

MDS: myelodysplastic syndrome

CML: chronic myeloid leukemia

AML: acute myeloid leukemia

CLL: chronic lymphocytic leukemia

ALL: acute lymphocytic leukema

CNS: central nervous system

HRs adjusted for sex, race, rank and education level; age was the time variable.

**Table S6**. Hazard ratios (HR) and 95% lower and upper confidence intervals (CI) for the Marines/Navy personnel subgroup analysis of base duration between 1975 and 1985 at Camp Lejeune with Camp Pendleton as reference: Underlying cause of death

| Outcome | Low duration HR | Lower CI | Upper CI | Medium duration  HR | Lower CI | Upper CI | High duration  HR | Lower CI | Upper CL |
| --- | --- | --- | --- | --- | --- | --- | --- | --- | --- |
| All causes | 1.00 | 0.97 | 1.03 | 1.00 | 0.98 | 1.03 | 0.94 | 0.91 | 0.96 |
| All cancer malignancies | 1.10 | 1.03 | 1.17 | 1.07 | 1.00 | 1.14 | 1.04 | 0.98 | 1.11 |
| Oral cancers | 0.96 | 0.65 | 1.41 | 0.67 | 0.44 | 1.03 | 1.28 | 0.91 | 1.79 |
| Pharyngeal cancer | 0.88 | 0.51 | 1.53 | 0.74 | 0.42 | 1.30 | 1.21 | 0.75 | 1.95 |
| Esophageal cancer | 1.37 | 1.01 | 1.86 | 0.99 | 0.71 | 1.37 | 1.45 | 1.09 | 1.94 |
| Stomach cancers | 1.05 | 0.70 | 1.58 | 0.83 | 0.54 | 1.27 | 0.91 | 0.60 | 1.38 |
| Colorectal cancers | 1.02 | 0.82 | 1.26 | 1.08 | 0.88 | 1.33 | 0.97 | 0.78 | 1.20 |
| Colon cancer | 1.03 | 0.80 | 1.33 | 1.10 | 0.86 | 1.39 | 1.05 | 0.83 | 1.34 |
| Rectal cancer | 0.98 | 0.64 | 1.51 | 1.04 | 0.69 | 1.56 | 0.72 | 0.45 | 1.16 |
| Liver cancer | 1.17 | 0.93 | 1.48 | 1.04 | 0.82 | 1.32 | 0.99 | 0.77 | 1.27 |
| Pancreatic cancer | 1.11 | 0.86 | 1.43 | 1.12 | 0.88 | 1.43 | 1.15 | 0.90 | 1.46 |
| Laryngeal cancer | 1.35 | 0.73 | 2.49 | 0.85 | 0.42 | 1.71 | 0.97 | 0.50 | 1.86 |
| Lung cancer | 1.16 | 1.01 | 1.32 | 1.26 | 1.12 | 1.43 | 1.15 | 1.01 | 1.30 |
| Bone cancers | 0.89 | 0.36 | 2.19 | 1.28 | 0.59 | 2.76 | 0.14 | 0.02 | 1.03 |
| Soft tissue cancers | 1.13 | 0.64 | 1.98 | 1.31 | 0.79 | 2.18 | 0.80 | 0.44 | 1.46 |
| Melanoma | 0.87 | 0.57 | 1.34 | 1.18 | 0.82 | 1.71 | 1.04 | 0.72 | 1.52 |
| Female Breast cancer | 1.22 | 0.66 | 2.27 | 0.68 | 0.29 | 1.58 | 1.70 | 0.90 | 3.20 |
| Male Breast cancer | 0.34 | 0.04 | 2.63 | 0.58 | 0.13 | 2.61 | 0.24 | 0.03 | 1.90 |
| Cervical cancer | 2.62 | 0.76 | 9.05 | 0.59 | 0.07 | 5.14 | 0.45 | 0.05 | 3.88 |
| Uterine cancer | 1.06 | 0.26 | 4.26 | 0.87 | 0.18 | 4.25 | - | - | - |
| Ovarian cancer | 2.22 | 0.64 | 7.64 | 1.10 | 0.22 | 5.52 | 0.44 | 0.05 | 3.68 |
| Prostate cancer | 1.06 | 0.72 | 1.56 | 0.69 | 0.45 | 1.07 | 0.94 | 0.64 | 1.39 |
| Testicular cancer | 2.45 | 1.00 | 6.01 | 1.21 | 0.39 | 3.75 | 1.86 | 0.69 | 5.00 |
| Bladder cancer | 1.02 | 0.61 | 1.73 | 0.80 | 0.46 | 1.38 | 1.24 | 0.78 | 1.96 |
| Kidney cancer | 1.33 | 0.95 | 1.86 | 1.23 | 0.88 | 1.72 | 1.04 | 0.73 | 1.49 |
| Outcome | Low duration  HR | Lower CI | Upper CI | Medium duration  HR | Lower CI | Upper CI | High duration  HR | Lower CI | Upper CL |
| Brain and CNS cancers | 0.88 | 0.65 | 1.19 | 0.84 | 0.62 | 1.13 | 0.93 | 0.70 | 1.22 |
| Thyroid cancer | 0.84 | 0.23 | 2.98 | 1.06 | 0.34 | 3.28 | 0.29 | 0.04 | 2.25 |
| Hematopoietic cancers | 1.07 | 0.87 | 1.32 | 1.10 | 0.90 | 1.35 | 0.87 | 0.70 | 1.08 |
| Hodgkin lymphoma | 0.81 | 0.37 | 1.76 | 1.01 | 0.49 | 2.05 | 1.24 | 0.63 | 2.42 |
| Non-Hodgkin lymphoma | 1.05 | 0.75 | 1.47 | 0.98 | 0.70 | 1.37 | 0.72 | 0.49 | 1.05 |
| Multiple myeloma | 1.02 | 0.60 | 1.75 | 1.26 | 0.79 | 2.03 | 0.94 | 0.57 | 1.58 |
| Leukemias | 1.17 | 0.84 | 1.64 | 1.19 | 0.86 | 1.64 | 0.95 | 0.67 | 1.35 |
| ALL | 0.96 | 0.41 | 2.23 | 1.05 | 0.47 | 2.34 | 0.66 | 0.25 | 1.74 |
| CLL | 0.88 | 0.19 | 4.20 | 0.40 | 0.05 | 3.17 | 1.46 | 0.43 | 4.95 |
| AML | 1.18 | 0.72 | 1.94 | 0.83 | 0.48 | 1.44 | 1.13 | 0.69 | 1.84 |
| CML | 0.19 | 0.03 | 1.44 | 1.63 | 0.72 | 3.69 | 0.18 | 0.02 | 1.37 |
| MDS | 1.77 | 0.44 | 7.11 | 2.37 | 0.66 | 8.49 | 3.11 | 0.86 | 11.20 |
| Lymphoid cancers | 0.69 | 0.30 | 1.55 | 0.79 | 0.38 | 1.65 | 0.92 | 0.47 | 1.83 |
| Myeloid cancers | 1.10 | 0.69 | 1.74 | 1.03 | 0.65 | 1.61 | 1.15 | 0.75 | 1.76 |
| Diabetes | 0.89 | 0.72 | 1.09 | 1.11 | 0.93 | 1.34 | 0.86 | 0.71 | 1.06 |
| Anemias | 0.75 | 0.21 | 2.64 | 1.12 | 0.40 | 3.16 | 0.88 | 0.28 | 2.72 |
| Cardiovascular disease | 1.00 | 0.94 | 1.06 | 0.99 | 0.94 | 1.05 | 0.98 | 0.92 | 1.04 |
| Heart disease | 0.98 | 0.92 | 1.05 | 0.99 | 0.93 | 1.06 | 0.99 | 0.93 | 1.06 |
| Stroke | 1.01 | 0.83 | 1.23 | 1.06 | 0.88 | 1.27 | 1.05 | 0.87 | 1.28 |
| Circulatory diseases | 1.12 | 0.92 | 1.36 | 0.94 | 0.77 | 1.15 | 0.81 | 0.65 | 1.00 |
| COPD | 1.06 | 0.84 | 1.33 | 1.18 | 0.95 | 1.46 | 1.00 | 0.80 | 1.26 |
| Chronic Liver disease | 0.88 | 0.75 | 1.03 | 0.94 | 0.81 | 1.09 | 0.99 | 0.84 | 1.15 |
| Cirrhosis | 0.87 | 0.75 | 1.01 | 0.97 | 0.84 | 1.12 | 0.99 | 0.85 | 1.14 |
| Alcoholic Liver disease | 0.81 | 0.66 | 0.99 | 0.89 | 0.73 | 1.07 | 0.90 | 0.74 | 1.09 |
| Nonalcoholic Liver disease | 1.03 | 0.79 | 1.34 | 1.04 | 0.81 | 1.34 | 1.18 | 0.91 | 1.52 |
| Acute Kidney disease | 0.82 | 0.34 | 2.00 | 0.59 | 0.23 | 1.54 | 0.46 | 0.16 | 1.34 |
| Chronic Kidney disease | 1.00 | 0.71 | 1.42 | 1.06 | 0.77 | 1.46 | 0.72 | 0.49 | 1.04 |
| Parkinson disease | 2.07 | 0.62 | 6.95 | 2.63 | 0.91 | 7.66 | 1.59 | 0.51 | 4.96 |
| ALS | 0.85 | 0.49 | 1.50 | 1.23 | 0.77 | 1.96 | 1.13 | 0.71 | 1.80 |
| Outcome | Low duration  HR | Lower CI | Upper CI | Medium duration  HR | Lower CI | Upper CI | High duration  HR | Lower CI | Upper CL |
| Multiple sclerosis | 1.88 | 0.98 | 3.61 | 0.97 | 0.44 | 2.14 | 0.77 | 0.33 | 1.79 |
| Alcoholism | 0.96 | 0.75 | 1.22 | 0.91 | 0.71 | 1.16 | 0.85 | 0.66 | 1.10 |
| Suicide | 1.01 | 0.92 | 1.11 | 0.96 | 0.87 | 1.05 | 0.81 | 0.73 | 0.90 |

COPD: chronic obstructive pulmonary disease

ALS: amyotrophic lateral sclerosis

MDS: myelodysplastic syndrome

CML: chronic myeloid leukemia

AML: acute myeloid leukemia

CLL: chronic lymphocytic leukemia

ALL: acute lymphocytic leukema

CNS: central nervous system

HRs adjusted for sex, race, rank and education level; age was the time variable.

Low duration (1 – 2 quarters); Medium duration (>2 – 7 quarters); High duration (>7 quarters).

Number of quarters potentially exposed to the contaminated drinking water at Camp Lejeune, 1975 - 1985:

Mean = 6.1

Median = 5.0

Minimum = 1

Maximum = 38

Interquartile range = 7 (25^th^ percentile = 2; 75^th^ percentile = 9)

**Table S7**. Hazard ratios (HR) and 95% confidence intervals (CI) for the analysis of civilian employees at Camp Lejeune (CL) vs. Camp Pendleton (CP): Contributing cause of death

| Outcome | Total | Camp Lejeune # | Unadjusted  HR (95% CI) | Adjusted  HR (95% CI) | Camp Pendleton # |
| --- | --- | --- | --- | --- | --- |
| All cancers | 1,990 | 993 | 1.01 (0.92, 1.10) | 1.01 (0.92, 1.11) | 997 |
| All malignancies | 1,938 | 960 | 0.99 (0.90, 1.08) | 1.00 (0.91, 1.09) | 978 |
| Oral cancers | 29 | 15 | 1.05 (0.51, 2.19) | 1.12 (0.53, 2.38) | 14 |
| Pharynx | 19 | 12 | 1.69 (0.66, 4.30) | 1.83 (0.70, 4.78) | 7 |
| Esophagus | 43 | 16 | 0.60 (0.32, 1.11) | 0.68 (0.35, 1.30) | 27 |
| Stomach | 46 | 24 | 1.07 (0.60, 1.91) | 0.93 (0.50, 1.74) | 22 |
| Colorectal cancers | 153 | 72 | 0.91 (0.66, 1.24) | 0.92 (0.66, 1.29) | 81 |
| Colon | 121 | 57 | 0.91 (0.64, 1.30) | 0.92 (0.63, 1.35) | 64 |
| Rectum and  Rectosigmoid junction | 34 | 16 | 0.90 (0.46, 1.76) | 0.94 (0.47, 1.89) | 18 |
| Rectum only | 24 | 11 | 0.85 (0.38, 1.90) | 0.86 (0.38, 1.96) | 13 |
| Liver, biliary, gall bladder | 54 | 21 | 0.65 (0.38, 1.13) | 0.69 (0.39, 1.21) | 33 |
| Liver and bile ducts | 43 | 17 | 0.67 (0.36, 1.24) | 0.69 (0.37, 1.31) | 26 |
| Primary liver | 19 | 7 | 0.59 (0.23, 1.51) | 0.70 (0.27, 1.83) | 12 |
| Pancreas | 110 | 45 | 0.68 (0.47, 1.00) | 0.76 (0.52, 1.13) | 65 |
| Larynx | 19 | 13 | 2.11 (0.80, 5.56) | 1.69 (0.62, 4.62) | 6 |
| Lung | 639 | 333 | 1.09 (0.93, 1.27) | 1.11 (0.95, 1.31) | 306 |
| Urinary bladder | 57 | 22 | 0.69 (0.40, 1.17) | 0.62 (0.35, 1.10) | 35 |
| Kidney | 45 | 25 | 1.23 (0.68, 2.21) | 1.12 (0.60, 2.08) | 20 |
| Brain and CNS | 47 | 19 | 0.65 (0.36, 1.17) | 0.70 (0.39, 1.27) | 28 |
| Connective tissue | 12 | 5 | 0.69 (0.22, 2.17) | 0.52 (0.15, 1.78) | 7 |
| Melanoma | 18 | 12 | 2.03 (0.76, 5.41) | 2.50 (0.92, 6.77) | 6 |
| Hematopoietic cancers | 212 | 97 | 0.86 (0.66, 1.13) | 0.88 (0.67, 1.17) | 115 |
| Lymphoid cancers | 28 | 11 | 0.69 (0.32, 1.47) | 0.82 (0.37, 1.81) | 17 |
| Myeloid cancers | 43 | 22 | 1.03 (0.57, 1.88) | 1.08 (0.58, 2.02) | 21 |
| Hodgkin lymphoma | 7 | 4 | 1.26 (0.28, 5.66) | 1.23 (0.27, 5.68) | 3 |
| Non-Hodgkin lymphoma | 90 | 40 | 0.84 (0.55, 1.27) | 0.87 (0.57, 1.35) | 50 |
| Multiple myeloma | 35 | 15 | 0.73 (0.37, 1.43) | 0.73 (0.36, 1.48) | 20 |
| Leukemias | 84 | 40 | 0.92 (0.60, 1.42) | 1.00 (0.64, 1.57) | 44 |
| CLL | 24 | 10 | 0.78 (0.35, 1.76) | 0.82 (0.35, 1.90) | 14 |
| AML | 32 | 16 | 0.99 (0.49, 1.98) | 1.01 (0.49, 2.08) | 16 |
| CML | 11 | 7 | 1.69 (0.49, 5.78) | 1.73 (0.47, 6.42) | 4 |
| Female Breast | 95 | 58 | 1.37 (0.91, 2.08) | 1.33 (0.87, 2.03) | 37 |
| Uterus | 21 | 11 | 1.00 (0.42, 2.35) | 0.97 (0.40, 2.36) | 10 |
| Ovary | 37 | 14 | 0.55 (0.28, 1.08) | 0.56 (0.28, 1.11) | 23 |
| Prostate | 182 | 101 | 1.38 (1.03, 1.85) | 1.08 (0.79, 1.50) | 81 |
| Diabetes | 775 | 380 | 1.00 (0.87, 1.15) | 0.94 (0.81, 1.09) | 395 |
| Cardiovascular disease | 3,831 | 1,785 | 0.91 (0.86, 0.97) | 0.90 (0.84, 0.96) | 2,046 |
| Outcome | Total | Camp Lejeune # | Unadjusted  HR (95% CI) | Adjusted  HR (95% CI) | Camp Pendleton # |
| Anemias | 116 | 60 | 1.14 (0.79, 1.64) | 1.00 (0.67, 1.48) | 56 |
| Chronic liver disease | 150 | 59 | 0.60 (0.43, 0.83) | 0.66 (0.47, 0.92) | 91 |
| Alcoholic liver disease | 61 | 21 | 0.47 (0.28, 0.80) | 0.60 (0.35, 1.04) | 40 |
| Nonalcoholic liver disease | 97 | 40 | 0.66 (0.44, 0.99) | 0.70 (0.46, 1.06) | 57 |
| Alcoholism | 81 | 33 | 0.64 (0.41, 0.99) | 0.66 (0.41, 1.05) | 48 |
| Chronic kidney disease | 591 | 295 | 1.06 (0.90, 1.25) | 0.97 (0.82, 1.16) | 296 |
| COPD | 871 | 418 | 0.98 (0.85, 1.11) | 1.05 (0.92, 1.20) | 453 |
| Multiple sclerosis | 10 | 3 | 0.41 (0.11, 1.58) | 0.34 (0.09, 1.36) | 7 |
| Amyotrophic Lateral Sclerosis | 18 | 7 | 0.63 (0.24, 1.63) | 0.57 (0.21, 1.51) | 11 |
| Parkinson disease | 97 | 44 | 0.93 (0.62, 1.38) | 1.06 (0.70, 1.60) | 53 |
| Suicide | 107 | 52 | 0.86 (0.59, 1.25) | 0.97 (0.65, 1.43) | 55 |

HR: hazard ratio

CI: confidence interval

CNS: Central nervous system cancers

CLL: Chronic lymphcytic leukemia

AML: Acute myeloid leukemia

CML: Chronic myeloid leukemia

COPD: Chronic obstructive pulmonary disease

Totals:

Camp Lejeune = 7,332 Females = 3,624 Males = 3,708

Camp Pendleton = 6,677 Females = 3,031 Males = 3,646

Causes of death that were not evaluated because the number of cases were <2 for CL and/or CP:

Testicular cancer

Male breast cancer

Thyroid cancer

Acute lymphocytic leukemia

HRs adjusted for sex, race, blue collar work (y/n) and education level; age was the time variable.

**Table S8**. Hazard ratios (HR) and 95% lower and upper confidence intervals (CI) for the analysis of civilian employees’ employment duration at Camp Lejeune between October 1972 and December 1985 with Camp Pendleton as reference: Underlying cause of death

| Outcome | Low duration  HR | Lower CI | Upper CI | Medium duration  HR | Lower CI | Upper CI | High duration  HR | Lower CI | Upper CL |
| --- | --- | --- | --- | --- | --- | --- | --- | --- | --- |
| All causes | 0.99 | 0.90 | 1.08 | 1.00 | 0.93 | 1.08 | 0.93 | 0.87 | 0.99 |
| All cancers | 1.05 | 0.90 | 1.23 | 1.02 | 0.89 | 1.17 | 0.97 | 0.86 | 1.10 |
| all malignancies | 1.05 | 0.90 | 1.23 | 1.02 | 0.89 | 1.17 | 0.96 | 0.85 | 1.09 |
| Oral cancers | 1.13 | 0.24 | 5.28 | 1.35 | 0.41 | 4.40 | 0.83 | 0.25 | 2.75 |
| Pharynx | 1.45 | 0.16 | 13.23 | 3.80 | 0.88 | 16.3 | 1.54 | 0.31 | 7.56 |
| Esophagus | 0.97 | 0.33 | 2.83 | 0.34 | 0.08 | 1.45 | 0.66 | 0.27 | 1.60 |
| Stomach | 0.61 | 0.18 | 2.09 | 0.94 | 0.37 | 2.39 | 1.23 | 0.57 | 2.66 |
| Colorectal cancers | 1.23 | 0.72 | 2.13 | 0.78 | 0.45 | 1.36 | 0.73 | 0.45 | 1.19 |
| Colon | 1.31 | 0.73 | 2.35 | 0.73 | 0.39 | 1.39 | 0.69 | 0.39 | 1.20 |
| Rectum and  Rectosigmoid junction | 0.81 | 0.18 | 3.65 | 0.91 | 0.29 | 2.80 | 0.98 | 0.38 | 2.54 |
| Rectum only | 1.24 | 0.26 | 5.92 | 0.32 | 0.04 | 2.53 | 1.04 | 0.33 | 3.26 |
| Liver, biliary, gall bladder | 0.87 | 0.33 | 2.30 | 0.94 | 0.42 | 2.10 | 0.55 | 0.23 | 1.29 |
| Liver and bile ducts | 1.22 | 0.45 | 3.30 | 0.78 | 0.29 | 2.10 | 0.58 | 0.23 | 1.49 |
| Primary liver | 0.56 | 0.07 | 4.55 | 0.44 | 0.05 | 3.57 | 1.32 | 0.39 | 4.42 |
| Pancreas | 0.76 | 0.40 | 1.47 | 0.76 | 0.42 | 1.37 | 0.64 | 0.36 | 1.13 |
| Larynx | 1.82 | 0.34 | 9.74 | 1.05 | 0.20 | 5.46 | 1.22 | 0.31 | 4.78 |
| Lung | 1.12 | 0.85 | 1.49 | 1.19 | 0.94 | 1.50 | 1.11 | 0.90 | 1.37 |
| Urinary bladder | 1.76 | 0.70 | 4.40 | 0.47 | 0.16 | 1.37 | 0.45 | 0.19 | 1.06 |
| Kidney | 1.36 | 0.48 | 3.82 | 1.36 | 0.54 | 3.41 | 1.68 | 0.75 | 3.76 |
| Brain and CNS | 0.31 | 0.07 | 1.34 | 1.00 | 0.47 | 2.15 | 0.51 | 0.21 | 1.24 |
| Connective tissue | 0.00 | - | - | 0.93 | 0.18 | 4.82 | 0.98 | 0.22 | 4.39 |
| Melanoma | 3.77 | 0.87 | 16.2 | 2.25 | 0.53 | 9.61 | 2.73 | 0.81 | 9.24 |
| Hematopoietic cancers | 0.80 | 0.46 | 1.40 | 0.97 | 0.61 | 1.53 | 1.13 | 0.77 | 1.66 |
| Outcome | Low duration  HR | Lower CI | Upper CI | Medium duration  HR | Lower CI | Upper CI | High duration  HR | Lower CI | Upper CL |
| Lymphoid cancers | 0.49 | 0.06 | 3.92 | 0.91 | 0.19 | 4.32 | 0.97 | 0.26 | 3.67 |
| Myeloid cancers | 0.88 | 0.30 | 2.60 | 0.67 | 0.22 | 1.99 | 1.28 | 0.59 | 2.79 |
| Hodgkin lymphoma | 0.00 | - | - | 1.42 | 0.12 | 16.2 | 2.52 | 0.35 | 18.0 |
| Non-Hodgkin lymphoma | 0.67 | 0.26 | 1.72 | 1.08 | 0.55 | 2.10 | 1.06 | 0.59 | 1.92 |
| Multiple myeloma | 1.61 | 0.56 | 4.63 | 0.69 | 0.19 | 2.45 | 0.93 | 0.35 | 2.48 |
| Leukemias | 0.67 | 0.26 | 1.72 | 0.94 | 0.45 | 2.00 | 1.23 | 0.67 | 2.26 |
| CLL | 0.00 | - | - | 1.06 | 0.22 | 5.15 | 1.04 | 0.27 | 4.01 |
| AML | 0.55 | 0.12 | 2.42 | 0.79 | 0.26 | 2.42 | 1.32 | 0.56 | 3.12 |
| CML | 1.32 | 0.14 | 12.27 | 0.00 | - | - | 1.81 | 0.34 | 9.77 |
| Female Breast | 1.12 | 0.62 | 2.04 | 1.53 | 0.87 | 2.68 | 0.92 | 0.45 | 1.89 |
| Uterus | 0.90 | 0.24 | 3.40 | 1.18 | 0.35 | 3.98 | 0.89 | 0.23 | 3.43 |
| Ovary | 0.48 | 0.16 | 1.43 | 0.77 | 0.30 | 1.94 | 0.56 | 0.19 | 1.67 |
| Prostate | 1.51 | 0.83 | 2.74 | 0.98 | 0.58 | 1.69 | 0.90 | 0.57 | 1.42 |
| Diabetes | 1.13 | 0.72 | 1.76 | 0.55 | 0.33 | 0.90 | 0.76 | 0.53 | 1.11 |
| Cardiovascular disease | 0.90 | 0.78 | 1.05 | 0.98 | 0.87 | 1.10 | 0.87 | 0.78 | 0.96 |
| Anemias | 3.97 | 0.73 | 21.7 | 0.78 | 0.08 | 7.64 | 1.41 | 0.26 | 7.60 |
| Chronic liver disease | 0.79 | 0.41 | 1.54 | 0.88 | 0.47 | 1.65 | 0.60 | 0.31 | 1.15 |
| Alcoholic liver disease | 0.72 | 0.30 | 1.74 | 0.57 | 0.22 | 1.47 | 0.40 | 0.15 | 1.04 |
| Nonalcoholic liver disease | 1.05 | 0.38 | 2.93 | 1.51 | 0.64 | 3.54 | 0.85 | 0.33 | 2.22 |
| Alcoholism | 1.03 | 0.27 | 3.86 | 0.30 | 0.04 | 2.42 | 0.59 | 0.15 | 2.29 |
| Chronic kidney disease | 2.17 | 1.00 | 4.71 | 1.92 | 1.01 | 3.67 | 1.60 | 0.88 | 2.91 |
| COPD | 1.25 | 0.89 | 1.76 | 0.95 | 0.71 | 1.28 | 0.75 | 0.57 | 0.99 |
| Multiple sclerosis | 0.90 | 0.09 | 9.21 | 0.83 | 0.08 | 8.19 | 0.79 | 0.08 | 7.80 |
| Amyotrophic Lateral Sclerosis | 0.00 | - | - | 0.53 | 0.11 | 2.48 | 0.61 | 0.16 | 2.34 |
| Parkinson disease | 0.23 | 0.03 | 1.69 | 1.19 | 0.55 | 2.54 | 1.60 | 0.88 | 2.90 |
| Suicide | 0.77 | 0.37 | 1.60 | 0.51 | 0.23 | 1.14 | 0.76 | 0.40 | 1.43 |

CNS: Central nervous system cancers

CLL: Chronic lymphcytic leukemia

AML: Acute myeloid leukemia

CML: Chronic myeloid leukemia

COPD: Chronic obstructive pulmonary disease

HRs adjusted for sex, race, blue collar work (y/n) and education level; age was the time variable.

Low duration: 1 – 5 quarters; Medium duration: 6 – 22 quarters; High duration: 23 – 53 quarters

Number of quarters potentially exposed to the contaminated drinking water at Camp Lejeune, October 1972 – December 1985:

Mean = 18.9

Median = 12.0

Minimum = 1

Maximum = 53

Interquartile range = 30 (25^th^ percentile = 3; 75^th^ percentile = 33)

**Table S9**. Chronic obstructive pulmonary disease (COPD) hazard ratio = 1.08. Adjusted for smoking prevalence (p) differences between the Camp Lejeune and Camp Pendleton Marines/Navy personnel subgroup.

| **Multidimensional RR CL-COPD Relationship Adjusted for smoking** | | | | | | | | | | | | | | |
| --- | --- | --- | --- | --- | --- | --- | --- | --- | --- | --- | --- | --- | --- | --- |
|  |  |  |  |  |  |  |  |  |  |  |  |  |  |  |
| **p(smoking+\|CL+)*** | **p(smoking+\|CL-)^#^** | **RR(smoking-COPD)** | **3** |  | **3.25** | **3.5** | **3.75** | **4** | **4.25** | **4.5** | **4.75** | **5** | **5.25** | **5.5** |
| **0.45** | **0.44** |  | 1.07 |  | 1.07 | 1.07 | 1.07 | 1.07 | 1.07 | 1.07 | 1.07 | 1.07 | 1.07 | 1.07 |
| **0.47** | **0.45** |  | 1.06 |  | 1.06 | 1.06 | 1.06 | 1.06 | 1.05 | 1.05 | 1.05 | 1.05 | 1.05 | 1.05 |
| **0.49** | **0.46** |  | 1.05 |  | 1.05 | 1.05 | 1.04 | 1.04 | 1.04 | 1.04 | 1.04 | 1.04 | 1.04 | 1.04 |
| **0.51** | **0.47** |  | 1.04 |  | 1.04 | 1.04 | 1.03 | 1.03 | 1.03 | 1.03 | 1.03 | 1.03 | 1.02 | 1.02 |
| **0.53** | **0.48** |  | 1.03 |  | 1.03 | 1.02 | 1.02 | 1.02 | 1.02 | 1.02 | 1.01 | 1.01 | 1.01 | 1.01 |
| **0.55** | **0.49** |  | **1.02** |  | **1.02** | **1.01** | **1.01** | **1.01** | **1.01** | **1.00** | **1.00** | **1.00** | **1.00** | **1.00** |
| **0.57** | **0.5** |  | 1.01 |  | 1.01 | 1.00 | 1.00 | 1.00 | 1.00 | 0.99 | 0.99 | 0.99 | 0.99 | 0.99 |
| **0.59** | **0.51** |  | 1.00 |  | 1.00 | 1.00 | 0.99 | 0.99 | 0.99 | 0.98 | 0.98 | 0.98 | 0.98 | 0.98 |
| **0.61** | **0.52** |  | 0.99 |  | 0.99 | 0.99 | 0.98 | 0.98 | 0.98 | 0.97 | 0.97 | 0.97 | 0.97 | 0.97 |
| **0.63** | **0.53** |  | 0.99 |  | 0.98 | 0.98 | 0.97 | 0.97 | 0.97 | 0.96 | 0.96 | 0.96 | 0.96 | 0.96 |
| **0.65** | **0.54** |  | 0.98 |  | 0.97 | 0.97 | 0.97 | 0.96 | 0.96 | 0.96 | 0.95 | 0.95 | 0.95 | 0.95 |

* Prevalence of smoking at Camp Lejeune

# Prevalence of smoking at Camp Pendleton

Assuming RRs for smoking and COPD range from 3.0 to 5.5 [3], for smoking to fully account for the COPD HR of 1.08, the smoking prevalence difference between Camp Lejeune and Camp Pendleton would be 6%, corresponding to 55% prevalence at Camp Lejeune and 49% prevalence at Camp Pendleton (i.e., the **bolded** prevalence difference resulting in the HR=1.00).

CL – Camp Lejeune

CP – Camp Pendleton

HR – hazard ratio

RR – risk ratio

COPD – Chronic obstructive pulmonary disease

**Table S10**. Kidney cancer hazard ratio = 1.21. Adjusted for a 6% smoking prevalence (p) difference between the Camp Lejeune and Camp Pendleton Marines/Navy personnel subgroup.

| **Multidimensional RR CL-Kidney cancer Relationship Adjusted for smoking** | | | | | | | | | | | | | | |
| --- | --- | --- | --- | --- | --- | --- | --- | --- | --- | --- | --- | --- | --- | --- |
|  |  |  |  |  |  |  |  |  |  |  |  |  |  |  |
| **p(smoking+\|CL+)*** | **p(smoking+\|CL-)^#^** | **RR(smoking-Kidney cancer)** | **1.25** |  | **1.3** | **1.35** | **1.4** | **1.45** | **1.5** | **1.55** | **1.6** | **1.65** | **1.7** | **1.75** |
| **0.45** | **0.44** |  | 1.20 |  | 1.20 | 1.20 | 1.20 | 1.20 | 1.20 | 1.20 | 1.20 | 1.20 | 1.20 | 1.20 |
| **0.47** | **0.45** |  | 1.20 |  | 1.20 | 1.20 | 1.20 | 1.20 | 1.20 | 1.20 | 1.19 | 1.19 | 1.19 | 1.19 |
| **0.49** | **0.46** |  | 1.20 |  | 1.20 | 1.19 | 1.19 | 1.19 | 1.19 | 1.19 | 1.19 | 1.19 | 1.19 | 1.19 |
| **0.51** | **0.47** |  | 1.20 |  | 1.19 | 1.19 | 1.19 | 1.19 | 1.19 | 1.19 | 1.18 | 1.18 | 1.18 | 1.18 |
| **0.53** | **0.48** |  | 1.19 |  | 1.19 | 1.19 | 1.19 | 1.18 | 1.18 | 1.18 | 1.18 | 1.18 | 1.17 | 1.17 |
| **0.55** | **0.49** |  | **1.19** |  | **1.19** | **1.18** | **1.18** | **1.18** | **1.18** | **1.18** | **1.17** | **1.17** | **1.17** | **1.17** |
| **0.57** | **0.5** |  | 1.19 |  | 1.18 | 1.18 | 1.18 | 1.18 | 1.17 | 1.17 | 1.17 | 1.17 | 1.16 | 1.16 |
| **0.59** | **0.51** |  | 1.18 |  | 1.18 | 1.18 | 1.17 | 1.17 | 1.17 | 1.17 | 1.16 | 1.16 | 1.16 | 1.16 |
| **0.61** | **0.52** |  | 1.18 |  | 1.18 | 1.17 | 1.17 | 1.17 | 1.16 | 1.16 | 1.16 | 1.16 | 1.15 | 1.15 |
| **0.63** | **0.53** |  | 1.18 |  | 1.18 | 1.17 | 1.17 | 1.16 | 1.16 | 1.16 | 1.15 | 1.15 | 1.15 | 1.14 |
| **0.65** | **0.54** |  | 1.18 |  | 1.17 | 1.17 | 1.16 | 1.16 | 1.16 | 1.15 | 1.15 | 1.15 | 1.14 | 1.14 |

* Prevalence of smoking at Camp Lejeune

# Prevalence of smoking at Camp Pendleton

RRs for smoking and kidney cancer were assumed to be between 1.25 and 1.75 [4-5].

The reduction in the HR for kidney cancer by adjusting for a 6% difference in smoking prevalence between Camp Lejeune and Camp Pendleton would be from a HR of 1.21 to HRs between 1.17 and 1.19 (**bolded**).

CL – Camp Lejeune

CP – Camp Pendleton

HR – hazard ratio

RR – risk ratio

**Table S11**. Esophageal cancer hazard ratio = 1.24. Adjusted for a 6% smoking prevalence (p) difference between the Camp Lejeune and Camp Pendleton Marines/Navy personnel subgroup.

| **Multidimensional RR CL-esophageal cancer Relationship Adjusted for smoking** | | | | | | | | | | | | | | |
| --- | --- | --- | --- | --- | --- | --- | --- | --- | --- | --- | --- | --- | --- | --- |
|  |  |  |  |  |  |  |  |  |  |  |  |  |  |  |
| **p(smoking+\|CL+)*** | **p(smoking+\|CL-)^#^** | **RR(smoking-esophageal cancer)** | **1.5** |  | **1.75** | **2** | **2.25** | **2.5** | **2.75** | **3** | **3.25** | **3.5** | **4** | **4.5** |
| **0.45** | **0.44** |  | 1.23 |  | 1.23 | 1.23 | 1.23 | 1.23 | 1.23 | 1.23 | 1.23 | 1.22 | 1.22 | 1.22 |
| **0.47** | **0.45** |  | 1.23 |  | 1.23 | 1.22 | 1.22 | 1.22 | 1.22 | 1.21 | 1.21 | 1.21 | 1.21 | 1.21 |
| **0.49** | **0.46** |  | 1.22 |  | 1.22 | 1.21 | 1.21 | 1.21 | 1.20 | 1.20 | 1.20 | 1.20 | 1.19 | 1.19 |
| **0.51** | **0.47** |  | 1.22 |  | 1.21 | 1.21 | 1.20 | 1.20 | 1.19 | 1.19 | 1.19 | 1.19 | 1.18 | 1.18 |
| **0.53** | **0.48** |  | 1.22 |  | 1.21 | 1.20 | 1.19 | 1.19 | 1.18 | 1.18 | 1.18 | 1.17 | 1.17 | 1.16 |
| **0.55** | **0.49** |  | **1.21** |  | **1.20** | **1.19** | **1.18** | **1.18** | **1.17** | **1.17** | **1.16** | **1.16** | **1.16** | **1.15** |
| **0.57** | **0.5** |  | 1.21 |  | 1.19 | 1.18 | 1.18 | 1.17 | 1.16 | 1.16 | 1.15 | 1.15 | 1.14 | 1.14 |
| **0.59** | **0.51** |  | 1.20 |  | 1.19 | 1.18 | 1.17 | 1.16 | 1.15 | 1.15 | 1.14 | 1.14 | 1.13 | 1.13 |
| **0.61** | **0.52** |  | 1.20 |  | 1.18 | 1.17 | 1.16 | 1.15 | 1.15 | 1.14 | 1.13 | 1.13 | 1.12 | 1.11 |
| **0.63** | **0.53** |  | 1.19 |  | 1.18 | 1.16 | 1.15 | 1.14 | 1.14 | 1.13 | 1.12 | 1.12 | 1.11 | 1.10 |
| **0.65** | **0.54** |  | 1.19 |  | 1.17 | 1.16 | 1.15 | 1.14 | 1.13 | 1.12 | 1.11 | 1.11 | 1.10 | 1.09 |

* Prevalence of smoking at Camp Lejeune

# Prevalence of smoking at Camp Pendleton

The RRs for smoking and esophageal cancer were assumed to be between 1.5 and 4.5 [4,6].

The reduction in the HR for esophageal cancer by adjusting for a 6% difference in smoking prevalence between Camp Lejeune and Camp Pendleton would be from a HR of 1.24 to HRs between 1.15 and 1.21 (**bolded**).

CL – Camp Lejeune

CP – Camp Pendleton

HR – hazard ratio

RR – risk ratio

**Table S12**. Lung cancer hazard ratio = 1.18. Adjusted for a 6% smoking prevalence (p) difference between the Camp Lejeune and Camp Pendleton Marines/Navy personnel subgroup.

| **Multidimensional RR CL-Lung cancer Relationship Adjusted for smoking** | | | | | | | | | | | | | | |
| --- | --- | --- | --- | --- | --- | --- | --- | --- | --- | --- | --- | --- | --- | --- |
|  |  |  |  |  |  |  |  |  |  |  |  |  |  |  |
| **p(smoking+\|CL+)*** | **p(smoking+\|CL-)^#^** | **RR(smoking-Lung cancer)** | **7** |  | **7.5** | **8** | **8.5** | **9** | **9.5** | **10** | **10.5** | **11** | **11.5** | **12** |
| **0.45** | **0.44** |  | 1.16 |  | 1.16 | 1.16 | 1.16 | 1.16 | 1.15 | 1.15 | 1.15 | 1.15 | 1.15 | 1.15 |
| **0.47** | **0.45** |  | 1.14 |  | 1.14 | 1.14 | 1.14 | 1.14 | 1.14 | 1.14 | 1.13 | 1.13 | 1.13 | 1.13 |
| **0.49** | **0.46** |  | 1.12 |  | 1.12 | 1.12 | 1.12 | 1.12 | 1.12 | 1.12 | 1.12 | 1.12 | 1.12 | 1.11 |
| **0.51** | **0.47** |  | 1.11 |  | 1.10 | 1.10 | 1.10 | 1.10 | 1.10 | 1.10 | 1.10 | 1.10 | 1.10 | 1.10 |
| **0.53** | **0.48** |  | 1.09 |  | 1.09 | 1.09 | 1.09 | 1.09 | 1.08 | 1.08 | 1.08 | 1.08 | 1.08 | 1.08 |
| **0.55** | **0.49** |  | **1.08** |  | **1.08** | **1.07** | **1.07** | **1.07** | **1.07** | **1.07** | **1.07** | **1.07** | **1.07** | **1.07** |
| **0.57** | **0.5** |  | 1.06 |  | 1.06 | 1.06 | 1.06 | 1.06 | 1.06 | 1.05 | 1.05 | 1.05 | 1.05 | 1.05 |
| **0.59** | **0.51** |  | 1.05 |  | 1.05 | 1.05 | 1.05 | 1.04 | 1.04 | 1.04 | 1.04 | 1.04 | 1.04 | 1.04 |
| **0.61** | **0.52** |  | 1.04 |  | 1.04 | 1.04 | 1.03 | 1.03 | 1.03 | 1.03 | 1.03 | 1.03 | 1.03 | 1.02 |
| **0.63** | **0.53** |  | 1.03 |  | 1.03 | 1.02 | 1.02 | 1.02 | 1.02 | 1.02 | 1.02 | 1.01 | 1.01 | 1.01 |
| **0.65** | **0.54** |  | 1.02 |  | 1.01 | 1.01 | 1.01 | 1.01 | 1.01 | 1.01 | 1.00 | 1.00 | 1.00 | 1.00 |

* Prevalence of smoking at Camp Lejeune

# Prevalence of smoking at Camp Pendleton

RR for smoking and lung cancer was assumed to range between 7.00 and 12.00 [4].

The reduction in the HR for lung cancer by adjusting for a 6% difference in smoking prevalence between Camp Lejeune and Camp Pendleton would be from a HR of 1.18 to HRs between 1.07 and 1.08 (**bolded**).

CL – Camp Lejeune

CP – Camp Pendleton

HR – hazard ratio

RR – risk ratio

**Table S13**. Parkinson disease hazard ratio = 2.05. Adjusted for a 6% smoking prevalence (p) difference between the Camp Lejeune and Camp Pendleton Marines/Navy personnel subgroup.

| **Multidimensional RR CL-Parkinson Relationship Adjusted for smoking** | | | | | | | | | | | | | | |
| --- | --- | --- | --- | --- | --- | --- | --- | --- | --- | --- | --- | --- | --- | --- |
|  |  |  |  |  |  |  |  |  |  |  |  |  |  |  |
| **p(smoking+\|CL+)*** | **p(smoking+\|CL-)^#^** | **RR(smoking-Parkinson)** | **0.3** |  | **0.35** | **0.4** | **0.45** | **0.5** | **0.55** | **0.6** | **0.65** | **0.7** | **0.8** | **0.85** |
| **0.45** | **0.44** |  | 2.07 |  | 2.07 | 2.07 | 2.07 | 2.07 | 2.07 | 2.06 | 2.06 | 2.06 | 2.06 | 2.06 |
| **0.47** | **0.45** |  | 2.10 |  | 2.09 | 2.09 | 2.08 | 2.08 | 2.08 | 2.07 | 2.07 | 2.07 | 2.06 | 2.06 |
| **0.49** | **0.46** |  | 2.12 |  | 2.11 | 2.11 | 2.10 | 2.09 | 2.09 | 2.08 | 2.08 | 2.08 | 2.07 | 2.06 |
| **0.51** | **0.47** |  | 2.14 |  | 2.13 | 2.12 | 2.12 | 2.11 | 2.10 | 2.09 | 2.09 | 2.08 | 2.07 | 2.07 |
| **0.53** | **0.48** |  | 2.17 |  | 2.16 | 2.14 | 2.13 | 2.12 | 2.11 | 2.11 | 2.10 | 2.09 | 2.08 | 2.07 |
| **0.55** | **0.49** |  | **2.19** |  | **2.18** | **2.16** | **2.15** | **2.14** | **2.13** | **2.12** | **2.11** | **2.10** | **2.08** | **2.07** |
| **0.57** | **0.5** |  | 2.22 |  | 2.20 | 2.18 | 2.17 | 2.15 | 2.14 | 2.13 | 2.12 | 2.11 | 2.09 | 2.08 |
| **0.59** | **0.51** |  | 2.25 |  | 2.23 | 2.21 | 2.19 | 2.17 | 2.15 | 2.14 | 2.13 | 2.11 | 2.09 | 2.08 |
| **0.61** | **0.52** |  | 2.28 |  | 2.25 | 2.23 | 2.21 | 2.19 | 2.17 | 2.15 | 2.14 | 2.12 | 2.10 | 2.08 |
| **0.63** | **0.53** |  | 2.31 |  | 2.28 | 2.25 | 2.23 | 2.20 | 2.18 | 2.16 | 2.15 | 2.13 | 2.10 | 2.09 |
| **0.65** | **0.54** |  | 2.34 |  | 2.31 | 2.28 | 2.25 | 2.22 | 2.20 | 2.18 | 2.16 | 2.14 | 2.11 | 2.09 |

* Prevalence of smoking at Camp Lejeune

# Prevalence of smoking at Camp Pendleton

RRs for smoking and Parkinson disease range from 0.23 to 0.53 for current smokers to 0.80 (ex-smoker) [7-8].

The increase in the HR for Parkinson disease by adjusting for a 6% difference in smoking prevalence between Camp Lejeune and Camp Pendleton would be from a HR of 2.05 to HRs between 2.07 and 2.19 (**bolded**).

CL – Camp Lejeune

CP – Camp Pendleton

HR – hazard ratio

RR – risk ratio

**Table S14**. Chronic obstructive pulmonary disease (COPD) hazard ratio = 1.05. Adjusted for smoking prevalence (p) differences between the Camp Lejeune and Camp Pendleton civilian employees.

| **Multidimensional RR CL-COPD Relationship Adjusted for smoking** | | | | | | | | | | | | | | |
| --- | --- | --- | --- | --- | --- | --- | --- | --- | --- | --- | --- | --- | --- | --- |
|  |  |  |  |  |  |  |  |  |  |  |  |  |  |  |
| **p(smoking+\|CL+)*** | **p(smoking+\|CL-)^#^** | **RR(smoking-COPD)** | **3** |  | **3.25** | **3.5** | **3.75** | **4** | **4.25** | **4.5** | **4.75** | **5** | **5.25** | **5.5** |
| **0.45** | **0.44** |  | 1.04 |  | 1.04 | 1.04 | 1.04 | 1.04 | 1.04 | 1.04 | 1.04 | 1.04 | 1.04 | 1.04 |
| **0.47** | **0.45** |  | 1.03 |  | 1.03 | 1.03 | 1.03 | 1.03 | 1.02 | 1.02 | 1.02 | 1.02 | 1.02 | 1.02 |
| **0.49** | **0.46** |  | 1.02 |  | 1.02 | 1.02 | 1.01 | 1.01 | 1.01 | 1.01 | 1.01 | 1.01 | 1.01 | 1.01 |
| **0.51** | **0.47** |  | **1.01** |  | **1.01** | **1.01** | **1.00** | **1.00** | **1.00** | **1.00** | **1.00** | **1.00** | **1.00** | **0.99** |
| **0.53** | **0.48** |  | 1.00 |  | 1.00 | 1.00 | 0.99 | 0.99 | 0.99 | 0.99 | 0.99 | 0.98 | 0.98 | 0.98 |
| **0.55** | **0.49** |  | 0.99 |  | 0.99 | 0.99 | 0.98 | 0.98 | 0.98 | 0.98 | 0.97 | 0.97 | 0.97 | 0.97 |
| **0.57** | **0.5** |  | 0.98 |  | 0.98 | 0.98 | 0.97 | 0.97 | 0.97 | 0.97 | 0.96 | 0.96 | 0.96 | 0.96 |
| **0.59** | **0.51** |  | 0.97 |  | 0.97 | 0.97 | 0.96 | 0.96 | 0.96 | 0.96 | 0.95 | 0.95 | 0.95 | 0.95 |
| **0.61** | **0.52** |  | 0.97 |  | 0.96 | 0.96 | 0.95 | 0.95 | 0.95 | 0.95 | 0.94 | 0.94 | 0.94 | 0.94 |
| **0.63** | **0.53** |  | 0.96 |  | 0.95 | 0.95 | 0.95 | 0.94 | 0.94 | 0.94 | 0.93 | 0.93 | 0.93 | 0.93 |
| **0.65** | **0.54** |  | 0.95 |  | 0.95 | 0.94 | 0.94 | 0.93 | 0.93 | 0.93 | 0.93 | 0.92 | 0.92 | 0.92 |

* Prevalence of smoking at Camp Lejeune

# Prevalence of smoking at Camp Pendleton

Assuming RRs for smoking and COPD range from 3.0 to 5.5 [3], for smoking to fully account for the COPD HR of 1.05, the smoking prevalence difference between Camp Lejeune and Camp Pendleton would be 4%, corresponding to 51% prevalence at Camp Lejeune and 47% prevalence at Camp Pendleton (i.e., the **bolded** prevalence difference resulting in the HR=1.00).

CL – Camp Lejeune

CP – Camp Pendleton

HR – hazard ratio

RR – risk ratio

COPD – Chronic obstructive pulmonary disease

**Table S15**. Lung cancer hazard ratio = 1.13. Adjusted for a 4% smoking prevalence (p) difference between the Camp Lejeune and Camp Pendleton civilian employees.

| **Multidimensional RR CL-lung cancer Relationship Adjusted for smoking** | | | | | | | | | | | | | | |
| --- | --- | --- | --- | --- | --- | --- | --- | --- | --- | --- | --- | --- | --- | --- |
|  |  |  |  |  |  |  |  |  |  |  |  |  |  |  |
| **p(smoking+\|CL+)*** | **p(smoking+\|CL-)^#^** | **RR(smoking-lung cancer)** | **7** |  | **7.5** | **8** | **8.5** | **9** | **9.5** | **10** | **10.5** | **11** | **11.5** | **12** |
| **0.45** | **0.44** |  | 1.11 |  | 1.11 | 1.11 | 1.11 | 1.11 | 1.11 | 1.11 | 1.11 | 1.11 | 1.11 | 1.11 |
| **0.47** | **0.45** |  | 1.09 |  | 1.09 | 1.09 | 1.09 | 1.09 | 1.09 | 1.09 | 1.09 | 1.09 | 1.09 | 1.09 |
| **0.49** | **0.46** |  | 1.08 |  | 1.08 | 1.08 | 1.07 | 1.07 | 1.07 | 1.07 | 1.07 | 1.07 | 1.07 | 1.07 |
| **0.51** | **0.47** |  | **1.06** |  | **1.06** | **1.06** | **1.06** | **1.06** | **1.06** | **1.06** | **1.06** | **1.06** | **1.05** | **1.05** |
| **0.53** | **0.48** |  | 1.05 |  | 1.05 | 1.05 | 1.04 | 1.04 | 1.04 | 1.04 | 1.04 | 1.04 | 1.04 | 1.04 |
| **0.55** | **0.49** |  | 1.03 |  | 1.03 | 1.03 | 1.03 | 1.03 | 1.03 | 1.03 | 1.03 | 1.02 | 1.02 | 1.02 |
| **0.57** | **0.5** |  | 1.02 |  | 1.02 | 1.02 | 1.02 | 1.02 | 1.01 | 1.01 | 1.01 | 1.01 | 1.01 | 1.01 |
| **0.59** | **0.51** |  | 1.01 |  | 1.01 | 1.01 | 1.00 | 1.00 | 1.00 | 1.00 | 1.00 | 1.00 | 1.00 | 1.00 |
| **0.61** | **0.52** |  | 1.00 |  | 1.00 | 0.99 | 0.99 | 0.99 | 0.99 | 0.99 | 0.99 | 0.99 | 0.99 | 0.98 |
| **0.63** | **0.53** |  | 0.99 |  | 0.99 | 0.98 | 0.98 | 0.98 | 0.98 | 0.98 | 0.98 | 0.97 | 0.97 | 0.97 |
| **0.65** | **0.54** |  | 0.98 |  | 0.97 | 0.97 | 0.97 | 0.97 | 0.97 | 0.97 | 0.96 | 0.96 | 0.96 | 0.96 |
|  |  |  |  |  |  |  |  |  |  |  |  |  |  |  |

* Prevalence of smoking at Camp Lejeune

# Prevalence of smoking at Camp Pendleton

RR for smoking and lung cancer was assumed to range between 7.00 and 12.00 [4].

The reduction in the HR for lung cancer by adjusting for a 4% difference in smoking prevalence between Camp Lejeune and Camp Pendleton would be from a HR of 1.13 to HRs between 1.05 and 1.06 (**bolded**).

CL – Camp Lejeune

CP – Camp Pendleton

HR – hazard ratio

RR – risk ratio

**Table S16**. Cancer of the larynx (as an underlying cause) hazard ratio = 1.19. Adjusted for a 4% smoking prevalence (p) difference between the Camp Lejeune and Camp Pendleton civilian employees.

| **Multidimensional RR CL-larynx Relationship Adjusted for smoking** | | | | | | | | | | | | | | |
| --- | --- | --- | --- | --- | --- | --- | --- | --- | --- | --- | --- | --- | --- | --- |
|  |  |  |  |  |  |  |  |  |  |  |  |  |  |  |
| **p(smoking+\|CL+)*** | **p(smoking+\|CL-)^#^** | **RR(smoking-larynx)** | **7** |  | **7.5** | **8** | **8.5** | **9** | **9.5** | **10** | **10.5** | **11** | **11.5** | **12** |
| **0.45** | **0.44** |  | 1.17 |  | 1.17 | 1.17 | 1.17 | 1.17 | 1.17 | 1.17 | 1.17 | 1.17 | 1.17 | 1.17 |
| **0.47** | **0.45** |  | 1.15 |  | 1.15 | 1.15 | 1.15 | 1.15 | 1.15 | 1.15 | 1.15 | 1.15 | 1.15 | 1.15 |
| **0.49** | **0.46** |  | 1.13 |  | 1.13 | 1.13 | 1.13 | 1.13 | 1.13 | 1.13 | 1.13 | 1.13 | 1.13 | 1.13 |
| **0.51** | **0.47** |  | **1.12** |  | **1.12** | **1.11** | **1.11** | **1.11** | **1.11** | **1.11** | **1.11** | **1.11** | **1.11** | **1.11** |
| **0.53** | **0.48** |  | 1.10 |  | 1.10 | 1.10 | 1.10 | 1.10 | 1.10 | 1.10 | 1.09 | 1.09 | 1.09 | 1.09 |
| **0.55** | **0.49** |  | 1.09 |  | 1.09 | 1.08 | 1.08 | 1.08 | 1.08 | 1.08 | 1.08 | 1.08 | 1.08 | 1.08 |
| **0.57** | **0.5** |  | 1.07 |  | 1.07 | 1.07 | 1.07 | 1.07 | 1.07 | 1.07 | 1.06 | 1.06 | 1.06 | 1.06 |
| **0.59** | **0.51** |  | 1.06 |  | 1.06 | 1.06 | 1.06 | 1.05 | 1.05 | 1.05 | 1.05 | 1.05 | 1.05 | 1.05 |
| **0.61** | **0.52** |  | 1.05 |  | 1.05 | 1.05 | 1.04 | 1.04 | 1.04 | 1.04 | 1.04 | 1.04 | 1.04 | 1.04 |
| **0.63** | **0.53** |  | 1.04 |  | 1.04 | 1.03 | 1.03 | 1.03 | 1.03 | 1.03 | 1.03 | 1.03 | 1.02 | 1.02 |
| **0.65** | **0.54** |  | 1.03 |  | 1.03 | 1.02 | 1.02 | 1.02 | 1.02 | 1.02 | 1.01 | 1.01 | 1.01 | 1.01 |

* Prevalence of smoking at Camp Lejeune

# Prevalence of smoking at Camp Pendleton

In one study, RRs for smoking and laryngeal cancer ranged from 4.65 (ex-smoker) to 6.98 (current smoker) [4]. In another study, the RR for current smokers among men was 13.9 [9].

The reduction in the HR for laryngeal cancer by adjusting for a 4% difference in smoking prevalence between Camp Lejeune and Camp Pendleton would be from a HR of 1.19 to HRs between 1.11 and 1.12 (**bolded**).

CL – Camp Lejeune

CP – Camp Pendleton

HR – hazard ratio

RR – risk ratio

**Table S17**. Cancer of the larynx (as a contributing cause) hazard ratio = 1.69. Adjusted for a 4% smoking prevalence (p) difference between Camp Lejeune and Camp Pendleton civilian employees.

| **Multidimensional RR CL-larynx Relationship Adjusted for smoking** | | | | | | | | | | | | | | |
| --- | --- | --- | --- | --- | --- | --- | --- | --- | --- | --- | --- | --- | --- | --- |
|  |  |  |  |  |  |  |  |  |  |  |  |  |  |  |
| **p(smoking+\|CL+)*** | **p(smoking+\|CL-)^#^** | **RR(smoking-larynx)** | **7** |  | **7.5** | **8** | **8.5** | **9** | **9.5** | **10** | **10.5** | **11** | **11.5** | **12** |
| **0.45** | **0.44** |  | 1.66 |  | 1.66 | 1.66 | 1.66 | 1.66 | 1.66 | 1.65 | 1.65 | 1.65 | 1.65 | 1.65 |
| **0.47** | **0.45** |  | 1.63 |  | 1.63 | 1.63 | 1.63 | 1.63 | 1.63 | 1.63 | 1.63 | 1.63 | 1.63 | 1.62 |
| **0.49** | **0.46** |  | 1.61 |  | 1.61 | 1.61 | 1.60 | 1.60 | 1.60 | 1.60 | 1.60 | 1.60 | 1.60 | 1.60 |
| **0.51** | **0.47** |  | **1.59** |  | **1.58** | **1.58** | **1.58** | **1.58** | **1.58** | **1.58** | **1.58** | **1.57** | **1.57** | **1.57** |
| **0.53** | **0.48** |  | 1.56 |  | 1.56 | 1.56 | 1.56 | 1.56 | 1.55 | 1.55 | 1.55 | 1.55 | 1.55 | 1.55 |
| **0.55** | **0.49** |  | 1.54 |  | 1.54 | 1.54 | 1.54 | 1.54 | 1.53 | 1.53 | 1.53 | 1.53 | 1.53 | 1.53 |
| **0.57** | **0.5** |  | 1.52 |  | 1.52 | 1.52 | 1.52 | 1.52 | 1.51 | 1.51 | 1.51 | 1.51 | 1.51 | 1.51 |
| **0.59** | **0.51** |  | 1.51 |  | 1.50 | 1.50 | 1.50 | 1.50 | 1.49 | 1.49 | 1.49 | 1.49 | 1.49 | 1.49 |
| **0.61** | **0.52** |  | 1.49 |  | 1.49 | 1.48 | 1.48 | 1.48 | 1.48 | 1.47 | 1.47 | 1.47 | 1.47 | 1.47 |
| **0.63** | **0.53** |  | 1.47 |  | 1.47 | 1.47 | 1.46 | 1.46 | 1.46 | 1.46 | 1.46 | 1.45 | 1.45 | 1.45 |
| **0.65** | **0.54** |  | 1.46 |  | 1.45 | 1.45 | 1.45 | 1.45 | 1.44 | 1.44 | 1.44 | 1.44 | 1.44 | 1.43 |

* Prevalence of smoking at Camp Lejeune

# Prevalence of smoking at Camp Pendleton

In one study, RRs for smoking and laryngeal cancer ranged from 4.65 (ex-smoker) to 6.98 (current smoker) [4]. In another study, the RR for current smokers among men was 13.9 [9].

The reduction in the HR for laryngeal cancer by adjusting for a 4% difference in smoking prevalence between Camp Lejeune and Camp Pendleton would be from a HR of 1.69 to HRs between 1.57 and 1.59 (**bolded**).

CL – Camp Lejeune

CP – Camp Pendleton

HR – hazard ratio

RR – risk ratio

**Table S18**. Pharyngeal cancer hazard ratio = 2.21. Adjusted for a 4% smoking prevalence (p) difference between the Camp Lejeune and Camp Pendleton civilian **employees.**

| **Multidimensional RR CL-pharyngeal cancer Relationship Adjusted for smoking** | | | | | | | | | | | | | | |
| --- | --- | --- | --- | --- | --- | --- | --- | --- | --- | --- | --- | --- | --- | --- |
|  |  |  |  |  |  |  |  |  |  |  |  |  |  |  |
| **p(smoking+\|CL+)*** | **p(smoking+\|CL-)^#^** | **RR(smoking-pharyngeal cancer)** | **5** |  | **5.25** | **5.5** | **5.75** | **6** | **6.25** | **6.5** | **6.75** | **7** | **7.25** | **7.5** |
| **0.45** | **0.44** |  | 2.17 |  | 2.17 | 2.17 | 2.17 | 2.17 | 2.17 | 2.17 | 2.17 | 2.17 | 2.17 | 2.17 |
| **0.47** | **0.45** |  | 2.14 |  | 2.14 | 2.14 | 2.14 | 2.14 | 2.14 | 2.14 | 2.13 | 2.13 | 2.13 | 2.13 |
| **0.49** | **0.46** |  | 2.11 |  | 2.11 | 2.11 | 2.11 | 2.11 | 2.11 | 2.10 | 2.10 | 2.10 | 2.10 | 2.10 |
| **0.51** | **0.47** |  | **2.09** |  | **2.08** | **2.08** | **2.08** | **2.08** | **2.08** | **2.08** | **2.07** | **2.07** | **2.07** | **2.07** |
| **0.53** | **0.48** |  | 2.06 |  | 2.06 | 2.06 | 2.05 | 2.05 | 2.05 | 2.05 | 2.05 | 2.05 | 2.04 | 2.04 |
| **0.55** | **0.49** |  | 2.04 |  | 2.03 | 2.03 | 2.03 | 2.03 | 2.02 | 2.02 | 2.02 | 2.02 | 2.02 | 2.02 |
| **0.57** | **0.5** |  | 2.02 |  | 2.01 | 2.01 | 2.01 | 2.00 | 2.00 | 2.00 | 2.00 | 1.99 | 1.99 | 1.99 |
| **0.59** | **0.51** |  | 1.99 |  | 1.99 | 1.99 | 1.98 | 1.98 | 1.98 | 1.97 | 1.97 | 1.97 | 1.97 | 1.97 |
| **0.61** | **0.52** |  | 1.97 |  | 1.97 | 1.96 | 1.96 | 1.96 | 1.96 | 1.95 | 1.95 | 1.95 | 1.95 | 1.94 |
| **0.63** | **0.53** |  | 1.95 |  | 1.95 | 1.94 | 1.94 | 1.94 | 1.93 | 1.93 | 1.93 | 1.93 | 1.92 | 1.92 |
| **0.65** | **0.54** |  | 1.93 |  | 1.93 | 1.93 | 1.92 | 1.92 | 1.91 | 1.91 | 1.91 | 1.91 | 1.90 | 1.90 |

* Prevalence of smoking at Camp Lejeune

# Prevalence of smoking at Camp Pendleton

RR for current smoking and cancer of the pharynx was 6.76 in a pooled analysis [4].

The reduction in the HR for pharyngeal cancer by adjusting for a 4% difference in smoking prevalence between Camp Lejeune and Camp Pendleton would be from a HR of 2.21 to HRs between 2.07 and 2.09 (**bolded**).

CL – Camp Lejeune

CP – Camp Pendleton

HR – hazard ratio

RR – risk ratio

**Table S19**. Kidney cancer hazard ratio = 1.44. Adjusted for a 4% smoking prevalence (p) difference between the Camp Lejeune and Camp Pendleton civilian employees.

| **Multidimensional RR CL-kidney cancer Relationship Adjusted for smoking** | | | | | | | | | | | | | | |
| --- | --- | --- | --- | --- | --- | --- | --- | --- | --- | --- | --- | --- | --- | --- |
|  |  |  |  |  |  |  |  |  |  |  |  |  |  |  |
| **p(smoking+\|CL+)*** | **p(smoking+\|CL-)^#^** | **RR(smoking- kidney cancer)** | **1.3** |  | **1.35** | **1.4** | **1.45** | **1.5** | **1.55** | **1.6** | **1.65** | **1.7** | **1.75** | **1.8** |
| **0.45** | **0.44** |  | 1.43 |  | 1.43 | 1.43 | 1.43 | 1.43 | 1.43 | 1.43 | 1.43 | 1.43 | 1.43 | 1.43 |
| **0.47** | **0.45** |  | 1.43 |  | 1.43 | 1.43 | 1.42 | 1.42 | 1.42 | 1.42 | 1.42 | 1.42 | 1.42 | 1.42 |
| **0.49** | **0.46** |  | 1.42 |  | 1.42 | 1.42 | 1.42 | 1.42 | 1.42 | 1.42 | 1.41 | 1.41 | 1.41 | 1.41 |
| **0.51** | **0.47** |  | **1.42** |  | **1.42** | **1.42** | **1.41** | **1.41** | **1.41** | **1.41** | **1.41** | **1.41** | **1.40** | **1.40** |
| **0.53** | **0.48** |  | 1.42 |  | 1.41 | 1.41 | 1.41 | 1.41 | 1.40 | 1.40 | 1.40 | 1.40 | 1.40 | 1.39 |
| **0.55** | **0.49** |  | 1.41 |  | 1.41 | 1.41 | 1.40 | 1.40 | 1.40 | 1.40 | 1.39 | 1.39 | 1.39 | 1.39 |
| **0.57** | **0.5** |  | 1.41 |  | 1.41 | 1.40 | 1.40 | 1.40 | 1.39 | 1.39 | 1.39 | 1.38 | 1.38 | 1.38 |
| **0.59** | **0.51** |  | 1.41 |  | 1.40 | 1.40 | 1.39 | 1.39 | 1.39 | 1.38 | 1.38 | 1.38 | 1.38 | 1.37 |
| **0.61** | **0.52** |  | 1.40 |  | 1.40 | 1.39 | 1.39 | 1.39 | 1.38 | 1.38 | 1.37 | 1.37 | 1.37 | 1.37 |
| **0.63** | **0.53** |  | 1.40 |  | 1.39 | 1.39 | 1.38 | 1.38 | 1.38 | 1.37 | 1.37 | 1.37 | 1.36 | 1.36 |
| **0.65** | **0.54** |  | 1.40 |  | 1.39 | 1.38 | 1.38 | 1.38 | 1.37 | 1.37 | 1.36 | 1.36 | 1.36 | 1.35 |

* Prevalence of smoking at Camp Lejeune

# Prevalence of smoking at Camp Pendleton

RRs for smoking and kidney cancer were assumed to be between 1.3 and 1.8 [4,9].

The reduction in the HR for kidney cancer by adjusting for a 4% difference in smoking prevalence between Camp Lejeune and Camp Pendleton would be from a HR of 1.44 to HRs between 1.40 and 1.42 (**bolded**).

CL – Camp Lejeune

CP – Camp Pendleton

HR – hazard ratio

RR – risk ratio

**Table S20**. Chronic kidney disease hazard ratio = 1.88. Adjusted for a 4% smoking prevalence (p) difference between the Camp Lejeune and Camp Pendleton civilian employees.

| **Multidimensional RR CL-chronic kidney disease Relationship Adjusted for smoking** | | | | | | | | | | | | | | |
| --- | --- | --- | --- | --- | --- | --- | --- | --- | --- | --- | --- | --- | --- | --- |
|  |  |  |  |  |  |  |  |  |  |  |  |  |  |  |
| **p(smoking+\|CL+)*** | **p(smoking+\|CL-)^#^** | **RR(smoking-chronic kidney disease)** | **1.3** |  | **1.35** | **1.4** | **1.45** | **1.5** | **1.55** | **1.6** | **1.65** | **1.7** | **1.75** | **1.8** |
| **0.45** | **0.44** |  | 1.87 |  | 1.87 | 1.87 | 1.87 | 1.87 | 1.87 | 1.87 | 1.87 | 1.87 | 1.86 | 1.86 |
| **0.47** | **0.45** |  | 1.87 |  | 1.86 | 1.86 | 1.86 | 1.86 | 1.86 | 1.86 | 1.86 | 1.86 | 1.85 | 1.85 |
| **0.49** | **0.46** |  | 1.86 |  | 1.86 | 1.86 | 1.85 | 1.85 | 1.85 | 1.85 | 1.85 | 1.85 | 1.84 | 1.84 |
| **0.51** | **0.47** |  | **1.86** |  | **1.85** | **1.85** | **1.85** | **1.85** | **1.84** | **1.84** | **1.84** | **1.84** | **1.83** | **1.83** |
| **0.53** | **0.48** |  | 1.85 |  | 1.85 | 1.84 | 1.84 | 1.84 | 1.84 | 1.83 | 1.83 | 1.83 | 1.83 | 1.82 |
| **0.55** | **0.49** |  | 1.85 |  | 1.84 | 1.84 | 1.83 | 1.83 | 1.83 | 1.82 | 1.82 | 1.82 | 1.82 | 1.81 |
| **0.57** | **0.5** |  | 1.84 |  | 1.84 | 1.83 | 1.83 | 1.82 | 1.82 | 1.82 | 1.81 | 1.81 | 1.81 | 1.80 |
| **0.59** | **0.51** |  | 1.84 |  | 1.83 | 1.83 | 1.82 | 1.82 | 1.81 | 1.81 | 1.80 | 1.80 | 1.80 | 1.79 |
| **0.61** | **0.52** |  | 1.83 |  | 1.83 | 1.82 | 1.82 | 1.81 | 1.81 | 1.80 | 1.80 | 1.79 | 1.79 | 1.78 |
| **0.63** | **0.53** |  | 1.83 |  | 1.82 | 1.82 | 1.81 | 1.80 | 1.80 | 1.79 | 1.79 | 1.78 | 1.78 | 1.78 |
| **0.65** | **0.54** |  | 1.82 |  | 1.82 | 1.81 | 1.80 | 1.80 | 1.79 | 1.79 | 1.78 | 1.78 | 1.77 | 1.77 |

* Prevalence of smoking at Camp Lejeune

# Prevalence of smoking at Camp Pendleton

The RRs for smoking and renal failure among men ranged from 1.5 (ex-smoker) to 2.1 (current smoker), and among women, the RRs ranged from 1.3 (ex-smoker) to 1.9 (current smoker) [9].

The reduction in the HR for chronic kidney disease by adjusting for a 4% difference in smoking prevalence between Camp Lejeune and Camp Pendleton would be from a HR of 1.88 to HRs between 1.83 and 1.86 (**bolded**).

CL – Camp Lejeune

CP – Camp Pendleton

HR – hazard ratio

RR – risk ratio

**Table S21**. Parkinson disease hazard ratio = 1.21. Adjusted for a 4% smoking prevalence (p) difference between the Camp Lejeune and Camp Pendleton civilian employees.

| **Multidimensional RR CL-Parkinson Relationship Adjusted for smoking** | | | | | | | | | | | | | | |
| --- | --- | --- | --- | --- | --- | --- | --- | --- | --- | --- | --- | --- | --- | --- |
|  |  |  |  |  |  |  |  |  |  |  |  |  |  |  |
| **p(smoking+\|CL+)*** | **p(smoking+\|CL-)^#^** | **RR(smoking-Parkinson)** | **0.3** |  | **0.35** | **0.4** | **0.45** | **0.5** | **0.55** | **0.6** | **0.65** | **0.7** | **0.8** | **0.85** |
| **0.45** | **0.44** |  | 1.22 |  | 1.22 | 1.22 | 1.22 | 1.22 | 1.22 | 1.22 | 1.22 | 1.21 | 1.21 | 1.21 |
| **0.47** | **0.45** |  | 1.24 |  | 1.23 | 1.23 | 1.23 | 1.23 | 1.22 | 1.22 | 1.22 | 1.22 | 1.22 | 1.21 |
| **0.49** | **0.46** |  | 1.25 |  | 1.25 | 1.24 | 1.24 | 1.23 | 1.23 | 1.23 | 1.23 | 1.22 | 1.22 | 1.22 |
| **0.51** | **0.47** |  | **1.26** |  | **1.26** | **1.25** | **1.25** | **1.24** | **1.24** | **1.24** | **1.23** | **1.23** | **1.22** | **1.22** |
| **0.53** | **0.48** |  | 1.28 |  | 1.27 | 1.26 | 1.26 | 1.25 | 1.25 | 1.24 | 1.24 | 1.23 | 1.22 | 1.22 |
| **0.55** | **0.49** |  | 1.29 |  | 1.28 | 1.28 | 1.27 | 1.26 | 1.25 | 1.25 | 1.24 | 1.24 | 1.23 | 1.22 |
| **0.57** | **0.5** |  | 1.31 |  | 1.30 | 1.29 | 1.28 | 1.27 | 1.26 | 1.25 | 1.25 | 1.24 | 1.23 | 1.22 |
| **0.59** | **0.51** |  | 1.33 |  | 1.31 | 1.30 | 1.29 | 1.28 | 1.27 | 1.26 | 1.25 | 1.25 | 1.23 | 1.23 |
| **0.61** | **0.52** |  | 1.34 |  | 1.33 | 1.31 | 1.30 | 1.29 | 1.28 | 1.27 | 1.26 | 1.25 | 1.24 | 1.23 |
| **0.63** | **0.53** |  | 1.36 |  | 1.34 | 1.33 | 1.31 | 1.30 | 1.29 | 1.28 | 1.27 | 1.26 | 1.24 | 1.23 |
| **0.65** | **0.54** |  | 1.38 |  | 1.36 | 1.34 | 1.32 | 1.31 | 1.30 | 1.28 | 1.27 | 1.26 | 1.24 | 1.23 |

* Prevalence of smoking at Camp Lejeune

# Prevalence of smoking at Camp Pendleton

RRs for smoking and Parkinson disease range from 0.23 to 0.53 for current smokers to 0.80 (ex-smoker) [7-8].

The increase in the HR for Parkinson disease by adjusting for a 4% difference in smoking prevalence between Camp Lejeune and Camp Pendleton would be from a HR of 1.21 to HRs between 1.22 and 1.26 (**bolded**).

CL – Camp Lejeune

CP – Camp Pendleton

HR – hazard ratio

RR – risk ratio

**Table S22**. Chronic liver disease mortality (as an underlying cause) hazard ratio = 0.93. Adjusted for alcohol consumption prevalence (p) differences between the Camp Lejeune and Camp Pendleton Marines/Navy personnel subgroup.

| **Multidimensional RR CL-chronic liver disease mortality Relationship Adjusted for alcohol use** | | | | | | | | | | | | | | |
| --- | --- | --- | --- | --- | --- | --- | --- | --- | --- | --- | --- | --- | --- | --- |
|  |  |  |  |  |  |  |  |  |  |  |  |  |  |  |
| **p(alcohol use+\|CL+)*** | **p(alcohol use+\|CL-)^#^** | **RR(alcohol use-chronic liver disease mortality)** | **2.5** |  | **3** | **3.5** | **4** | **5** | **6** | **6.5** | **7** | **8** | **9** | **10** |
| **0.67** | **0.69** |  | 0.94 |  | 0.94 | 0.95 | 0.95 | 0.95 | 0.95 | 0.95 | 0.95 | 0.95 | 0.95 | 0.95 |
| **0.67** | **0.7** |  | 0.95 |  | 0.95 | 0.95 | 0.96 | 0.96 | 0.96 | 0.96 | 0.96 | 0.96 | 0.96 | 0.96 |
| **0.67** | **0.71** |  | 0.96 |  | 0.96 | 0.96 | 0.97 | 0.97 | 0.97 | 0.97 | 0.97 | 0.97 | 0.97 | 0.98 |
| **0.67** | **0.72** |  | 0.96 |  | 0.97 | 0.97 | 0.97 | 0.98 | 0.98 | 0.98 | 0.98 | 0.99 | 0.99 | 0.99 |
| **0.67** | **0.73** |  | 0.97 |  | 0.98 | 0.98 | 0.98 | 0.99 | 0.99 | 0.99 | 0.99 | **1.00** | **1.00** | **1.00** |
| **0.67** | **0.74** |  | 0.98 |  | 0.98 | 0.99 | 0.99 | **1.00** | **1.00** | **1.00** | 1.01 | 1.01 | 1.01 | 1.01 |
| **0.67** | **0.75** |  | 0.98 |  | 0.99 | **1.00** | **1.00** | 1.01 | 1.01 | 1.02 | 1.02 | 1.02 | 1.02 | 1.02 |
| **0.67** | **0.76** |  | 0.99 |  | **1.00** | 1.01 | 1.01 | 1.02 | 1.02 | 1.03 | 1.03 | 1.03 | 1.03 | 1.04 |
| **0.67** | **0.77** |  | **1.00** |  | 1.01 | 1.02 | 1.02 | 1.03 | 1.03 | 1.04 | 1.04 | 1.04 | 1.05 | 1.05 |
| **0.67** | **0.78** |  | 1.00 |  | 1.02 | 1.02 | 1.03 | 1.04 | 1.05 | 1.05 | 1.05 | 1.05 | 1.06 | 1.06 |
| **0.67** | **0.79** |  | 1.01 |  | 1.02 | 1.03 | 1.04 | 1.05 | 1.06 | 1.06 | 1.06 | 1.07 | 1.07 | 1.07 |

* Prevalence of alcohol consumption at Camp Lejeune

# Prevalence of alcohol consumption at Camp Pendleton

A systematic review of alcohol consumption and mortality due to liver cirrhosis found RRs of 2.65, 6.83 and 16.38 for drinking 25g/day (2 drinks/day), 50g/day (4 drinks/day) and 100g/day (8 drinks/day) compared to those who never drank alcoholic beverages [6]. The bias analysis used RRs ranging between 2.5 and 10 for alcohol consumption and chronic liver disease. A military survey conducted in 1980 found that about 30% of Marines were heavy drinkers [2]. The bias analysis assumed that at least 2/3 of Marines/Navy personnel at Camp Lejeune consumed ≥1 drink/day.

To fully explain the HR of 0.93 for chronic liver disease mortality and base location, the prevalence difference between Camp Lejeune and Camp Pendleton would range between 6% and 10% (**bolded**). (If only half the Marines/Navy personnel at Camp Lejeune were drinkers, then the percentage difference ranges would be 5% - 9%)

CL – Camp Lejeune CP – Camp Pendleton HR – hazard ratio RR – risk ratio

**Table S23**. Esophageal cancer mortality hazard ratio = 1.24. Adjusted for a 8% alcohol consumption prevalence (p) difference between the Camp Lejeune and Camp Pendleton Marines/Navy personnel subgroup.

| **Multidimensional RR CL-esophageal cancer deaths Relationship Adjusted for alcohol use** | | | | | | | | | | | | | | |
| --- | --- | --- | --- | --- | --- | --- | --- | --- | --- | --- | --- | --- | --- | --- |
|  |  |  |  |  |  |  |  |  |  |  |  |  |  |  |
| **p(alcohol use+\|CL+)*** | **p(alcohol use+\|CL-)^#^** | **RR(alcohol use-esophageal cancer deaths)** | **1.25** |  | **1.5** | **1.75** | **2** | **2.5** | **3** | **3.5** | **4** | **4.5** | **5** | **5.25** |
| **0.67** | **0.68** |  | 1.24 |  | 1.24 | 1.25 | 1.25 | 1.25 | 1.25 | 1.25 | 1.25 | 1.25 | 1.25 | 1.25 |
| **0.67** | **0.69** |  | 1.24 |  | 1.25 | 1.25 | 1.25 | 1.26 | 1.26 | 1.26 | 1.26 | 1.27 | 1.27 | 1.27 |
| **0.67** | **0.7** |  | 1.25 |  | 1.25 | 1.26 | 1.26 | 1.27 | 1.27 | 1.27 | 1.28 | 1.28 | 1.28 | 1.28 |
| **0.67** | **0.71** |  | 1.25 |  | 1.26 | 1.26 | 1.27 | 1.28 | 1.28 | 1.29 | 1.29 | 1.29 | 1.29 | 1.29 |
| **0.67** | **0.72** |  | 1.25 |  | 1.26 | 1.27 | 1.28 | 1.29 | 1.29 | 1.30 | 1.30 | 1.30 | 1.31 | 1.31 |
| **0.67** | **0.73** |  | 1.26 |  | 1.27 | 1.28 | 1.28 | 1.30 | 1.30 | 1.31 | 1.31 | 1.32 | 1.32 | 1.32 |
| **0.67** | **0.74** |  | 1.26 |  | 1.27 | 1.28 | 1.29 | 1.30 | 1.31 | 1.32 | 1.33 | 1.33 | 1.33 | 1.34 |
| **0.67** | **0.75** |  | **1.26** |  | **1.28** | **1.29** | **1.30** | **1.31** | **1.32** | **1.33** | **1.34** | **1.34** | **1.35** | **1.35** |
| **0.67** | **0.76** |  | 1.26 |  | 1.28 | 1.30 | 1.31 | 1.32 | 1.33 | 1.34 | 1.35 | 1.36 | 1.36 | 1.36 |
| **0.67** | **0.77** |  | 1.27 |  | 1.29 | 1.30 | 1.31 | 1.33 | 1.35 | 1.36 | 1.36 | 1.37 | 1.37 | 1.38 |
| **0.67** | **0.78** |  | 1.27 |  | 1.29 | 1.31 | 1.32 | 1.34 | 1.36 | 1.37 | 1.38 | 1.38 | 1.39 | 1.39 |

* Prevalence of alcohol consumption at Camp Lejeune

# Prevalence of alcohol consumption at Camp Pendleton

RRs for alcohol consumption and esophageal cancer were assumed to be similar to the RRs for squamous cell esophageal cancer:

RR for squamous cell esophageal cancer and moderate alcohol use (12.5g/day – 50g/day) = 2.23; and RR for squamous cell esophageal cancer and heavy alcohol use (>50g/day) = 4.95.^12^ RRs for esophageal cancer range from 1.24 (<1 drink per week) to 3.99 (≥3 drinks per day) [11].

The increase in the HR for esophageal cancer by adjusting for a 8% difference in alcohol use prevalence between Camp Lejeune and Camp Pendleton would be from a HR of 1.24 to HRs between 1.26 and 1.35 (**bolded**).

CL – Camp Lejeune CP – Camp Pendleton HR – hazard ratio RR – risk ratio

**Table S24**. Cancer of the larynx mortality (as a contributing cause) hazard ratio = 1.14. Adjusted for a 8% alcohol consumption prevalence (p) difference between the Camp Lejeune and Camp Pendleton Marines/Navy personnel subgroup.

| **Multidimensional RR CL-laryngeal cancer Relationship Adjusted for smoking** | | | | | | | | | | | | | | |
| --- | --- | --- | --- | --- | --- | --- | --- | --- | --- | --- | --- | --- | --- | --- |
|  |  |  |  |  |  |  |  |  |  |  |  |  |  |  |
| **p(smoking+\|CL+)** | **p(smoking+\|CL-)** | **RR(smoking-laryngeal cancer)** | **1.2** |  | **1.3** | **1.5** | **1.7** | **1.9** | **2.1** | **2.3** | **2.5** | **2.7** | **2.9** | **3** |
| **0.67** | **0.68** |  | 1.14 |  | 1.14 | 1.14 | 1.14 | 1.14 | 1.15 | 1.15 | 1.15 | 1.15 | 1.15 | 1.15 |
| **0.67** | **0.69** |  | 1.14 |  | 1.14 | 1.15 | 1.15 | 1.15 | 1.15 | 1.15 | 1.16 | 1.16 | 1.16 | 1.16 |
| **0.67** | **0.7** |  | 1.14 |  | 1.15 | 1.15 | 1.15 | 1.16 | 1.16 | 1.16 | 1.16 | 1.17 | 1.17 | 1.17 |
| **0.67** | **0.71** |  | 1.15 |  | 1.15 | 1.16 | 1.16 | 1.16 | 1.17 | 1.17 | 1.17 | 1.17 | 1.18 | 1.18 |
| **0.67** | **0.72** |  | 1.15 |  | 1.15 | 1.16 | 1.17 | 1.17 | 1.17 | 1.18 | 1.18 | 1.18 | 1.19 | 1.19 |
| **0.67** | **0.73** |  | 1.15 |  | 1.16 | 1.16 | 1.17 | 1.18 | 1.18 | 1.19 | 1.19 | 1.19 | 1.20 | 1.20 |
| **0.67** | **0.74** |  | 1.15 |  | 1.16 | 1.17 | 1.18 | 1.18 | 1.19 | 1.19 | 1.20 | 1.20 | 1.20 | 1.21 |
| **0.67** | **0.75** |  | **1.15** |  | **1.16** | **1.17** | **1.18** | **1.19** | **1.20** | **1.20** | **1.21** | **1.21** | **1.21** | **1.22** |
| **0.67** | **0.76** |  | 1.16 |  | 1.16 | 1.18 | 1.19 | 1.20 | 1.20 | 1.21 | 1.21 | 1.22 | 1.22 | 1.23 |
| **0.67** | **0.77** |  | 1.16 |  | 1.17 | 1.18 | 1.19 | 1.20 | 1.21 | 1.22 | 1.22 | 1.23 | 1.23 | 1.24 |
| **0.67** | **0.78** |  | 1.16 |  | 1.17 | 1.19 | 1.20 | 1.21 | 1.22 | 1.23 | 1.23 | 1.24 | 1.24 | 1.25 |

* Prevalence of alcohol consumption at Camp Lejeune

# Prevalence of alcohol consumption at Camp Pendleton

RR for laryngeal cancer and moderate alcohol use (12.5g/day – 50g/day) = 1.44; RR for laryngeal cancer and heavy alcohol use (>50g/day) = 2.65 12].

The increase in the HR for laryngeal cancer by adjusting for a 8% difference in alcohol use prevalence between Camp Lejeune and Camp Pendleton would be from a HR of 1.14 to HRs between 1.15 and 1.22 (**bolded**).

CL – Camp Lejeune CP – Camp Pendleton

HR – hazard ratio RR – risk ratio

**Table S25**. Female breast cancer mortality hazard ratio = 1.20. Adjusted for a 8% alcohol consumption prevalence (p) difference between the Camp Lejeune and Camp Pendleton Marines/Navy personnel subgroup.

| **Multidimensional RR CL-female breast cancer Relationship Adjusted for alcohol use** | | | | | | | | | | | | | | |
| --- | --- | --- | --- | --- | --- | --- | --- | --- | --- | --- | --- | --- | --- | --- |
|  |  |  |  |  |  |  |  |  |  |  |  |  |  |  |
| **p(alcohol use+\|CL+)*** | **p(alcohol use+\|CL-)^#^** | **RR(alcohol use-female breast cancer)** | **1.1** |  | **1.15** | **1.2** | **1.25** | **1.3** | **1.35** | **1.4** | **1.45** | **1.5** | **1.55** | **1.6** |
| **0.67** | **0.68** |  | 1.20 |  | 1.20 | 1.20 | 1.20 | 1.20 | 1.20 | 1.20 | 1.21 | 1.21 | 1.21 | 1.21 |
| **0.67** | **0.69** |  | 1.20 |  | 1.20 | 1.21 | 1.21 | 1.21 | 1.21 | 1.21 | 1.21 | 1.21 | 1.21 | 1.21 |
| **0.67** | **0.7** |  | 1.20 |  | 1.21 | 1.21 | 1.21 | 1.21 | 1.21 | 1.21 | 1.21 | 1.21 | 1.22 | 1.22 |
| **0.67** | **0.71** |  | 1.21 |  | 1.21 | 1.21 | 1.21 | 1.21 | 1.21 | 1.22 | 1.22 | 1.22 | 1.22 | 1.22 |
| **0.67** | **0.72** |  | 1.21 |  | 1.21 | 1.21 | 1.21 | 1.22 | 1.22 | 1.22 | 1.22 | 1.22 | 1.23 | 1.23 |
| **0.67** | **0.73** |  | 1.21 |  | 1.21 | 1.21 | 1.22 | 1.22 | 1.22 | 1.22 | 1.23 | 1.23 | 1.23 | 1.23 |
| **0.67** | **0.74** |  | 1.21 |  | 1.21 | 1.22 | 1.22 | 1.22 | 1.22 | 1.23 | 1.23 | 1.23 | 1.23 | 1.24 |
| **0.67** | **0.75** |  | **1.21** |  | **1.21** | **1.22** | **1.22** | **1.22** | **1.23** | **1.23** | **1.23** | **1.24** | **1.24** | **1.24** |
| **0.67** | **0.76** |  | 1.21 |  | 1.22 | 1.22 | 1.22 | 1.23 | 1.23 | 1.24 | 1.24 | 1.24 | 1.24 | 1.25 |
| **0.67** | **0.77** |  | 1.21 |  | 1.22 | 1.22 | 1.23 | 1.23 | 1.24 | 1.24 | 1.24 | 1.25 | 1.25 | 1.25 |
| **0.67** | **0.78** |  | 1.21 |  | 1.22 | 1.22 | 1.23 | 1.23 | 1.24 | 1.24 | 1.25 | 1.25 | 1.25 | 1.26 |

* Prevalence of alcohol consumption at Camp Lejeune

# Prevalence of alcohol consumption at Camp Pendleton

RR for female breast cancer and moderate alcohol use (12.5g/day – 50g/day) = 1.23 [12].

RR for female breast cancer and heavy alcohol use (>50g/day) = 1.61 [12].

The increase in the HR for female breast cancer by adjusting for a 8% difference in alcohol use prevalence between Camp Lejeune and Camp Pendleton would be from a HR of 1.20 to HRs between 1.21 and 1.24 (**bolded**).

CL – Camp Lejeune

CP – Camp Pendleton

HR – hazard ratio

RR – risk ratio

**Table S26**. Chronic liver disease mortality hazard ratio = 0.74. Adjusted for alcohol consumption prevalence (p) differences between the Camp Lejeune and Camp Pendleton civilian employees.

| **Multidimensional RR CL-chronic liver disease mortality Relationship Adjusted for alcohol use** | | | | | | | | | | | | | | |
| --- | --- | --- | --- | --- | --- | --- | --- | --- | --- | --- | --- | --- | --- | --- |
|  |  |  |  |  |  |  |  |  |  |  |  |  |  |  |
| **p(alcohol use+\|CL+)*** | **p(alcohol use+\|CL-)^#^** | **RR(alcohol use-chronic liver disease mortality)** | **2.5** |  | **3** | **3.5** | **4** | **5** | **6** | **6.5** | **7** | **8** | **9** | **10** |
| **0.33** | **0.48** |  | 0.86 |  | 0.88 | 0.90 | 0.91 | 0.94 | 0.96 | 0.96 | 0.97 | 0.98 | 0.99 | **1.00** |
| **0.33** | **0.49** |  | 0.86 |  | 0.89 | 0.91 | 0.92 | 0.95 | 0.97 | 0.98 | 0.98 | **1.00** | 1.01 | 1.02 |
| **0.33** | **0.5** |  | 0.87 |  | 0.90 | 0.92 | 0.94 | 0.96 | 0.98 | 0.99 | **1.00** | 1.01 | 1.02 | 1.03 |
| **0.33** | **0.51** |  | 0.88 |  | 0.91 | 0.93 | 0.95 | 0.98 | **1.00** | 1.01 | 1.01 | 1.03 | 1.04 | 1.05 |
| **0.33** | **0.52** |  | 0.89 |  | 0.92 | 0.94 | 0.96 | 0.99 | 1.01 | 1.02 | 1.03 | 1.04 | 1.06 | 1.07 |
| **0.33** | **0.53** |  | 0.89 |  | 0.92 | 0.95 | 0.97 | **1.00** | 1.03 | 1.04 | 1.04 | 1.06 | 1.07 | 1.08 |
| **0.33** | **0.54** |  | 0.90 |  | 0.93 | 0.96 | 0.98 | 1.01 | 1.04 | 1.05 | 1.06 | 1.08 | 1.09 | 1.10 |
| **0.33** | **0.55** |  | 0.91 |  | 0.94 | 0.97 | 0.99 | 1.03 | 1.05 | 1.07 | 1.07 | 1.09 | 1.11 | 1.12 |
| **0.33** | **0.56** |  | 0.92 |  | 0.95 | 0.98 | **1.00** | 1.04 | 1.07 | 1.08 | 1.09 | 1.11 | 1.12 | 1.13 |
| **0.33** | **0.57** |  | 0.92 |  | 0.96 | 0.99 | 1.01 | 1.05 | 1.08 | 1.09 | 1.10 | 1.12 | 1.14 | 1.15 |
| **0.33** | **0.58** |  | 0.93 |  | 0.97 | **1.00** | 1.03 | 1.07 | 1.10 | 1.11 | 1.12 | 1.14 | 1.15 | 1.17 |

* Prevalence of alcohol consumption at Camp Lejeune

# Prevalence of alcohol consumption at Camp Pendleton

Alcohol consumption and liver cirrhosis mortality RRs: 2.65 for 25 g/day, 6.83 for 50 g/day, and 16.38 for 100 g/day [10].

The bias analysis assumed that 33% of CL employees were moderate to heavy drinkers.

To fully explain the HR of 0.74 for chronic liver disease mortality and base location, the prevalence difference between Camp Lejeune and Camp Pendleton would range between 15% and 25% (**bolded**).

CL – Camp Lejeune

CP – Camp Pendleton

HR – hazard ratio

RR – risk ratio

**Table S27**. Oral cancer mortality (as a contributing cause) hazard ratio = 1.12. Adjusted for a 15% alcohol consumption prevalence (p) difference between the Camp Lejeune and Camp Pendleton civilian employees.

| **Multidimensional RR CL-oral cancer deaths Relationship Adjusted for alcohol use** | | | | | | | | | | | | | | |
| --- | --- | --- | --- | --- | --- | --- | --- | --- | --- | --- | --- | --- | --- | --- |
|  |  |  |  |  |  |  |  |  |  |  |  |  |  |  |
| **p(alcohol use+\|CL+)*** | **p(alcohol use+\|CL-)^#^** | **RR(alcohol use-oral cancer deaths)** | **1.1** |  | **1.5** | **2** | **2.5** | **3** | **3.5** | **4** | **4.5** | **5** | **5.1** | **5.2** |
| **0.33** | **0.45** |  | 1.13 |  | 1.18 | 1.22 | 1.25 | 1.28 | 1.30 | 1.32 | 1.34 | 1.35 | 1.35 | 1.36 |
| **0.33** | **0.46** |  | 1.13 |  | 1.18 | 1.23 | 1.26 | 1.29 | 1.32 | 1.34 | 1.35 | 1.37 | 1.37 | 1.37 |
| **0.33** | **0.47** |  | 1.13 |  | 1.19 | 1.24 | 1.28 | 1.31 | 1.33 | 1.35 | 1.37 | 1.39 | 1.39 | 1.39 |
| **0.33** | **0.48** |  | **1.13** |  | **1.19** | **1.24** | **1.29** | **1.32** | **1.35** | **1.37** | **1.39** | **1.41** | **1.41** | **1.41** |
| **0.33** | **0.49** |  | 1.14 |  | 1.20 | 1.25 | 1.30 | 1.33 | 1.36 | 1.39 | 1.41 | 1.43 | 1.43 | 1.43 |
| **0.33** | **0.5** |  | 1.14 |  | 1.20 | 1.26 | 1.31 | 1.35 | 1.38 | 1.41 | 1.43 | 1.45 | 1.45 | 1.45 |
| **0.33** | **0.51** |  | 1.14 |  | 1.21 | 1.27 | 1.32 | 1.36 | 1.39 | 1.42 | 1.45 | 1.47 | 1.47 | 1.47 |
| **0.33** | **0.52** |  | 1.14 |  | 1.21 | 1.28 | 1.33 | 1.37 | 1.41 | 1.44 | 1.46 | 1.49 | 1.49 | 1.49 |
| **0.33** | **0.53** |  | 1.14 |  | 1.21 | 1.29 | 1.34 | 1.39 | 1.43 | 1.46 | 1.48 | 1.50 | 1.51 | 1.51 |
| **0.33** | **0.54** |  | 1.14 |  | 1.22 | 1.30 | 1.35 | 1.40 | 1.44 | 1.47 | 1.50 | 1.52 | 1.53 | 1.53 |
| **0.33** | **0.55** |  | 1.14 |  | 1.22 | 1.30 | 1.37 | 1.42 | 1.46 | 1.49 | 1.52 | 1.54 | 1.55 | 1.55 |

* Prevalence of alcohol consumption at Camp Lejeune

# Prevalence of alcohol consumption at Camp Pendleton

RR for oral cancer and moderate alcohol use (12.5g/day – 50g/day) = 1.83 [12].

RR for oral cancer and heavy alcohol use (>50g/day) = 5.13 [12].

The increase in the HR for oral cancers by adjusting for a 15% difference in alcohol use prevalence between Camp Lejeune and Camp Pendleton would be from a HR of 1.12 to HRs between 1.13 and 1.41 (**bolded**).

CL – Camp Lejeune

CP – Camp Pendleton

HR – hazard ratio

RR – risk ratio

**Table S28**. Cancer of the larynx hazard ratio = 1.19. Adjusted for a 15% alcohol consumption prevalence (p) difference between the Camp Lejeune and Camp Pendleton civilian employees.

| **Multidimensional RR CL-Laryngeal cancer Relationship Adjusted for alcohol use** | | | | | | | | | | | | | | |
| --- | --- | --- | --- | --- | --- | --- | --- | --- | --- | --- | --- | --- | --- | --- |
|  |  |  |  |  |  |  |  |  |  |  |  |  |  |  |
| **p(alcohol use+\|CL+)*** | **p(alcohol use+\|CL-)^#^** | **RR(alcohol use-Laryngeal cancer)** | **1.1** |  | **1.3** | **1.5** | **1.7** | **1.9** | **2.1** | **2.3** | **2.5** | **2.7** | **2.9** | **3** |
| **0.33** | **0.44** |  | 1.20 |  | 1.22 | 1.24 | 1.26 | 1.28 | 1.29 | 1.30 | 1.32 | 1.33 | 1.34 | 1.34 |
| **0.33** | **0.45** |  | 1.20 |  | 1.22 | 1.25 | 1.27 | 1.28 | 1.30 | 1.31 | 1.33 | 1.34 | 1.35 | 1.36 |
| **0.33** | **0.46** |  | 1.20 |  | 1.23 | 1.25 | 1.27 | 1.29 | 1.31 | 1.33 | 1.34 | 1.35 | 1.37 | 1.37 |
| **0.33** | **0.47** |  | 1.20 |  | 1.23 | 1.26 | 1.28 | 1.30 | 1.32 | 1.34 | 1.35 | 1.37 | 1.38 | 1.39 |
| **0.33** | **0.48** |  | **1.20** |  | **1.23** | **1.26** | **1.29** | **1.31** | **1.33** | **1.35** | **1.36** | **1.38** | **1.39** | **1.40** |
| **0.33** | **0.49** |  | 1.20 |  | 1.24 | 1.27 | 1.29 | 1.32 | 1.34 | 1.36 | 1.38 | 1.39 | 1.41 | 1.41 |
| **0.33** | **0.5** |  | 1.20 |  | 1.24 | 1.27 | 1.30 | 1.32 | 1.35 | 1.37 | 1.39 | 1.40 | 1.42 | 1.43 |
| **0.33** | **0.51** |  | 1.21 |  | 1.24 | 1.28 | 1.31 | 1.33 | 1.36 | 1.38 | 1.40 | 1.42 | 1.43 | 1.44 |
| **0.33** | **0.52** |  | 1.21 |  | 1.25 | 1.28 | 1.31 | 1.34 | 1.37 | 1.39 | 1.41 | 1.43 | 1.45 | 1.46 |
| **0.33** | **0.53** |  | 1.21 |  | 1.25 | 1.29 | 1.32 | 1.35 | 1.38 | 1.40 | 1.42 | 1.44 | 1.46 | 1.47 |
| **0.33** | **0.54** |  | 1.21 |  | 1.25 | 1.29 | 1.33 | 1.36 | 1.39 | 1.41 | 1.43 | 1.46 | 1.48 | 1.48 |

* Prevalence of alcohol use at Camp Lejeune

# Prevalence of alcohol use at Camp Pendleton

RR for laryngeal cancer and moderate alcohol use (12.5g/day – 50g/day) = 1.44 [12].

RR for laryngeal cancer and heavy alcohol use (>50g/day) = 2.65 [12].

The increase in the HR for laryngeal cancer by adjusting for a 15% difference in alcohol use prevalence between Camp Lejeune and Camp Pendleton would be from a HR of 1.19 to HRs between 1.20 and 1.40 (**bolded**).

CL – Camp Lejeune

CP – Camp Pendleton

HR – hazard ratio

RR – risk ratio

**Table S29**. Cancer of the pharynx hazard ratio = 2.21. Adjusted for a 15% alcohol consumption prevalence (p) difference between the Camp Lejeune and Camp Pendleton civilian workers.

| **Multidimensional RR CL-pharyngeal cancer Relationship Adjusted for alcohol** | | | | | | | | | | | | | | |
| --- | --- | --- | --- | --- | --- | --- | --- | --- | --- | --- | --- | --- | --- | --- |
|  |  |  |  |  |  |  |  |  |  |  |  |  |  |  |
| **p(alcohol+\|CL+)*** | **p(alcohol+\|CL-)^#^** | **RR(alcohol-pharyngeal cancer)** | **1.1** |  | **1.5** | **2** | **2.5** | **3** | **3.5** | **4** | **4.5** | **5** | **5.1** | **5.2** |
| **0.33** | **0.45** |  | 2.24 |  | 2.32 | 2.41 | 2.48 | 2.53 | 2.57 | 2.61 | 2.64 | 2.67 | 2.67 | 2.68 |
| **0.33** | **0.46** |  | 2.24 |  | 2.33 | 2.43 | 2.50 | 2.56 | 2.60 | 2.64 | 2.68 | 2.71 | 2.71 | 2.72 |
| **0.33** | **0.47** |  | 2.24 |  | 2.34 | 2.44 | 2.52 | 2.58 | 2.63 | 2.68 | 2.71 | 2.74 | 2.75 | 2.76 |
| **0.33** | **0.48** |  | **2.24** |  | **2.35** | **2.46** | **2.54** | **2.61** | **2.66** | **2.71** | **2.75** | **2.78** | **2.79** | **2.79** |
| **0.33** | **0.49** |  | 2.24 |  | 2.36 | 2.48 | 2.57 | 2.64 | 2.70 | 2.74 | 2.79 | 2.82 | 2.83 | 2.83 |
| **0.33** | **0.5** |  | 2.25 |  | 2.37 | 2.49 | 2.59 | 2.66 | 2.73 | 2.78 | 2.82 | 2.86 | 2.87 | 2.87 |
| **0.33** | **0.51** |  | 2.25 |  | 2.38 | 2.51 | 2.61 | 2.69 | 2.76 | 2.81 | 2.86 | 2.90 | 2.90 | 2.91 |
| **0.33** | **0.52** |  | 2.25 |  | 2.39 | 2.53 | 2.63 | 2.72 | 2.79 | 2.84 | 2.89 | 2.93 | 2.94 | 2.95 |
| **0.33** | **0.53** |  | 2.25 |  | 2.40 | 2.54 | 2.65 | 2.74 | 2.82 | 2.88 | 2.93 | 2.97 | 2.98 | 2.99 |
| **0.33** | **0.54** |  | 2.26 |  | 2.41 | 2.56 | 2.68 | 2.77 | 2.85 | 2.91 | 2.96 | 3.01 | 3.02 | 3.03 |
| **0.33** | **0.55** |  | 2.26 |  | 2.42 | 2.58 | 2.70 | 2.80 | 2.88 | 2.94 | 3.00 | 3.05 | 3.06 | 3.07 |

* Prevalence of alcohol consumption at Camp Lejeune

# Prevalence of alcohol consumption at Camp Pendleton

RR for oral cancer and moderate alcohol use (12.5g/day – 50g/day) = 1.83 [12].

RR for oral cancer and heavy alcohol use (>50g/day) = 5.13 [12].

The increase in the HR for cancer of the pharynx by adjusting for a 15% difference in alcohol use prevalence between Camp Lejeune and Camp Pendleton would be from a HR of 2.21 to HRs between 2.24 and 2.79 (**bolded**).

CL – Camp Lejeune

CP – Camp Pendleton

HR – hazard ratio

RR – risk ratio

**Table S30**. Female breast cancer mortality hazard ratio = 1.19. Adjusted for a 15% alcohol consumption prevalence (p) difference between the Camp Lejeune and Camp Pendleton civilian employees.

| **Multidimensional RR CL-female breast cancer Relationship Adjusted for alcohol use** | | | | | | | | | | | | | | |
| --- | --- | --- | --- | --- | --- | --- | --- | --- | --- | --- | --- | --- | --- | --- |
|  |  |  |  |  |  |  |  |  |  |  |  |  |  |  |
| **p(alcohol use+\|CL+)*** | **p(alcohol use+\|CL-)^#^** | **RR(alcohol use-female breast cancer)** | **1.1** |  | **1.15** | **1.2** | **1.25** | **1.3** | **1.35** | **1.4** | **1.45** | **1.5** | **1.55** | **1.6** |
| **0.33** | **0.44** |  | 1.20 |  | 1.20 | 1.21 | 1.22 | 1.22 | 1.23 | 1.23 | 1.24 | 1.24 | 1.25 | 1.25 |
| **0.33** | **0.45** |  | 1.20 |  | 1.21 | 1.21 | 1.22 | 1.22 | 1.23 | 1.24 | 1.24 | 1.25 | 1.25 | 1.26 |
| **0.33** | **0.46** |  | 1.20 |  | 1.21 | 1.21 | 1.22 | 1.23 | 1.23 | 1.24 | 1.25 | 1.25 | 1.26 | 1.26 |
| **0.33** | **0.47** |  | 1.20 |  | 1.21 | 1.22 | 1.22 | 1.23 | 1.24 | 1.24 | 1.25 | 1.26 | 1.26 | 1.27 |
| **0.33** | **0.48** |  | **1.20** |  | **1.21** | **1.22** | **1.23** | **1.23** | **1.24** | **1.25** | **1.26** | **1.26** | **1.27** | **1.27** |
| **0.33** | **0.49** |  | 1.20 |  | 1.21 | 1.22 | 1.23 | 1.24 | 1.24 | 1.25 | 1.26 | 1.27 | 1.27 | 1.28 |
| **0.33** | **0.5** |  | 1.20 |  | 1.21 | 1.22 | 1.23 | 1.24 | 1.25 | 1.26 | 1.26 | 1.27 | 1.28 | 1.29 |
| **0.33** | **0.51** |  | 1.21 |  | 1.22 | 1.23 | 1.23 | 1.24 | 1.25 | 1.26 | 1.27 | 1.28 | 1.28 | 1.29 |
| **0.33** | **0.52** |  | 1.21 |  | 1.22 | 1.23 | 1.24 | 1.25 | 1.26 | 1.26 | 1.27 | 1.28 | 1.29 | 1.30 |
| **0.33** | **0.53** |  | 1.21 |  | 1.22 | 1.23 | 1.24 | 1.25 | 1.26 | 1.27 | 1.28 | 1.29 | 1.30 | 1.30 |
| **0.33** | **0.54** |  | 1.21 |  | 1.22 | 1.23 | 1.24 | 1.25 | 1.26 | 1.27 | 1.28 | 1.29 | 1.30 | 1.31 |

* Prevalence of alcohol consumption at Camp Lejeune

# Prevalence of alcohol consumption at Camp Pendleton

RR for female breast cancer and moderate alcohol use (12.5g/day – 50g/day) = 1.23 [12].

RR for female breast cancer and heavy alcohol use (>50g/day) = 1.61 [12].

The increase in the HR for female breast cancer by adjusting for a 15% difference in alcohol use prevalence between Camp Lejeune and Camp Pendleton would be from a HR of 1.19 to HRs between 1.20 and 1.27 (**bolded**).

CL – Camp Lejeune

CP – Camp Pendleton

HR – hazard ratio

RR – risk ratio

**Table S31**: Increases in the hazard ratio (HR) accounting for non-differential exposure misclassification: Marines/Navy personnel subgroup

| Sensitivity | Specificity | % false positive | Kidney cancer | Esophageal cancer | Lung cancer | Parkinson disease |
| --- | --- | --- | --- | --- | --- | --- |
|  |  |  | HR=1.21^£^ | HR=1.24^£^ | HR=1.18^£^ | HR=2.05^£^ |
| 1.00 | 0.91 | 10% | 1.23 | 1.27 | 1.20 | 2.17 |
| 1.00 | 0.875 | 15% | 1.24 | 1.28 | 1.21 | 2.24 |
| 1.00 | 0.84 | 20% | 1.26 | 1.30 | 1.22 | 2.32 |
| 1.00 | 0.81 | 25% | 1.27 | 1.32 | 1.23 | 2.40 |

HR: adjusted hazard ratio

£: Adjusted HR from Table 3 (underlying cause)

**Table S32**. Increases in the hazard ratio (HR) accounting for non-differential exposure misclassification: Civilian employees

| Sensitivity | Specificity | % false positive | Lung cancer | Kidney cancer | Kidney cancer* | Female breast cancer | Female breast cancer* | Parkinson disease | Chronic kidney disease |
| --- | --- | --- | --- | --- | --- | --- | --- | --- | --- |
|  |  |  | HR=1.13^£^ | HR=1.44^£^ | HR=1.12^∞^ | HR=1.19^£^ | HR=1.33^∞^ | HR=1.21^£^ | HR=1.88^£^ |
| 1.00 | 0.91 | 10% | 1.14 | 1.53 | 1.13 | 1.21 | 1.36 | 1.24 | 1.97 |
| 1.00 | 0.875 | 15% | 1.14 | 1.55 | 1.13 | 1.21 | 1.37 | 1.25 | 2.02 |
| 1.00 | 0.84 | 20% | 1.15 | 1.59 | 1.14 | 1.22 | 1.39 | 1.26 | 2.07 |
| 1.00 | 0.81 | 25% | 1.16 | 1.62 | 1.15 | 1.23 | 1.41 | 1.27 | 2.13 |

HR: adjusted hazard ratio

£: Adjusted HR from Table 6 (underlying cause)

∞: Adjusted HR from Table 7 (contributing cause)

* Adjusted HR for contributing cause

**References**

1. Fox MP, MacLehose RF, Lash TL. Applying Quantitative Bias Analysis to Epidemiologic Data, Second Edition Springer (NY, 2021).
2. Bray RM and Hourani LL. Substance use trends among active duty military personnel: findings from the United States Department of Defense Health Related Behavior Surveys, 1980–2005. Addiction 2007;102:1092–1101. PMID: 17567397 https://doi.org/ 10.1111/j.1360-0443.2007.01841.x.
3. Forey BA, Thornton AJ, Lee PN. Systematic review with meta-analysis of the epidemiological evidence relating smoking to COPD, chronic bronchitis and emphysema. BMC Pulm Med 2011;11:36. PMID: 21672193 <https://doi.org/10.1186/1471-2466-11-36>.
4. Gandini S, Botteri E, Iodice S, Boniol M, Lowenfels AB, Maisonneuve P, Boyle P. Tobacco smoking and cancer: a meta-analysis. Int J Cancer 2008;122:155-164. PMID: 17893872 <https://doi.org/10.1002/ijc.23033>.
5. Cumberbatch MG, Rota M, Catto JWF, La Vecchia C. The role of tobacco smoke in bladder and kidney carcinogenesis: A comparison of exposures and meta-analysis of incidence and mortality risks. Eur Urol 2016;70:458-466. PMID: 26149669 https://doi.org/10.1016/j.eururo.2015.06.042.
6. Luu MN, Han M, Bui TT, Tran PTT, Lim MK, Oh JK. Smoking trajectory and cancer risk: A population-based cohort study. Tob. Induc. Dis. 2022;20(August):71. PMID: 36118557 https://doi.org/10.18332/tid/152137
7. Mappin-Kasirer B, Pan H, Lewington S, Kizza J, Gray R, Clarke R, Peto R. Tobacco smoking and the risk of Parkinson disease: A 65-year follow-up of 30,000 male British doctors. Neurology 2020;94:e2132-e2138. PMID: 32371450 https://doi.org/10.1212/WNL.0000000000009437
8. Ritz B, Ascherio A, Checkoway H, Marder KS, Nelson LM, Rocca WA, et al. Pooled analysis of tobacco use and risk of Parkinson disease. Arch Neurol 2007;64:990-7. PMID: 17620489 <https://doi.org/10.1001/archneur.64.7.990>
9. Carter BD, Abnet CC, Feskanich D, Freedman ND, Hartge P, Lewis CE, et al. Smoking and mortality – beyond established causes. NEJM 2015;372:631-40. PMID: 25671255 https://doi.org/10.1056/NEJMsa1407211
10. Llamosas-Falcon L, Probst C, Buckley C, Jiang H, Lasserre AM, Puka K, et al. How does alcohol use impact morbidity and mortality of liver cirrhosis? A systematic review and dose-response meta-analysis. Hepatology International 08 September 2023 online ahead of print. PMID: 37684424 <https://doi.org/10.1007/s12072-023-10584-z>.
11. Kunzmann AT, Coleman HG, Huang WY, Berndt SI. The association of lifetime alcohol use with mortality and cancer risk in older adults: A cohort study. PLoS Med 2018;15(6): e1002585 (Supplemental table 3). PMID: 29920516 <https://doi.org/10.1371/journal.pmed.1002585>.
12. Rumgay H, Murphy N, Ferrari P, Soerjomatatum I. Alcohol and cancer: Epidemiology and biological mechanisms. Nutrients 2021;13:3173 PMID: 34579050 https://doi.org/ 10.3390/nu13093173.
